# The mechanism of BUD13 m6A methylation mediated MBNL1-phosphorylation by CDK12 regulating the vasculogenic mimicry in glioblastoma cells

**DOI:** 10.1038/s41419-022-05426-z

**Published:** 2022-12-03

**Authors:** Meichen Liu, Xuelei Ruan, Xiaobai Liu, Weiwei Dong, Di Wang, Chunqing Yang, Libo Liu, Ping Wang, Mengyang Zhang, Yixue Xue

**Affiliations:** 1 Department of Neurobiology, School of Life Sciences, China Medical University, 110122 Shenyang, China; 2 Department of Neurosurgery, Shengjing Hospital of China Medical University, 110004 Shenyang, China; 3 Key Laboratory of Neuro-oncology in Liaoning Province, 110004 Shenyang, China; 4 Liaoning Medical Surgery and Rehabilitation Robot Technology Engineering Research Center, 110004 Shenyang, China

**Keywords:** CNS cancer, Cell growth

## Abstract

Vasculogenic mimicry (VM) is an endothelium-independent tumor microcirculation that provides adequate blood supply for tumor growth. The presence of VM greatly hinders the treatment of glioblastoma (GBM) with anti-angiogenic drugs. Therefore, targeting VM formation may be a feasible therapeutic strategy for GBM. The research aimed to evaluate the roles of BUD13, CDK12, MBNL1 in regulating VM formation of GBM. BUD13 and CDK12 were upregulated and MBNL1 was downregulated in GBM tissues and cells. Knockdown of BUD13, CDK12, or overexpression of MBNL1 inhibited GBM VM formation. METTL3 enhanced the stability of BUD13 mRNA and upregulated its expression through m6A methylation. BUD13 enhanced the stability of CDK12 mRNA and upregulated its expression. CDK12 phosphorylated MBNL1, thereby regulating VM formation of GBM. The simultaneous knockdown of BUD13, CDK12, and overexpression of MBNL1 reduced the volume of subcutaneously transplanted tumors in nude mice and prolonged the survival period. Thus, the BUD13/CDK12/MBNL1 axis plays a crucial role in regulating VM formation of GBM and provides a potential target for GBM therapy.

## Introduction

Glioblastoma (GBM) is one of the most common primary malignant tumors in the central nervous system [1]. Despite continuous improvement in treatment, the prognosis of GBM remains poor due to the special location and high invasiveness [2], with a median survival of only 15 months. Vasculogenic mimicry (VM) is a new phenomenon about fluid-conducting channels discovered by Maniotis [3]. VM microcirculatory channels lined by nonendothelial cells are generated by pluripotent embryonic stem cells, highly invasive tumor cells and the extra-cellular matrix in aggressive primary and metastatic tumors. VM mimics the function of blood vessels and transports plasma and blood cells, providing an alternative mechanism to supply malignant tumors with adequate blood. The pathological grade of glioma increases with the number of VM [4]. The presence of VM greatly hinders the treatment of GBM with anti-angiogenic drugs [5]. Therefore, it is of great significance to research the inhibition of VM formation in GBM for gene targeting therapy.

RNA-binding proteins (RBPs) form ribonucleoprotein complexes by binding to double-stranded RNA through RNA structural domains [6] to regulate cell metabolism, proliferation, and differentiation. Previous studies have found that some RBPs act as oncogenes in lung cancer [7], and liver cancer [8], however, some others act as tumor suppressors in breast cancer [9] and liver cancer [10].

BUD13 homolog (BUD13) is the subunit of the retention and splicing (RES) complex, which affects the retention of pre-mRNA in yeast cells [11]. BUD13 regulates blood lipid metabolism, and its aberrant expression causes kidney disease [12], coronary artery disease [13], and metabolic syndrome [14]. However, the effect of BUD13 on GBM cells remains unknown.

N6-methyladenosine (m6A) methylation mainly occurs on adenosine of the RRACH (R: purine; A: m6A; H: non-guanine) sequence [15]. The m6A methylation regulates the whole process of RNA processing, such as nuclear export, translation, degradation, etc [16]. Methyltransferase like 3 (METTL3), one of the most common RNA methyltransferases, is widely distributed in human eukaryotic cells [17] and regulates the proliferation, migration, invasion, and progression of various tumors. Research showed METTL3 regulated m6A methylation of RBP-HBx [18]. The m6A2Target database and m6A SRAMP database predicted the existence of m6A methylation sites on BUD13 mRNA. Therefore, METTL3 might regulate the malignant progression of tumors via m6A methylation of BUD13. Upon searching, studies of METTL3 regulating the m6A methylation of BUD13 in GBM have not been reported.

Cyclin dependent kinase 12 (CDK12) is a member of the serine/threonine kinase family. It regulates a variety of biological processes, such as C-Myc expression, Wnt/β-catenin signaling pathway, RNA splicing, MAPK signaling pathway, and DNA damage response [19]. CDK12 is upregulated and promotes proliferation and migration in prostate cancer [20], breast cancer [21], and liver cancer [22]. To date, the research on CDK12 in GBM has not been reported. STARBASE database predicted that BUD13 and CDK12 mRNA have potential binding sites. RBP-RBM47 has been reported to inhibit non-small cell lung cancer metastasis by regulating AXIN1 mRNA stability [23]. Whether BUD13 regulates the malignant progression of GBM by regulating the stability of CDK12 mRNA still needs further investigate.

Muscleblind-like 1 (MBNL1) is a highly conserved protein belonging to the tissue-specific RNA metabolism regulation family, which controls RNA shearing function and drives large transcriptome changes during cell differentiation [24]. By generalized analysis, we found that MBNL1 was downregulated in breast cancer, leukemia, gastric cancer, esophageal cancer, GBM, and Huntington’s disease. MBNL1-AS1, an antisense protein of MBNL1, inhibits colorectal cancer, non-small cell lung cancer, and gastric cancer [25]. IPTMnet database and iGPS database predicted that MBNL1 has multiple CDK12 phosphorylation sites. We speculated that CDK12 may function by phosphorylating MBNL1 in GBM.

Matrix metallopeptidase 2 (MMP2) belongs to the matrix metalloproteinase family, which can degrade the tumor-mediated extracellular matrix. It plays an important role in the invasion and metastasis of tumor cells and promotes tumor cells VM formation through a variety of mechanisms [26, 27]. In glioma cells, high expression of MMP2 suggests an enhanced VM formation ability [5]. Laminin subunit gamma 2 (LAMC2) is a family of extracellular matrix glycoproteins. It is the main non-collagen component of the basement membrane and is involved in regulating a variety of biological processes, including cell adhesion, differentiation, migration, signaling, neurite growth [28]. LAMC2 plays a key role in VM formation of glioma through the AKT and ERK (extracellular regulated protein kinases) signaling pathways, and it increases the malignancy degree of glioma [29].

In the study, we firstly clarified the expression of BUD13, CDK12, and MBNL1 in GBM tissues and cells and further analyzed the regulatory relationship between the above factors on VM formation in GBM cell. This study aims to identify new therapeutic targets for GBM and bring new references for treatment and prognosis.

## Materials and methods

### Clinical specimens and cells

Normal brain tissues (NBTs) of patients (*n* = 9) with traumatic brain trauma in neurosurgery at Shengjing Hospital affiliated to China Medical University were selected as negative control group, and tissues with postoperative pathological examination as glioma in patients with brain tumors were selected as experimental group. Glioma are classified according to the WHO classification into low-grade (WHO 1–2, *n* = 9) and high-grade glioma (WHO 3–4, *n* = 9). This study was confirmed by the Ethics Committee of Shengjing Hospital affiliated to China Medical University, and was approved by the patients and the families with informed consent obtained. Glioma inclusion criteria: (1) age superior to 18 years; (2) first onset, imaging (CT, MRI) identified intracranial mass lesions, hospitalization for surgical treatment and postoperative pathology diagnosis; (3) without radiotherapy, chemotherapy, and other treatments before surgery. Glioma exclusion criteria: (1) combined with hematologic disorders; (2) combined with other malignant tumors; (3) combined with other organs abnormal function; (4) combined with immune system diseases or connective tissue lesions. Negative control group inclusion criteria: (1) brain trauma; (2) without related diseases such as intracranial tumors, cerebral hemorrhage, and cerebral infarction before injury. Negative control group exclusion criteria: (1) other serious underlying diseases such as: coronary heart disease, cirrhosis, renal insufficiency; (2) other tumors. All the tissue samples were immediately frozen in liquid nitrogen after surgical resection and stored in liquid nitrogen until use.

Human GBM cell lines (U251, U373) and 293T cells were purchased from the Shanghai Institutes for Biological Sciences Cell Resource Center. Normal human astrocytes (NHAs) were purchased from Sciencell Laboratory in the United States.

### RNA extraction and quantitative real-time PCR

RNA expression levels in the study were detected by quantitative real-time PCR (qRT-PCR). Trizol reagent (Life Technologies, CA, USA) was used to extract RNA from various tissues and cells. The primers are displayed in Supplementary Table S1. For more details, please see Supplemental Materials and Methods.

### Cell transfection

The overexpression plasmids, knockdown plasmids, and mutant plasmids of various indicators in the study were purchased from Gene-Pharama (Gene-Pharama, Shanghai, China). For more details, please refer to Supplemental Materials and Methods. Sequence of shRNA are shown in Supplementary Table S2. Sequence of site mutation are shown in Supplementary Table S3.

### Human mRNA microarray analysis

Microarray analysis, sample preparation and microarray hybridization were operated by Aksomics Biotech (Aksomics Biotech, Shanghai, China).

### Western blot

Western blot was operated as previously described. For details of the experiment, please see Supplemental Materials and Methods.

### Cell viability assay

Cell viability assay was operated as previously described [30]. For details of the experiment, please see Supplemental Materials and Methods.

### Cell migration assay

The capacity for migration in GBM cells was observed by the HoloMonitor M4 culture system (Phase Holographic Imaging PHI AB, Lund, Sweden) in vitro. For details of the experiment, please refer to Supplemental Materials and Methods.

### Cell invasion assay

Cell invasion assay was operated as previously described [30]. For details of the experiment, please see Supplemental Materials and Methods.

### Three-dimensional tube formation assay

Three-dimensional tube formation assay was operated as previously described. For details of the experiment, please see Supplemental Materials and Methods [30].

### RNA immunoprecipitation assay

RNA immunoprecipitation (RIP) assay was operated as previously described [30]. For details of the experiment, please see Supplemental Materials and Methods.

### RNA pull-down assay

RNA pull-down assay was operated as previously described [30]. For details of the experiment, please see Supplemental Materials and Methods.

### Nascent RNA capture assay

The nascent RNA capture assay was performed as previously described [30]. For details, please see Supplemental Materials and Methods.

### RNA stability measurement

Cells were cultured in the medium containing 5 µg/mL actinomycin D (Act D, NobleRyder, China). Total RNA was extracted from the cells and collected at different time points. The half-life of mRNA was detected at a certain time point compared with 0 h by qRT-PCR.

### Dot blot assay

The RNA was denatured by heating at 65 °C for 5 min and transferred to the nitrocellulose membrane (Amersham, GE Healthcare, USA) with Bio-Dot device (Bio-Rad, CA, USA). The membrane was UV-crosslinked, sealed, and incubated with m6A antibody (1:4000; Proteintech, IL, USA) at 4 °C overnight. After washing with TTST, the membrane was incubated with HRP-conjugated goat anti-mouse IgG (1:10,000; Proteintech, IL, USA) for 1.5 h. Then visualizing by enhanced chemiluminescence (Bio-Rad, CA, USA). Staining the membrane with 0.02% methylene blue diluted in 0.3 M sodium acetate to ensure consistency across groups.

### Immunofluorescence assay

Cells seeded on glass slides were fixed in 4% paraformaldehyde for 20 min and permeabilized with 0.2% TritonX-100 for 10 min and then blocked with 5% BSA for 2 h at room temperature. Next, the cell slides were incubated with primary antibodies at 4 °C overnight. Then, the cell slides were washed with PBST three times and incubated with fluorescent-conjugated secondary antibodies-Goat anti-Rabbit Alexa Fluor 488 or Goat anti-Mouse Alexa Fluor 647 (Beyotime Institute of Biotechnology, Jiangsu, China) for 2 h at room temperature away from the light. Finally, the nucleus were stained with DAPI for 5 min. Fluorescence was visualized under laser confocal microscopy.

### Co-immunoprecipitation assay

Co-immunoprecipitation (Co-IP) assay was performed using the Pierce Co-IP Kit (Thermo Fisher Scientific, MA, USA) following the manufacturer’s instructions. Cell lysates were prepared and incubated with AminoLink Plus Coupling Resin immobilized primary antibody overnight at 4 °C. Then, the samples were washed three times with 200 μL Wash Buffer and eluted with Elution Buffer for 5 min. The eluates were finally analyzed by western blot.

### GST pull-down assay

Prokaryotic expression plasmids fused with FLAG or GST tag were constructed including FLAG-CDK12, GST-MBNL1 (WT), GST-MBNL1-T6A (mut). These plasmids were transformed into Escherichia coli competent cell BL21 (Takara, Kyoto, Japan) and protein expression was induced by 1 mM IPTG (Solarbio, Beijing, China) at 25 °C and 200 rpm for 6 h. Then, cells were lysed, sonicated, and centrifuged. The proteins were purified using the BeyoMag™ anti-Flag Magnetic Beads and BeyoGold™ GST-tag Purification Resin (Beyotime Institute of Biotechnology, Jiangsu, China) according to the manufacturer’s procedure. For GST pull-down assay, purified FLAG-CDK12 was incubated with purification resin coupled with GST-MBNL1 protein at 4 °C overnight. Then protein complexes were eluted by Elution Buffer and then subjected to SDS-PAGE and analyzed by western blot.

### In vitro kinase assay

In vitro kinase assay was performed at 30 °C for 20 min containing 10 μg MBNL1 protein, 5 μg CDK12 kinase (Signalchem, BC, Canada), 50 μL kinase buffer (Cell Signaling Technology, MA, USA), 50 mM ATP (Beyotime Institute of Biotechnology, Jiangsu, China), 5 μCi [γ-32P]-ATP (PerkinElmer, MA, USA). Stop the kinase reaction with 1×SDS buffer (Beyotime Institute of Biotechnology, Jiangsu, China), and perform autoradiography after SDS-PAGE electrophoresis. The loaded amount of substrate protein was shown by coomassie brilliant blue (Solarbio, Beijing, China) staining.

### Cycloheximide chase assay

Cells were cultured in the medium containing 10 µM cycloheximide (CHX, Sigma-Aldrich, MO, USA) to inhibit protein biosynthesis. Total protein was extracted at different time points, then detected by western blot.

### CD34-PAS staining

VM was detected by CD34-PAS in xenograft tumor tissue samples. The assay was performed as reported previously [30]. For details, see Supplemental Materials and Methods.

### Tumor xenograft in nude mice

The constructed stably transfected GBM cells (U251 and U373) were used to establish xenograft models in nude mice. For more details, please see Supplemental Materials and Methods.

### Statistical analysis

The experimental data were collected and presented as mean ± SD. Student’s t-test or one-way ANOVA was used in the statistical analysis by the GraphPad Prism v8.4 (GraphPad, CA, USA). Differences between groups were regarded as significant when *P* < 0.05.

## Result

### BUD13 was upregulated in GBM tissues and cells, and knockdown of BUD13 inhibited VM formation in GBM cells

**Fig. 1:**
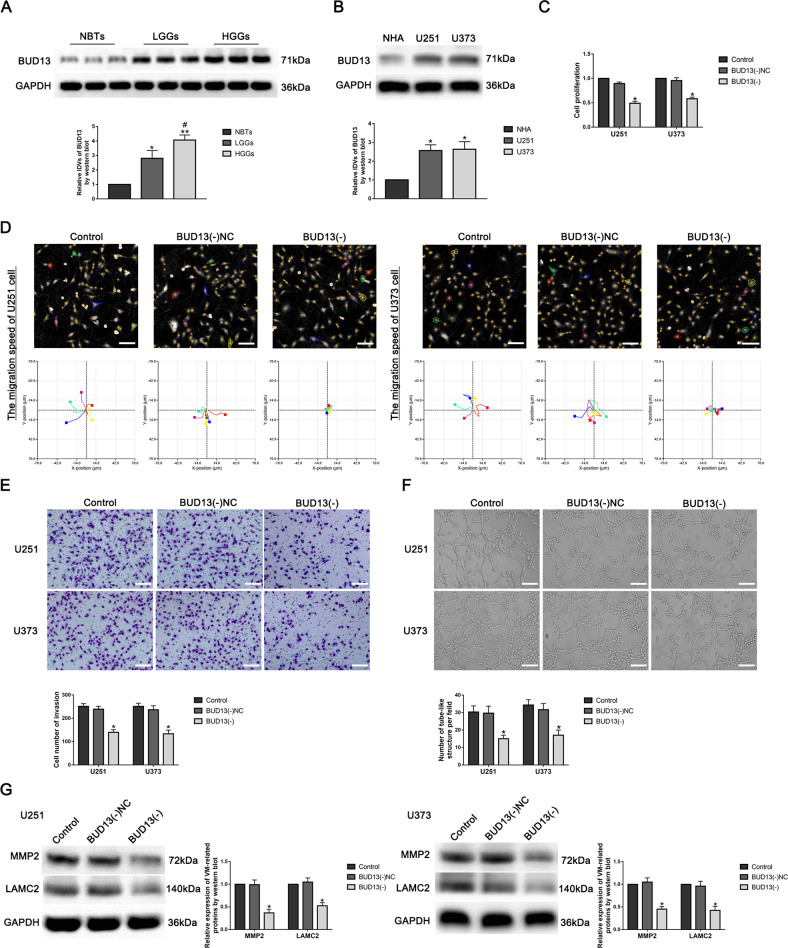
BUD13 was upregulated in GBM tissues and cells, and knockdown of BUD13 inhibited VM formation in GBM cells. **A** Western blot was used to detect the expression of BUD13 in normal brain tissues (NBTs), low-grade glioma tissues (WHO 1–2), and high-grade glioma tissues (WHO 3–4). Data are presented as mean ± SD (*n* = 3). Compared with NBTs group, ^*^P < 0.05, ^**^P < 0.01, compared with low-grade glioma tissue group, ^#^*P* < 0.05. **B** Western blot was used to detect the expression of BUD13 in normal human astrocytes (NHAs), U251, and U373 cells. Data are presented as mean ± SD (*n* = 3). ^*^P < 0.05, compared with NHAs group. **C** The effect of BUD13 on the proliferation ability of U251 and U373 cells was detected by CCK8 assay. Data are presented as mean ± SD (*n* = 3). ^*^P < 0.05, compared with BUD13(-)NC group. **D** The effect of BUD13 on the migration ability of U251 and U373 cells was detected by Hstudio M4 (*n* = 5). **E** Transwell assay was used to detect the effect of BUD13 on the invasion of U251 and U373 cells. Data are presented as mean ± SD (*n* = 3). ^*^P < 0.05, compared with BUD13(-)NC group, scale bar: 50 μm. **F** Three-dimensional tube formation assay was used to detect the effect of BUD13 on the tube formation ability of U251 and U373 cells. Data are presented as mean ± SD (*n* = 3). ^*^P < 0.05, compared with BUD13(-)NC group, scale bar: 50 μm. **G** Western blot was used to detect the effect of BUD13 on the expression of VM-related proteins MMP2 and LAMC2 in U251 and U373 cells. Data are presented as mean ± SD (*n* = 3). ^*^P < 0.05, compared with BUD13(-)NC group.

The protein expression of BUD13 in GBM tissues was significantly higher than that in NBTs and was positively correlated with the pathological grade. Similarly, BUD13 protein expression in U251 and U373 cells was significantly higher than that in NHAs (Fig. 1A, B). To further explore the role of BUD13 in GBM cells, we constructed stably knockdown of BUD13 plasmid. The proliferation, migration, invasion, and tube formation abilities in BUD13(-) group were significantly decreased compared with BUD13(-)NC group (Fig. 1C–F). The expression of VM-related proteins MMP2 and LAMC2 in BUD13(-) group decreased significantly compared with BUD13(-) NC group (Fig. 1G).

### METTL3 enhanced BUD13 mRNA stability via m6A methylating and promoted VM formation in GBM cells

The m6A methylation level and the METTL3 protein expression in U251 and U373 cells were significantly higher than that in NHAs (Fig. S1A, B). After METTL3 knockdown, the m6A methylation level reduced (Fig. S1C). Based on the m6A2Target database, METTL3 methylated BUD13 (Fig. S1D). As expected, the BUD13 protein expression decreased as METTL3 knockdown (Fig. S1E). We then confirmed that the transcript of nascent BUD13 exhibited no obvious change, whereas the half-time of BUD13 was significantly shortened after METTL3 knockdown (Fig. S1F, G). RIP assay validated the binding between METTL3 and BUD13 mRNA. As expected, the METTL3-immunoprecipitated sample showed enrichment of BUD13 mRNA compared with that of the IgG-immunoprecipitated group (Fig. S1H). Next, RNA pull-down assay validated the binding (Fig. S1I). The m6A SRAMP predicted the presence of multiple binding sites between METTL3 and BUD13 mRNA, and the 1645 site with the highest scoring was selected and mutated (Fig. S1J).

**Fig. 2:**
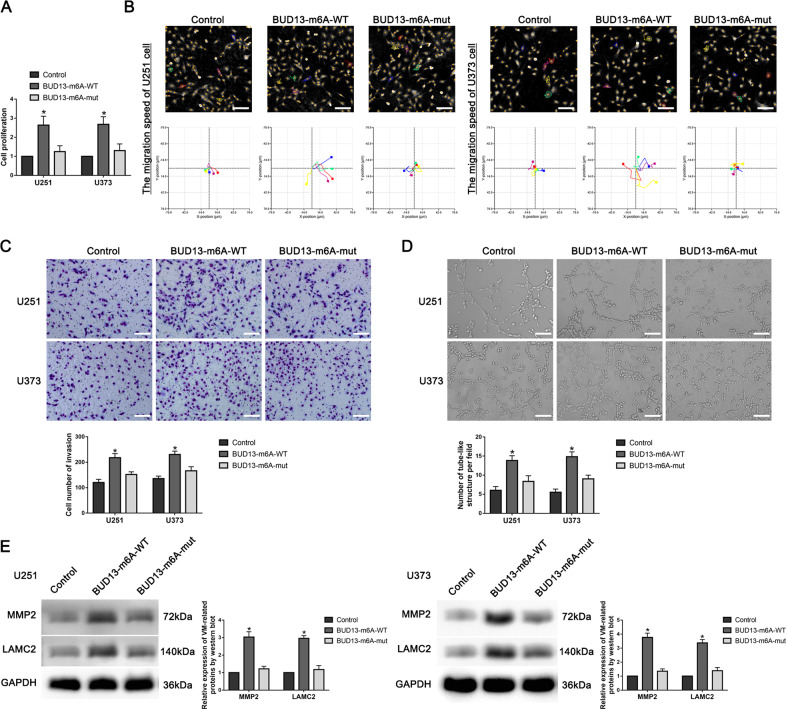
Effects of BUD13 m6A methylation site on VM formation in GBM cells. **A** The effect of BUD13 m6A methylation site on the proliferation of U251 and U373 cells was detected by CCK8 assay. Data are presented as mean ± SD (*n* = 3). ^*^P < 0.05, compared with the Control group. **B** Hstudio M4 was used to detect the effect of BUD13 m6A methylation site on the migration of U251 and U373 cells (*n* = 5). **C** Transwell assay was used to detect the effect of BUD13 m6A methylation site on the invasion of U251 and U373 cells. Data are presented as mean ± SD (*n* = 3). ^*^P < 0.05, compared with the Control group. **D** Three-dimensional tube formation assay was used to detect the effect of BUD13 m6A methylation site on the tube formation of U251 and U373 cells. Data are presented as mean ± SD (*n* = 3). ^*^P < 0.05, compared with the Control group. **E** Western blot was used to detect the effect of BUD13 m6A methylation site on the expression of VM-related proteins MMP2 and LAMC2 in U251 and U373 cells. Data are presented as mean ± SD (*n* = 3). ^*^P < 0.05, compared with the Control group.

Compared with the Control group, the proliferation, migration, invasion, and tube formation abilities of the BUD13-m6A-WT group were significantly enhanced, while BUD13-m6A-mut group had no difference (Fig. 2A–D). Compared with the Control group, VM-related proteins MMP2 and LAMC2 expression in the BUD13-m6A-WT group was significantly enhanced, whereas the BUD13-m6A-mut group had no difference (Fig. 2E). These results suggested that METTL3 strengthened the stability of BUD13 mRNA and promoted VM formation in GBM cells by methylating the 1645 site of BUD13.

### CDK12 was upregulated in GBM tissues and cells, and knockdown of CDK12 inhibited VM formation in GBM cells

**Fig. 3:**
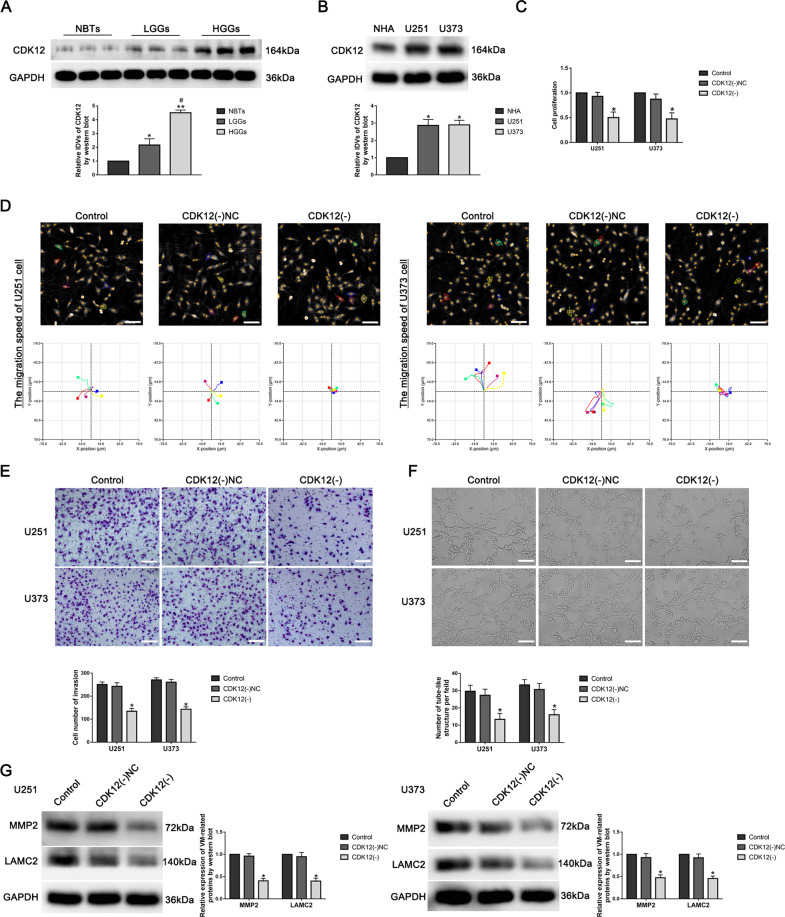
CDK12 was upregulated in GBM tissues and cells, and knockdown of CDK12 inhibited VM formation in GBM cells. **A** Western blot was used to detect CDK12 protein expression level in normal brain tissues (NBTs), low-grade glioma tissues (WHO 1–2), and high-grade glioma tissues (WHO 3–4). Data are presented as mean ± SD (*n* = 3). ^*^P < 0.05, ^**^P < 0.01, compared with NBTs group, ^#^*P* < 0.05, compared with low-grade glioma tissue group. **B** Western blot was used to detect CDK12 protein expression level in NHAs, U251, and U373 cells. Data are presented as mean ± SD (*n* = 3). ^*^P < 0.05, compared with NHAs group. **C** The effect of CDK12 on the proliferation of U251 and U373 cells was detected by CCK8 assay. Data are presented as mean ± SD (*n* = 3). ^*^P < 0.05, compared with CDK12(-)NC group. **D** Hstudio M4 was used to detect the effect of CDK12 on the migration of U251 and U373 cells (*n* = 5). **E** Transwell assay was used to detect the effect of CDK12 on the invasion of U251 and U373 cells. Data are presented as mean ± SD (*n* = 3). ^*^P < 0.05, compared with CDK12(-)NC group, scale bar: 50 μm. **F** Three-dimensional tube formation assay was used to detect the effect of CDK12 on the tube formation ability of U251 and U373 cells. Data are presented as mean ± SD (*n* = 3). ^*^P < 0.05, compared with CDK12(-)NC group, scale bar: 50 μm. **G** Western blot was used to detect the effect of CDK12 on VM-related proteins MMP2 and LAMC2 expression in U251 and U373 cells. Data are presented as mean ± SD (*n* = 3). ^*^P < 0.05, compared with CDK12(-)NC group.

With mRNA microarray analysis and qRT-PCR, we verified that CDK12 was significantly decreased in U251 and U373 cells treated with BUD13(-) (Fig. S2A–C). The protein expression of CDK12 in GBM tissues was significantly higher than that in NBTs, and was positively correlated with the pathological grade. Similarly, CDK12 protein expression in U251 and U373 cells was significantly higher than that in NHAs (Fig. 3A, B). To further explore the role of CDK12, we constructed CDK12-knockdown U251 and U373 cell lines. CDK12(-) group had significantly lower proliferation, migration, invasion, and tube formation abilities than the CDK12(-)NC group (Fig. 3C–F). VM-related proteins MMP2 and LAMC2 expression in CDK12(-) group was significantly lower than that in CDK12(-)NC group (Fig. 3G).

### BUD13 bound to CDK12 mRNA to enhance its stability and promoted VM formation in GBM cells

We further confirmed CDK12 protein expression decreased after BUD13 knockdown (Fig. S2D). STARBASE database predicted that BUD13 was positively correlated with CDK12 expression, and BUD13 might bind to CDK12 mRNA (Fig. S2E). RIP and RNA pull down assay confirmed the binding between BUD13 and CDK12 mRNA (Fig. S2,F G). After BUD13 knockdown, CDK12 nascent RNA had no obvious change, however, CDK12 half-life reduced significantly (Fig. S2H, I).

To further investigated whether CDK12 was involved in the inhibitory effect of BUD13 knockdown on VM formation in GBM cells, CDK12(-), CDK12(+), and their NC plasmids were transfected into BUD13-knockdown plasmids in the study. CDK12 knockdown strengthened the inhibition of the proliferation, migration, invasion, and tube formation abilities induced by BUD13 knockdown, while CDK12 overexpression rescued the inhibition of the proliferation, migration, invasion, and tube formation abilities induced by BUD13 knockdown (Fig. S3A–D). And we observed same effects on VM-related proteins MMP2 and LAMC2 expression (Fig. S3E). Above results confirmed that BUD13 enhanced CDK12 mRNA’s stability and promoted VM formation in GBM cells by binding to CDK12 mRNA.

### MBNL1 was downregulated in GBM tissues and cells, and overexpression of MBNL1 inhibited VM formation in GBM cells

**Fig. 4:**
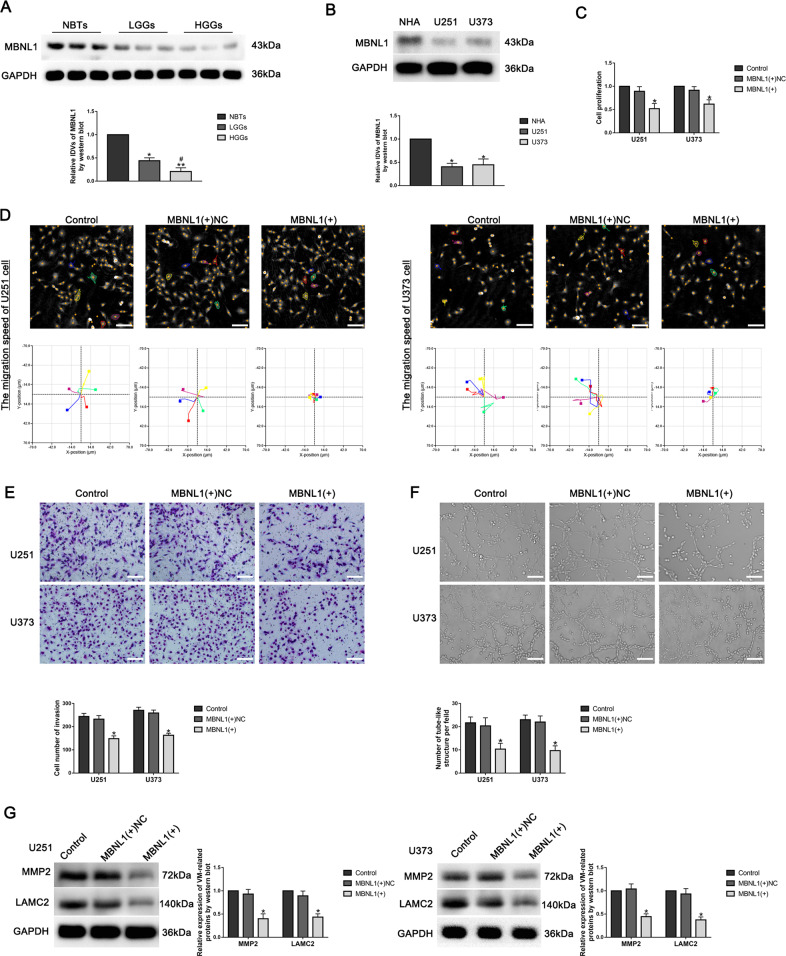
MBNL1 was downregulated in GBM tissues and cells, and overexpression of MBNL1 inhibited VM formation in GBM cells. **A** Western blot detected MBNL1 protein expression level in normal brain tissues (NBTs), low-grade glioma tissues (WHO 1–2) and high-grade glioma tissues (WHO 3–4). Data are presented as mean ± SD (*n* = 3). ^*^P < 0.05, ^**^P < 0.01, compared with NBTs group; ^#^*P* < 0.05, compared with low-grade glioma tissue group. **B** Western blot detected MBNL1 protein expression level in NHAs, U251, and U373 cells. Data are presented as mean ± SD (*n* = 3). ^*^P < 0.05, compared with NHAs group. **C** The effect of MBNL1 on the proliferation of U251 and U373 cells was detected by CCK8 assay. Data are presented as mean ± SD (*n* = 3). ^*^P < 0.05, compared with MBNL1(+)NC group. **D** Hstudio M4 detected the effect of MBNL1 on the migration of U251 and U373 cells (*n* = 5). **E** Transwell assay was used to detect the effect of MBNL1 on the invasion of U251 and U373 cells. Data are presented as mean ± SD (*n* = 3). ^*^P < 0.05, compared with MBNL1(+)NC group. Scale bar: 50 μm. **F** Three-dimensional tube formation assay detected the effect of MBNL1 on the tube formation of U251 and U373 cells. Data are presented as mean ± SD (*n* = 3). ^*^P < 0.05, compared with MBNL1(+)NC group. Scale bar: 50 μm. **G** Western blot detected the effect of MBNL1 on the expressions of VM-related proteins MMP2 and LAMC2 in U251 and U373 cells. Data are presented as mean ± SD (*n* = 3). ^*^P < 0.05, compared with MBNL1(+)NC group.

To reveal the function of CDK12, CDK12-interacting proteins were coimmunoprecipitated for mass spectrometry analysis in U251 cells expressing FLAG-CDK12. Among the candidates, MBNL1 was selected for further study (Fig. S4A). The phosphorylation sites of MBNL1 by CDK12 was based on IPTMnet database and iGPS database (Fig. S4B, C). The protein expression of MBNL1 in GBM tissues was significantly lower than that in NBTs, and was negatively correlated with the pathological grade. Similarly, the protein expression of MBNL1 in U251 and U373 cells was significantly lower than that in NHAs (Fig. 4A,B). To further explore the function of MBNL1 in GBM cells, we overexpressed MBNL1. Then we found the proliferation, migration, invasion, and tube formation abilities in MBNL1(+) group were significantly lower than that in MBNL1(+)NC group (Fig. 4C–F), and the expression of VM-related proteins MMP2 and LAMC2 in MBNL1(+) group was significantly lower than that in MBNL1(+)NC group (Fig. 4G).

### Phosphorylation of MBNL1 by CDK12 promoted VM formation in GBM cells

**Fig. 5:**
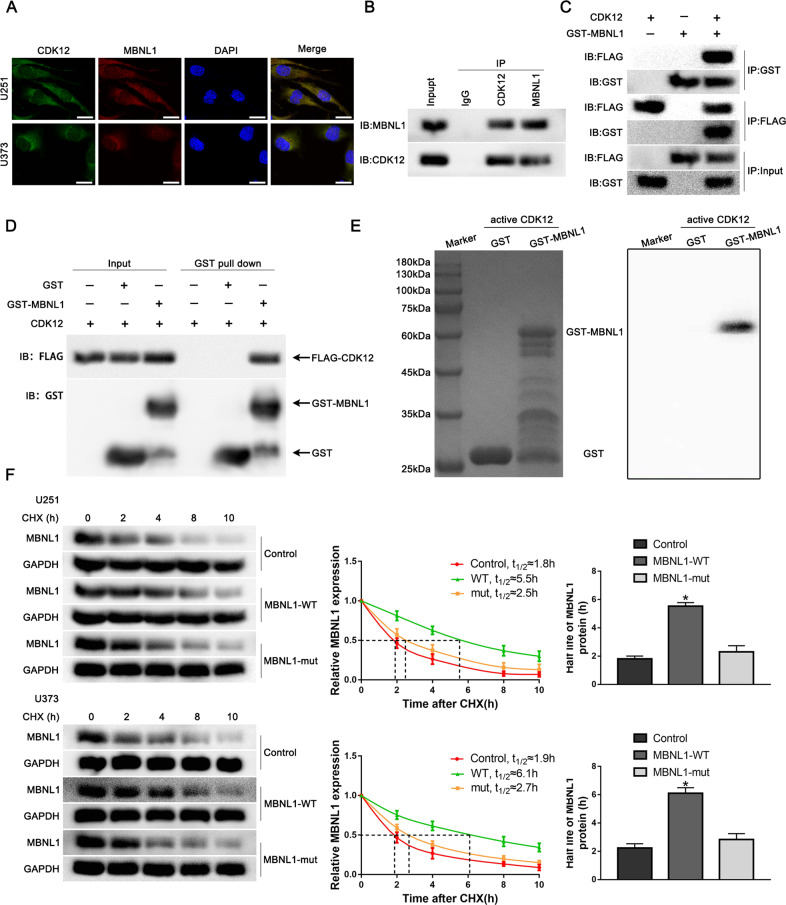
CDK12 phosphorylated MBNL1. **A** The colocalization of CDK12 and MBNL1 in U251 and U373 cells by immunofluorescence assay. Green, CDK12; red, MBNL1; blue, DAPI nuclear staining. Scale bar: 10 μm. **B** Lysates of U251 cells were subjected to immunoprecipitation (IP) and immunoblotting (IB) with CDK12 and MBNL1 antibodies. **C** Lysates of 293T cells transfected with FLAG-CDK12 and GST-MBNL1 plasmids were subjected to IP and IB with FLAG tag and GST tag antibodies. **D** The direct interaction between CDK12 and MBNL1 was confirmed by GST pull-down assay. GST protein functioned as a negative control. **E** Autoradiography detected phosphorylation of GST-MBNL1 fusion protein. **F** The effects of MBNL1 T6 phosphorylation site on its stability were detected by Cycloheximide (CHX) chase assay. Data are presented as the mean ± SD (*n* = 3, each group). ^*^P < 0.05, ^**^P < 0.01, compared with MBNL1-WT group by one-way ANOVA.

To further investigate the mechanism between CDK12 and MBNL1, immunofluorescence assay was performed and showed that CDK12 colocalized with MBNL1 in the cytoplasm of U251 and U373 cells (Fig. 5A). Co-IP assay indicated that CDK12 bound to MBNL1 in U251 cells and FLAG-CDK12 bound to GST-MBNL1 in 293T cells (Fig. 5B, C). GST pull-down assay using purified FLAG-CDK12 and GST-MBNL1 proteins proved that CDK12 directly bound to MBNL1 in vitro (Fig. 5D). In vitro kinase assay revealed that CDK12 phosphorylated MBNL1 protein (Fig. 5E). The highest scoring T6 phosphorylation site was mutated according to IPTMnet database and iGPS database. We mutated the T6 phosphorylation site rendering it unable to be phosphorylated, and found the phosphorylated band disappeared (Fig. S4D). MBNL1 and p-MBNL1 protein expression was significantly decreased in CDK12(+) group compared with CDK12(+) NC group, and MBNL1 and p-MBNL1 protein expression in CDK12(+) + MBNL1-WT group was elevated compared with CDK12(+) group. MBNL1 protein expression in CDK12(+) + MBNL1-mut group was significantly elevated, whereas p-MBNL1 protein expression was significantly decreased compared with CDK12(+) + MBNL1-WT group (Fig. S4E). Cycloheximide chase assay found that the half-life of MBNL1-WT significantly increased. However, the half-life of MBNL1-mut has no significant change (Fig. 5F).

To further investigate whether MBNL1 was involved in the inhibitory effect of CDK12-knockdown on VM formation in GBM cells. The study transfected MBNL1(+), MBNL1(-) and their NC plasmids into CDK12-knockdown plasmids. MBNL1 overexpression strengthened the inhibition of proliferation, migration, invasion and tube formation abilities induced by CDK12 knockdown, while MBNL1 knockdown rescued the inhibition of proliferation, migration, invasion and tube formation abilities induced by CDK12 knockdown (Fig. S5A–D). And we observed similar effects on MMP2 and LAMC2 protein expression (Fig. S5E).

### Knockdown of BUD13 and CDK12 combined with overexpression of MBNL1 inhibited the growth of GBM and prolonged the survival period of nude mice

**Fig. 6:**
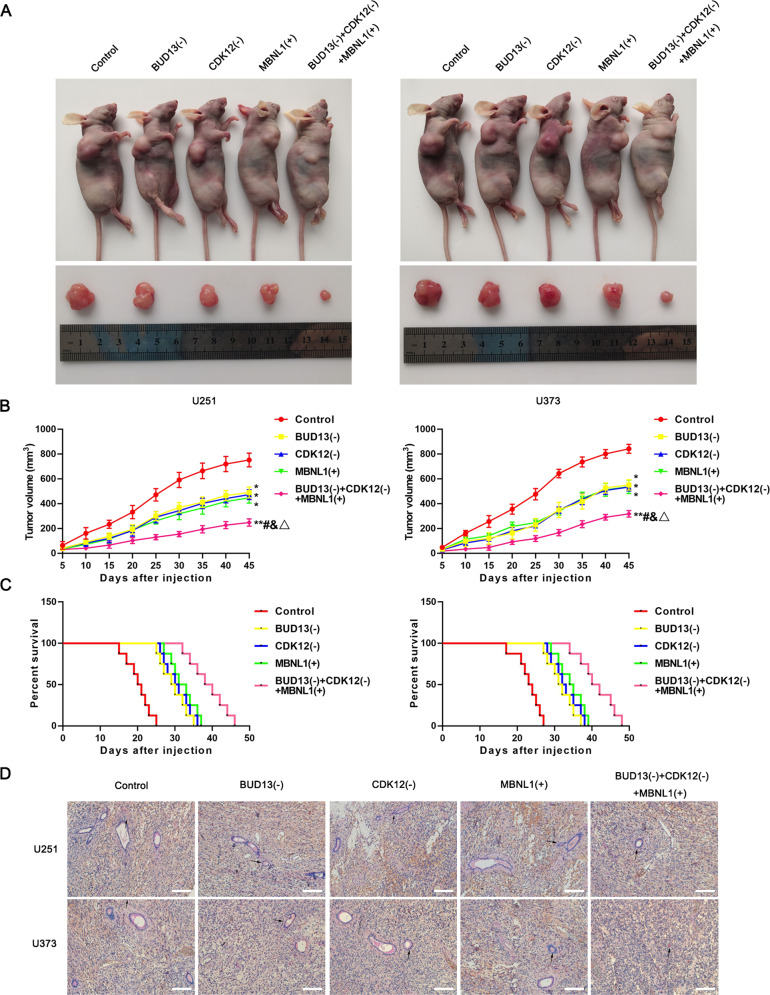
Knockdown of BUD13 and CDK12 combined with overexpression of MBNL1 inhibited the growth of GBM and prolonged the survival period of nude mice. **A** Subcutaneously xenografted nude mice injected with differently treated cells are shown (above). Representative tumors from each group are shown (below). **B** Tumor growth curve (*n* = 8). Tumor size was recorded every 5 days and tumors were removed after day 45. ^*^P < 0.05, ^**^P < 0.01, compared with the Control group; ^#^*P* < 0.05, compared with BUD13(-) group; ^&^*P* < 0.05, compared with CDK12(-) group; ^Δ^*P* < 0.05, compared with MBNL1(+) group. **C** Survival curve of subcutaneously transplanted tumor in nude mice (*n* = 8). **D** CD34-PAS staining was used to detect tube-forming ability in nude mice tumors. Scale bar: 50 μm. Arrows indicate VM structures.

**Fig. 7:**
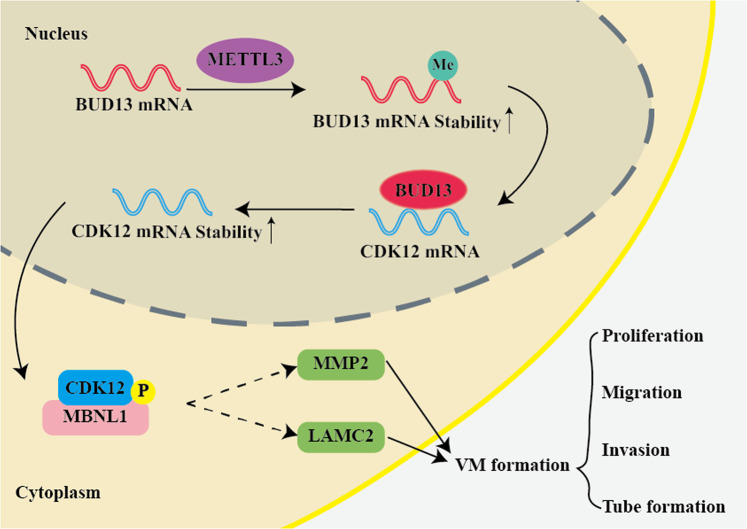
The schematic diagram about the regulating process of BUD13/CDK12/MBNL1 axis on VM formation of GBM. METTL3 enhanced the stability of BUD13 mRNA and upregulated its expression through m6A methylation. BUD13 enhanced the stability of CDK12 mRNA and upregulated its expression. CDK12 phosphorylating MBNL1 regulated proliferation, migration, invasion, and tube formation of GBM.

To confirm the roles of BUD13, CDK12, and MBNL1 in tumor growth in vivo, we subcutaneously injected GBM BUD13(-) cells, CDK12(-) cells, MBNL1(+) cells, or a combination of the three cells to construct nude mice xenograft models. The average size of the xenograft was smaller in BUD13(-), CDK12(-), and MBNL1(+) mice than that in the Control mice (Fig. 6A, B). The xenograft volume was smallest in the group injected with a combination of the three cells. GBM cells were stereotactically implanted into the right striatum of nude mice for survival time analysis. As shown in Fig. 6C, mice in the BUD13(-), CDK12(-), and MBNL1(+) groups showed longer survival time than that in the Control group, and mice in the BUD13(-) + CDK12(-) + MBNL1(+) group showed the longest survival time. Then, pathological sections of orthotopically transplanted nude mice were taken. Meanwhile, CD34-PAS staining found that the number of VM in BUD13(-), CDK12(-), and MBNL1(+) mice was decreased compared with the Control mice, and was most obvious in a combination of the three cells (Fig. 6D). A schematic diagram underlying the function of BUD13/CDK12/MBNL1 axis in VM formation of GBM is shown in Fig. 7.

## Discussion

The study confirmed for the first time that BUD13 and CDK12 were upregulated and positively correlated with the pathological grade in GBM. MBNL1 was downregulated and negatively correlated with the pathological grade in GBM. METTL3 enhanced the stability of BUD13 mRNA and upregulated its expression through m6A methylation. BUD13 enhanced the stability of CDK12 mRNA and upregulated its expression. CDK12 phosphorylating MBNL1 regulated proliferation, migration, invasion, and tube formation of GBM.

RNA-binding proteins (RBPs) play vital roles throughout the mRNA life cycle [31]. The mechanisms of RBPs regulating tumorigenesis have attracted more and more attention in recent years [32]. BUD13 was originally discovered in yeast and affected alternative splicing of pre-mRNA [33]. The study verified that BUD13 was upregulated in GBM, and knockdown of BUD13 inhibited VM formation of GBM. Previous research found DBH-AS1 recruited BUD13 enhancing FN1 stability, and promoted cell proliferation, migration, and invasion in diffuse large B-cell lymphoma [34]. In prostate cancer, circSERPINA3 competed with miR-653-5p for binding BUD13, and regulated apoptosis, autophagy, and aerobic glycolysis [35]. The results above indicated BUD13 might play an oncogenic role in GBM.

m6A methylation was first discovered in the 1970s and was the most common reversible epigenetic modification in mRNA [36]. As an important member of m6A methylation regulators, METTL3 is upregulated in osteosarcoma [37], melanoma [38], liver cancer [39], and colorectal cancer [40]. The present study suggested that m6A methylation level and the expression of METTL3 in GBM cells were significantly elevated than that in NHAs, which was consistent with previous studies [41]. Next, we found that knockdown of METTL3 reduced the protein expression and m6A methylation level of BUD13. And m6A2 Target database predicting the existence of multiple m6A methylation sites in BUD13 confirmed the relationship between METTL3 and BUD13. RNA nascent assay and half-life assay found that the stability of BUD13 mRNA decreased after METTL3 knockdown, suggesting that METTL3 might enhance BUD13 mRNA’s stability by m6A methylation. RIP and RNA pull-down assay further confirmed that METTL3 bound to BUD13 mRNA. To further verify the interaction between METTL3 and BUD13, we predicted the existence of multiple m6A methylation sites between METTL3 and BUD13 through the m6A SRAMP database, and selected the 1645 site with the highest score as the target and mutated it. After mutation, the proliferation, migration, invasion, and tube formation abilities of GBM cells decreased, suggesting that METTL3 might promote GBM progression by enhancing BUD13’s stability via methylation at 1645 site.

Based on mRNA microarray analysis, we verified that CDK12 was significantly decreased in U251 and U373 cells after BUD13 knockdown. The study proved that CDK12 was upregulated in GBM tissues and cells, and knockdown of CDK12 might inhibit the proliferation, migration, invasion, and tube formation of GBM cells. CDK12 regulated transcription by binding to cycling K and phosphorylated RNA polymerase II Ser5 in vitro to regulate transcription initiation and was involved in DNA damage response, cell proliferation, and pre-RNA splicing. In diffuse large B-cell lymphoma, CDK12 activated MYC to inhibit miR-28-5p/EZH2 and amplify BCR signaling to promote its progression [42]. Structure-activity relationship study of THZ531 derivatives found that CDK12/13 dual inhibitor BSJ-01-175 could treat Ewing’s sarcoma [43]. CDK12 promoted cervical cancer progression by enhancing macrophage infiltration [44]. Knockdown of CDK12 inhibited the proliferation of lung cancer and esophageal cancer cells [45]. Targeting CCNK/CDK12 degradation might regulated colorectal cancer [46]. The above results further indicated that CDK12 might play an oncogenic role in GBM.

The STARBASE database predicted that BUD13 bound to CDK12 mRNA. In the study, RIP and RNA pull-down assay confirmed the prediction. The nascent RNA and half-life experiments confirmed the stability of CDK12 mRNA decreased when BUD13 was knockdown, suggesting that BUD13 might intensify the stability of CDK12 mRNA. Previous research reported RBPs enhanced the mRNA stability or degradation of proto-oncogenes and tumor suppressor genes by forming complexes with them [47]. For example, SORBS2 inhibited renal clear cell carcinoma metastasis by enhancing MTUS1 mRNA stability [48]; RBM38 increased PREN stability and enhanced its expression to promote breast cancer progression progress [49]. The results above suggested that BUD13 might regulate VM formation of GBM cells by enhancing the stability of CDK12.

To investigate the possible mechanisms by which CDK12 regulated VM formation in GBM, we performed Co-IP assay coupled with mass spectrometry and selected MBNL1, which has been reported regulated the expression of VM-related proteins MMP2 and LAMC2 through the TGF-β pathway thereby regulating VM formation. MBNL1 is an RBPs regulating RNA alternative splicing, localization, and integrity. Previous studies have found that MBNL1 was associated with ankylosing muscular dystrophy [50], but recently it has been found to play an important role in tumors. For example, LncRNA PVT1 sponged miR-1301-3p to promote MBNL1 expression could regulate laryngeal squamous cell carcinoma progression [51]; MBNL1 inhibited the metastasis of cutaneous squamous cell carcinoma through the TIAL1/MYOD1/Caspase-3 signaling pathway [52]; RSF3-MBNL1-Acin1 axis promoted apoptosis of colorectal cancer cells through post-transcriptional regulation [53]; MBNL1 inhibited colorectal cancer metastasis by degrading Snail mRNA through the Snail/E-cadherin axis [54]; MBNL1 regulated the resistance of HeLa cells to cisplatin through Nrf2 [55]. The study revealed that MBNL1 was downregulated in GBM tissues and cells, and functioned as tumor suppressor. The IPTMnet database and iGPS database predicted that CDK12 phosphorylating MBNL1 at T6 site. Phosphorylation, as one of the most common protein modifications, regulates cell proliferation, differentiation, and signal transduction [56], and plays an important regulatory role in glioma. SMURF2 Thr249 phosphorylation regulated GBM aggressiveness through TGFBR-SMAD-SOX4 axis [57]. CircGLIS3 promoted invasion and tube formation of glioma through Ezrin T567 phosphorylation [58]. The phosphorylation of ERK Ser-249 and Ser-266 phosphorylation might affect the occurrence of glioma through Aml1/Runx1 [59]. Ser-436 phosphorylation promoted YAP-mediated GBM proliferation, migration, and invasion [60]. NDR1 inhibited GBM progression by phosphorylating YAP [61]. PIKE-A regulated STAT3 phosphorylation-mediated G6PD expression to promote GBM cell proliferation and anti-ROS stress response [62]. Further studies revealed that CDK12 phosphorylated MBNL1 on T6 in vitro and in vivo. In this study, MBNL1-WT significantly increased its stability, rather than MBNL1-mut. This finding suggested that CDK12 phosphorylation may mediate its degradation, which requires further investigation. In addition, we found that p-MBNL1 expression was reduced after the T6 phosphorylation site mutation, indicating that CDK12 reduced MBNL1 by T6 phosphorylation site. It has been reported that phosphorylation can affect protein stability [63]. In conclusion, CDK12 reduced MBNL1’s stability through T6 phosphorylation site and thus downregulated its expression. The results above suggested that CDK12 regulated GBM VM formation by phosphorylating MBNL1.

Matrix metalloproteinases (MMPs) are proteolytic enzymes that degrade basement membranes and extracellular matrix. VM formation of tumor was dependent on extracellular matrix MMPs and laminin 5γ2, which could be cleaved into γ2 and γ2x by activated MMP2. These fragments promoted VM formation in 3D cultures [64]. TGF-β is a peptide growth factor, mainly including 3 subtypes: TGF-β1, TGF-β2, TGF-β3. The TGF-β family was associated with cell activation, loss of intercellular connections, and invasion of the matrix. MBNL1 negatively regulated TGF-β-mediated epithelial-mesenchymal transition in endocardial cells [65]. Knockdown of MBNL1 resulted in TGF-β3 secretion increasing and earlier secretion [66]. TGF-β promoted the expression of MMP2 by increasing its enhancer activity, thereby regulating the migration and invasion of lung cancer [67]. LAMC2 encoded the laminin γ subunit, and research has shown that TGF-β1 might act on the promoter region of LAMC2 in colon cancer cells to promote its expression [68]. The above suggested that MBNL1 regulated the expression of VM-related proteins MMP2 and LAMC2 through the TGF-β pathway, thereby regulating the VM formation of GBM.

Further, nude mice transplantation experiment in vivo found that knockdown of BUD13, CDK12, and overexpression of MBNL1 alone inhibited the growth of transplanted tumors, prolonged the survival time of nude mice, and inhibited the VM in transplanted tumors, and the effect of three coregulation was the most significant. These findings indicated that knockdown of BUD13 and CDK12 combined with overexpression of MBNL1 had potential clinical value.

In conclusion, the study revealed the expression and interaction of BUD13, CDK12, and MBNL1 in GBM tissues and cells for the first time. Knockdown of BUD13, CDK12, and overexpression of MBNL1 significantly inhibited proliferation, migration, invasion, and tube formation of GBM cells. METTL3 enhanced the stability of BUD13 mRNA and upregulated its expression through m6A methylation. BUD13 enhanced the stability of CDK12 mRNA and upregulated its expression. CDK12 phosphorylated MBNL1, thereby regulating the proliferation, migration, invasion, and tube formation of GBM. The research provided not only a theoretical and experimental basis for the malignant progression of GBM, but also a new reference for molecular targeted therapy of GBM.

## Supplementary information

Supplementary Materials and Methods

Supplementary Table

Original western blots

checklist

Figure S1

Figure S2

Figure S3

Figure S4

Figure S5

Figure S6

Supplementary Information

## Data Availability

The data that support the findings of this study are available from the corresponding author upon reasonable request.
