# Supplementary material for: The mechanism of BUD13 m6A methylation mediated MBNL1-phosphorylation by CDK12 regulating the vasculogenic mimicry in glioblastoma cells
Source: Cell Death Dis. 2022 Dec 3;13(12):1017. doi: 10.1038/s41419-022-05426-z (PMC9719550; doi:10.1038/s41419-022-05426-z)
Supplement: Supplementary file 1 — Supplementary Materials and Methods [file 41419_2022_5426_MOESM1_ESM.docx]

**Supplemental Materials and Methods**

**Cell Culture**

U251 and U373 tumor cells were cultured in Dulbecco’s modified Eagle’s medium (DMEM, HyClone, UT, USA) supplemented with 10% fetal bovine serum (Gibco, NY, USA). NHAs were cultured in RPMI-1640 medium (Gibco, NY, USA) with 10% FBS. All cells were maintained in a humidified incubator at 37°C with 5% CO_2_.

**RNA extraction and quantitative real**-**time PCR**

Total RNA was separated from cells and tissues using Trizol reagent (Life Technologies, CA, USA). GAPDH was used as an endogenous control. RNA concentration and quality were determined by Nanodrop Spectrophotometer (ND-100, Thermo Fisher Scientific, MA, USA). All qRT-PCR reactions were conducted by 7500 Fast System (ABI, Shanghai, China) using One-Step SYBR PrimeScript RT-PCR Kit (Takara, Kyoto, Japan). Relative expression values were calculated using the relative quantification (2^-ΔΔCt^) method.

**Cell transfection**

The short-hairpin RNA against BUD13 (BUD13(-)) and its nontargeting sequence (BUD13(-)NC); the short-hairpin RNA against METTL3 (METTL3(-)) and its nontargeting sequence (METTL3(-)NC); the short-hairpin RNA against CDK12 (CDK12(-)), CDK12 full length (CDK12(+)) sequence and their respective negative control (CDK12(-)NC or CDK12(+)NC) plasmids; the short-hairpin RNA against MBNL1 (MBNL1(-)), MBNL1 full length (MBNL1(+)) sequence and their respective negative control (MBNL1(-)NC or MBNL1(+)NC) plasmids were synthesized by Gene-Pharama (Gene-Pharama, Shanghai, China). The BUD13 1645 methylation site mutation plasmid (BUD13-m6A-mut) and its wild type (BUD13-m6A-WT), MBNL1 T6 phosphorylation site mutaion plasmid (MBNL1-mut) and its wild type (MBNL1-WT) were synthesized by Gene-Pharama (Gene-Pharama, Shanghai, China). Cells were seeded in a 24-well plate and transfected with plasmids when cells were cultured to 70-80% confluence using Lipofectamine 3000 reagent (Life Technologies, CA, USA) following the manufacture’s protocol. G418, puromycin, and bleomycin (Sigma-Aldrich, MO, USA) were used to select the stably transfected cells. Resistant cell clones were established around approximately 4 weeks.

**Western blot**

Total protein was lysed with RIPA buffer and protease inhibitors (Beyotime Institute of Biotechnology, Jiangsu, China). The protein concentration was analyzed by BCA protein assay (Beyotime Institute of Biotechnology, Jiangsu, China). An equal amount of protein sample was subjected to SDS-PAGE gels and transferred to PVDF membranes (Millipore, MA, USA). Membranes were blocked with 5% nonfat milk for 2 h at room temperature and then incubated with primary antibodies overnight at 4°C as follows: BUD13 (1:1000; Proteintech, IL, USA), METTL3 (1:5000; Proteintech, IL, USA), CDK12 (1:1000; Cell Signaling Technology, MA, USA), MBNL1 (1:2000; Huada, Beijing, China), GST tag (1:10000; Proteintech, IL, USA), and FLAG tag (1:5000; Proteintech, IL, USA), p-MBNL1 (1:1000; Huada, Beijing, China), MMP2 (1:1000; Proteintech, IL, USA), LAMC2 (1:1000; Proteintech, IL, USA), GAPDH (1:10000; Proteintech, IL, USA), After washing 3 times with TTBS, the membranes were incubated with respective appropriate secondary antibodies at room temperature for 2h as follows: goat anti-mouse (1:10000; Proteintech, IL, USA) or goat anti-rabbit (1:10000; Proteintech, IL, USA). The blots were visualized with Enhanced Chemiluminescence (ECL) Kit (Beyotime Institute of Biotechnology, Jiangsu, China) and scanned by ChemImager 5500 V2.03 software. The relative integrated density values (IDV) were calculated using Image-J software based on GAPDH as endogenous control.

**Cell viability assay**

Cell Counting Kit-8 (CCK8) assay (Beyotime Institute of Biotechnology, Jiangsu, China) was performed to determine the viability of GBM cells. 2×10^3^ cells were seeded in 96-well plates. Each group had 3 replicates. After 48 h incubation, 10 µL CCK8 solution was added to each well and incubated with cells for 2 h. Absorbance was measured at a wavelength of 450 nm using a SpectraMax M5 microplate reader.

**Cell migration assay**

Cell migration assay was performed using the HoloMonitor M4 culture system (Phase Holographic Imaging PHI AB, Lund, Sweden) according to the manufacturer’s protocols. The cells of each group were inoculated into a six-well plate at a concentration of 2×10^4^ cells/mL. After the cells were attached to the culture plate, they were placed on the HoloMonitor M4 culture system and set for imaging for 6 h at 1 h intervals. For each experimental group, we showed the last image frame and cell movements. At the start of the analysis, 5 visually identifiable cells in each experimental set were selected for tracking. Their movements were displayed in spatial X-Y plots.

**Cell invasion assay**

The cell invasion was assessed by transwell assay using an 8 μm pore size polycarbonate membrane (Corning, NJ, USA). The cells were resuspended in serum-free medium at a density of 2×10^5^ cells/mL and seeded into the upper chamber of polycarbonate membrane (pre-coated with Matrigel and incubated at 37°C for 30 min before the invasion assay started). After incubation at 37°C for 36 h, the cells invaded from the upper chamber to the lower surface of the membrane and the cells were fixed with methanol and glacial acetic acid at a ratio of 3:1 before staining with 20% Giemsa. Five random fields were chosen to count and take photos under a microscope.

**Three**-**dimensional tube formation assay**Each hole in the 96-well culture plate was covered with 100 μL Matrigel (BD, NJ, USA). The 96-well culture plate was incubated for 30 min at 37°C. Then, the cells were resuspended in 100 μL serum-free medium and seeded onto the surface of Matrigel at a density of 6×10^5^ cells/mL and incubated for 8 hours. The cells vascular structures were observed and photographed under an inverted microscope (Olympus, Tokyo, Japan). An independent observer counted the total number of tube-like structures per image.

**RNA immunoprecipitation (RIP) assay**

RIP assay was performed using the Magna RNA-Binding Protein Immunoprecipitation Kit (Millipore, MA, USA) according to the manufacture’s protocol. Whole cell lysate, magnetic beads containing METTL3 antibody (Proteintech, IL, USA), BUD13 antibody (Proteintech, IL, USA), or IgG were incubated with RIP buffer. After binding to proteinase K, the co-immunoprecipitated RNA was isolated. The RNA concentration was measured by Nanodrop Spectrophotometer (ND-100, Thermo Fisher Scientific, MA, USA), and PCR was performed after purification to analyze the RNA binding to the target protein.

**RNA pull**-**down assay**

RNA pull-down assay was performed according to the instructions of the RNA-Protein Pull-Down Kit (Thermo Fisher Scientific, MA, USA). Biotin-labeled BUD13, CDK12 and its antisense RNA, whole cell lysate, and magnetic beads were incubated together. The bead-RNA-protein complex was collected by low-speed centrifugation, and washed with spin columns, boiled in SDS buffer. Then the protein bound to the target RNA was detected by western blot.

**Nascent RNA capture assay**

Nascent RNA was detected by Click-iT Nascent RNA Capture Kit (Thermo Fisher Scientific, MA, USA) following the manufacture’s protocols. We used 5-ethynyl uridine to label the nascent RNA, which was then isolated using streptavidin magnetic beads. Finally, Nascent RNA was analyzed by qPCR.

**CD34**-**PAS staining**

CD34-PAS was examined for the existence of VM. Tumor xenograft tissue samples were fixed with 4% formaldehyde, embedded in paraffin, and sectioned into 5 μm tumor slides. These were deparaffinized in xylene, hydrated, and boiled in EDTA antigen-unmasking solution. When cooled to room temperature, slides were incubated in peroxide at room temperature for endogenous peroxidase ablation, blocked with goat serum, and stained with CD34 antibody (1:50; Proteintech, IL, USA) overnight at 4°C. After washing three times with PBS and incubating with goat-anti-mouse secondary antibody at room temperature for 10 min, the slides were treated with a DAB Kit (MaiXin Biotech, Fuzhou, China). Then, the slides were exposed to the periodic acid solution for 10 min, incubated with Schiff solution for 10 min in the dark, and counterstained with Mayer’s hematoxylin (Baso, Guangdong, China). Lastly, the slides were viewed under a light microscope to detect CD34 and PAS signals and counted the pipe structure of CD34(-)+PAS(+). Immunohistochemical staining was performed with the help of UltraSensitive S-P Kit (MaiXin Biotech, Fuzhou, China).

**Tumor xenograft in nude mice**

For in vivo study, Four-week-old athymic nude mice (BALB/c) were purchased from Beijing Vital River Laboratory Animal Technology. All the animal experiments were performed following the Animal Welfare Act and approved by the Ethics Committee of China Medical University. The nude mice were divided into five groups: Control group, BUD13(-) group, CDK12(-) group, MBNL1(+) group, and BUD13(-)+CDK12(-)+MBNL1(+) group. For subcutaneous implantation, the stably transfected and expressed cells were selected, and 3×10^5^ cells were injected subcutaneously under the right axilla area. The volumes of the tumor were measured every 5 days according to the formula: mm^3^=length×width^2^/2. At 45 days after subcutaneous injection, the mice were sacrificed and the tumors were separated. As for the survival study, 3×10^5^ cells were injected into the right striatum. The number of survival mice was recorded every day and survival analysis was performed according to the Kaplan-Meier survival curve.
