# Supplementary material for: The mechanism of BUD13 m6A methylation mediated MBNL1-phosphorylation by CDK12 regulating the vasculogenic mimicry in glioblastoma cells
Source: Cell Death Dis. 2022 Dec 3;13(12):1017. doi: 10.1038/s41419-022-05426-z (PMC9719550; doi:10.1038/s41419-022-05426-z)
Supplement: Supplementary file 2 — Supplementary Table [file 41419_2022_5426_MOESM2_ESM.docx]

Table S1 Primers used for qRT-PCR

| **Gene** | **Sequence (5’**-**3’)** |
| --- | --- |
| BUD13 | F: TGCGGATTGTGGATGATGATGTGAG |
|  | R: GCCTCCATCTGCTTTACCTCTTCTG |
| METTL3 | F: CTGTGTCCATCTGTCTTGCCATCTC |
|  | R: ACCTCGCTTTACCTCAATCAACTCC |
| CDK12 | F: TCGAAGCACAAGCGGCATAAGTC |
|  | R: TCGGAGAAGGTGTCGGAATCAGAG |
| MBNL1 | F: AAGAGGAAAGGACAGTGTGCTTGG |
|  | R:GTGGATGTCTTGTGGCTGAGGAAC |
| GAPDH | F: CAGGAGGCATTGCTGATGAT |
|  | R: GAAGGCTGGGGCTCATTT |

Table S2 The short hairpin RNAs and their sequences

| **Gene** | **Sequence (5’**-**3’)** |
| --- | --- |
| sh-NC | TTCTCCGAACGTGTCACGT |
| sh-METTL3 | GCACATCCTACTCTTGTAACC |
| sh-BUD13 | GCTCCTAATGTCACTTATTCC |
| sh-CDK12 | GCAGTCGTCATTCCAGTATCT |
| sh-MBNL1 | GCACAATGATTGACACCAATG |

Table S3 The sites mutation

| **Site** | **Mutation** |
| --- | --- |
| BUD13 m6A methylation site at 1645 | A→C |
| MBNL1 T6 phosphorylation site | Thr (ACA)→Ala (GCA) |
