# Supplementary material for: The mechanism of BUD13 m6A methylation mediated MBNL1-phosphorylation by CDK12 regulating the vasculogenic mimicry in glioblastoma cells
Source: Cell Death Dis. 2022 Dec 3;13(12):1017. doi: 10.1038/s41419-022-05426-z (PMC9719550; doi:10.1038/s41419-022-05426-z)

Original western blots of "The mechanism of BUD13 m6A methylation mediated MBNL1-phosphorylation by CDK12 regulating the vasculogenic mimicry in glioblastoma cells"

Figure 1A representative image

BUD13

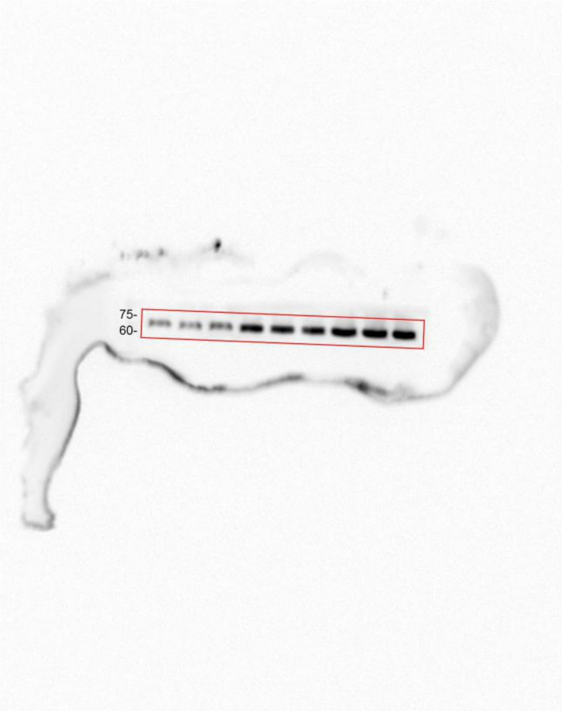

GAPDH

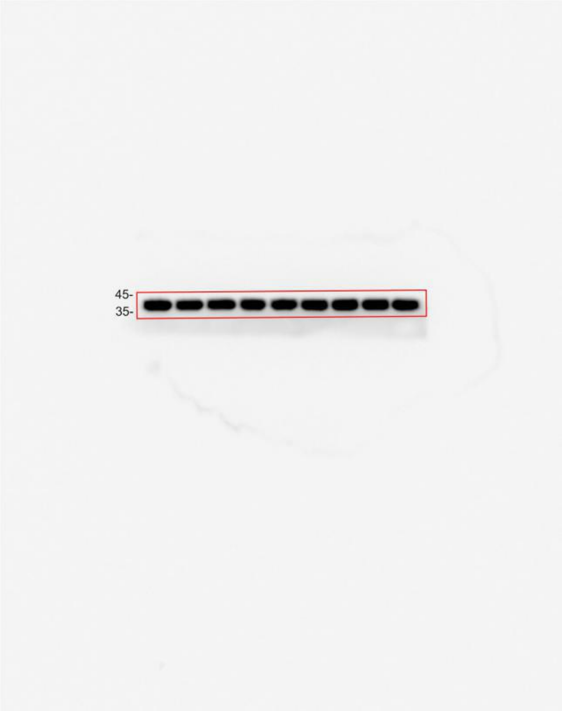

Figure 1B representative image

BUD13

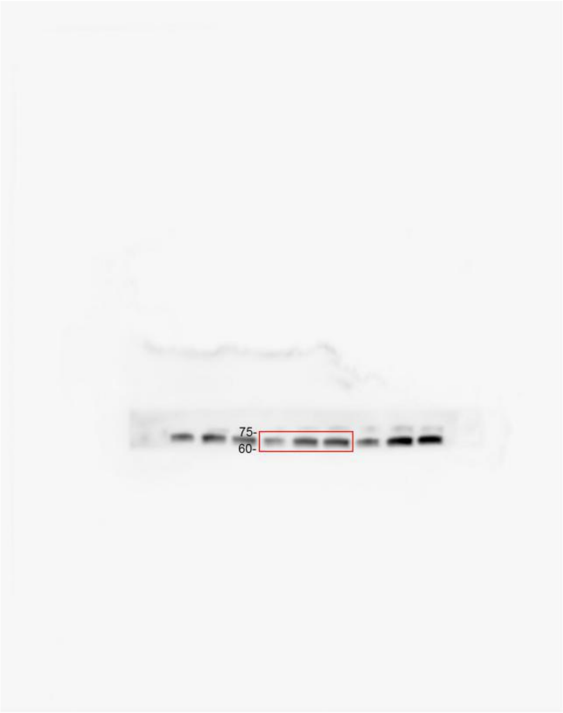

GAPDH

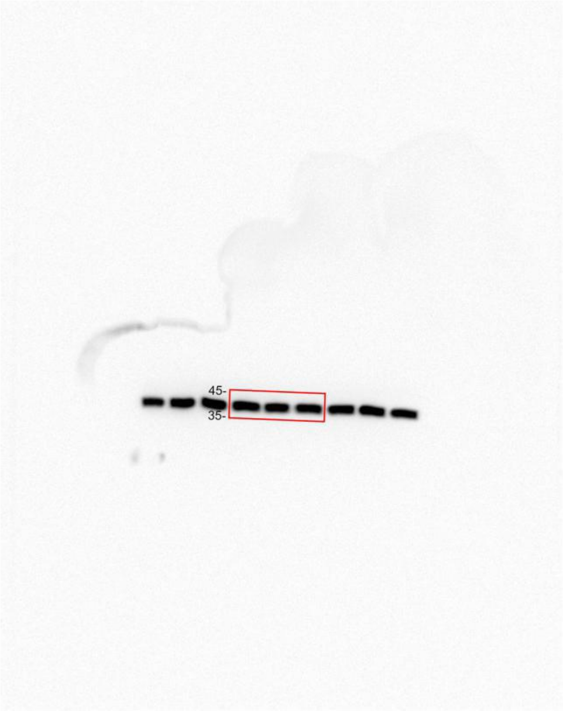

Figure 1G representative image

U251 MMP2

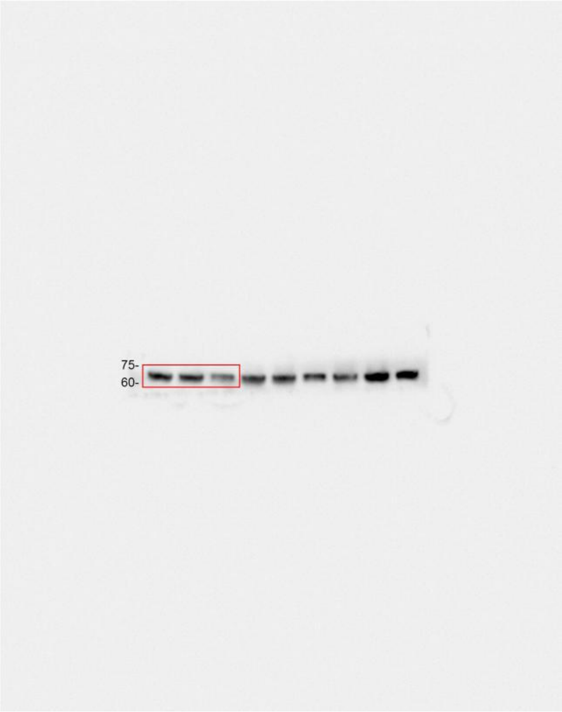

U251 LAMC2

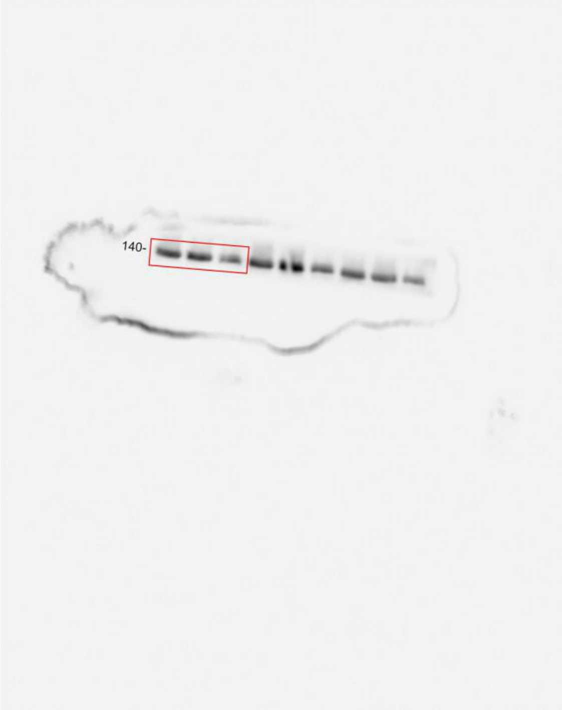

U251 GAPDH

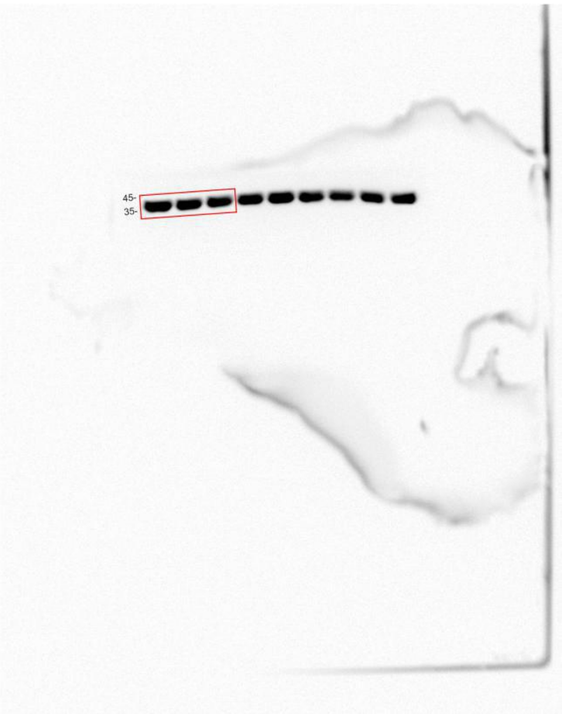

U373 MMP2

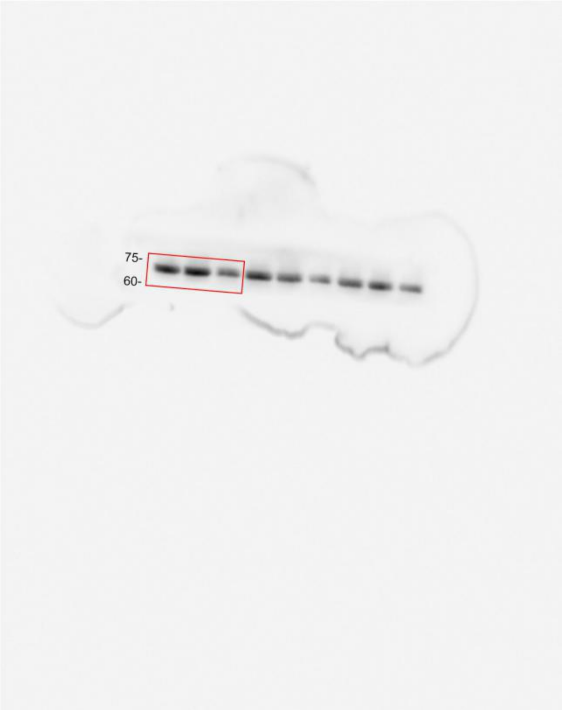

U373 LAMC2

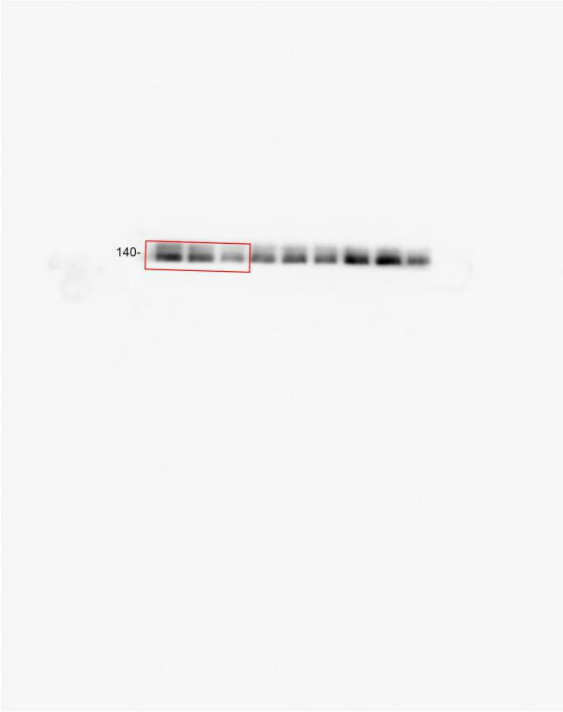

U373 GAPDH

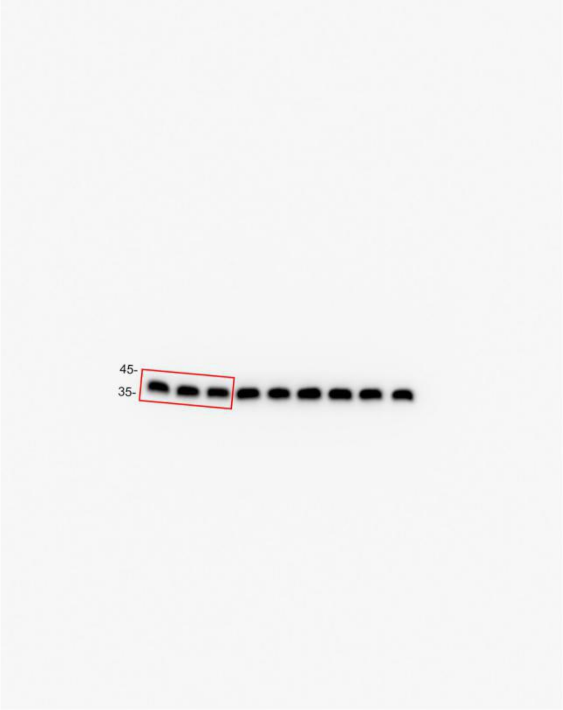

Figure 2E representative image

U251 MMP2

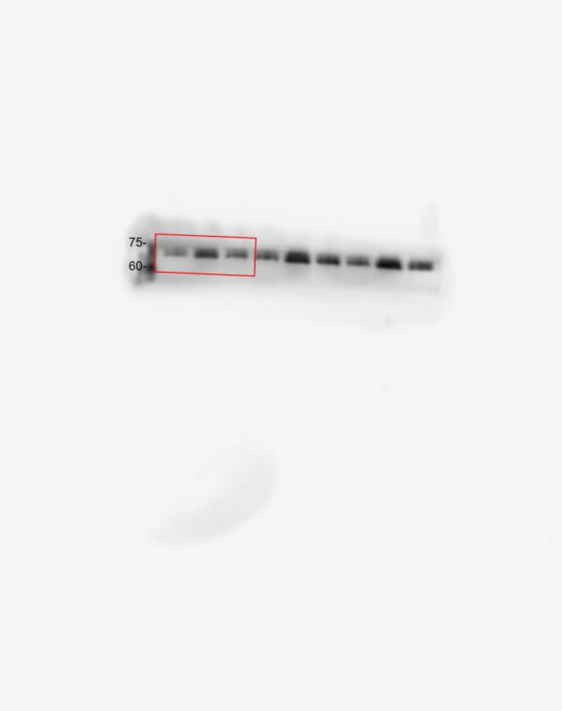

U251 LAMC2

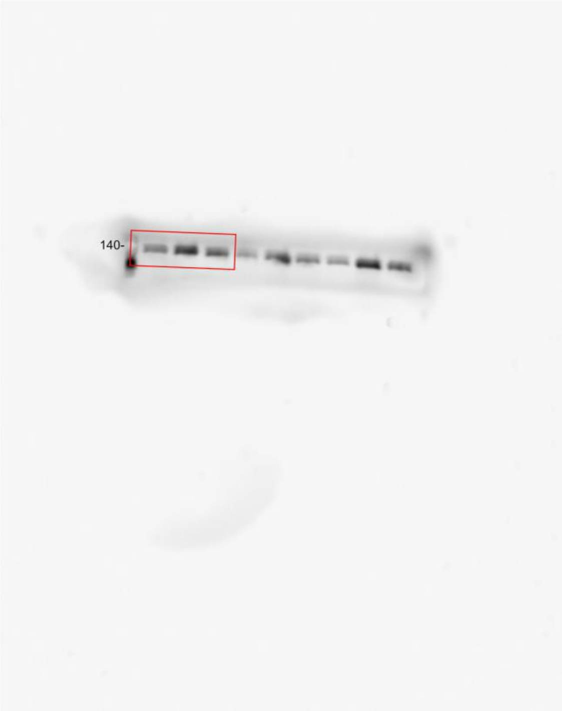

U251 GAPDH

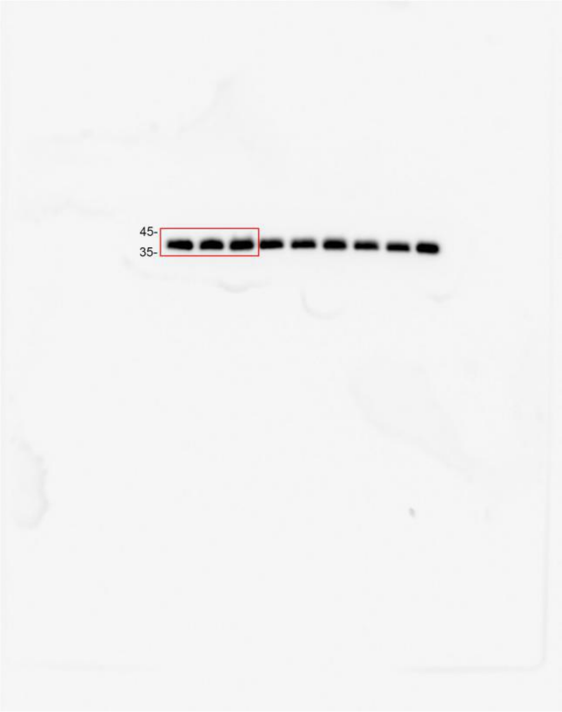

U373 MMP2

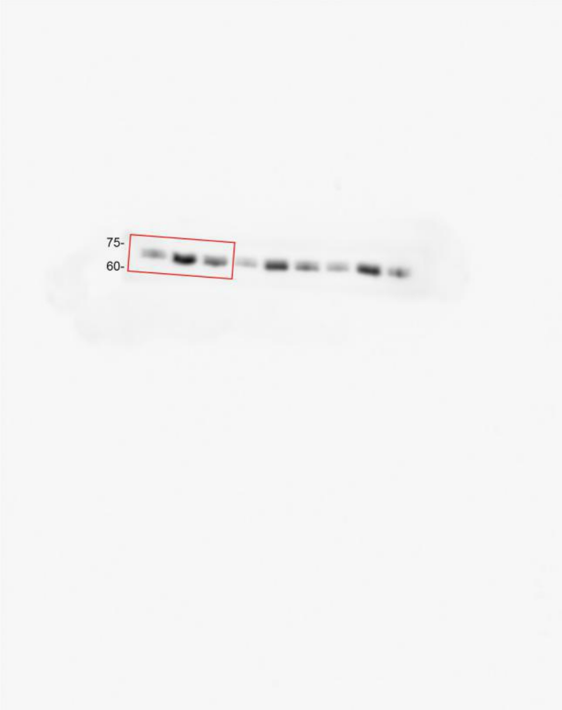

U373 LAMC2

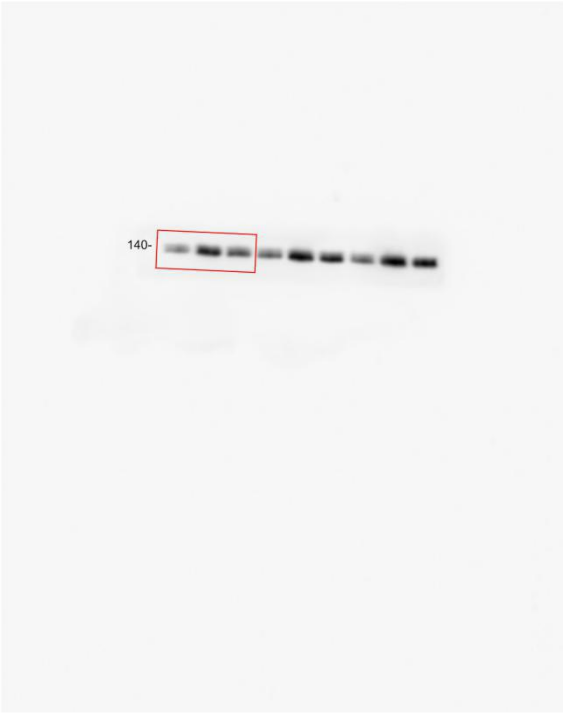

U373 GAPDH

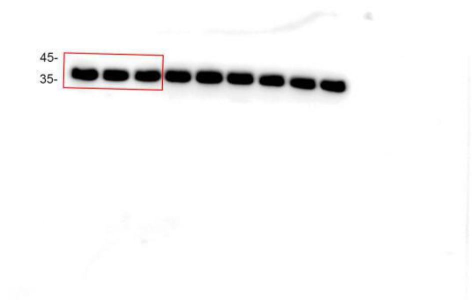

Figure 3A representative image

CDK12

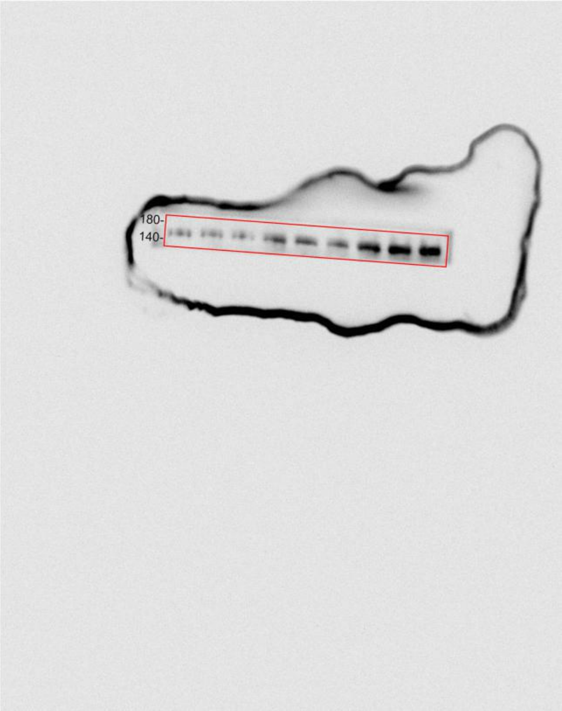

GAPDH

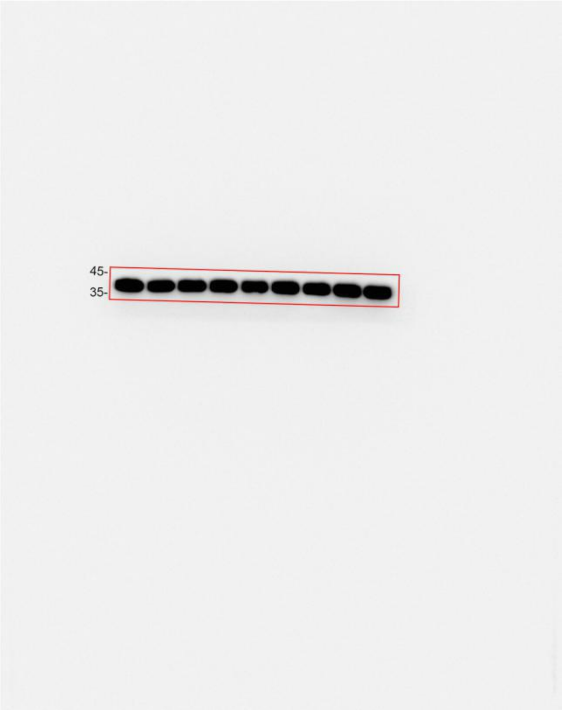

Figure 3B representative image

CDK12

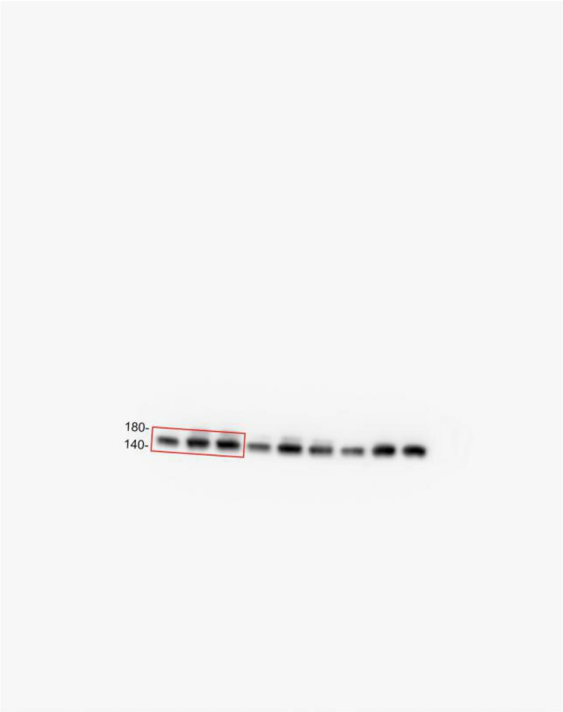

GAPDH

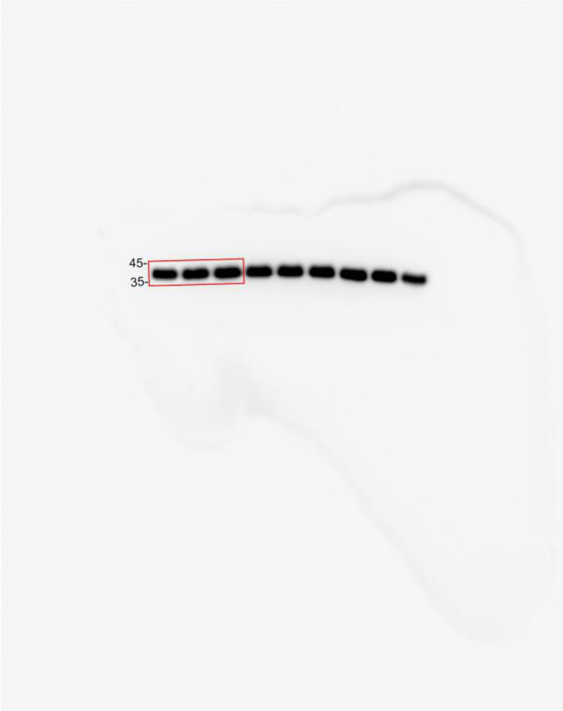

Figure 3G representative image

U251 MMP2

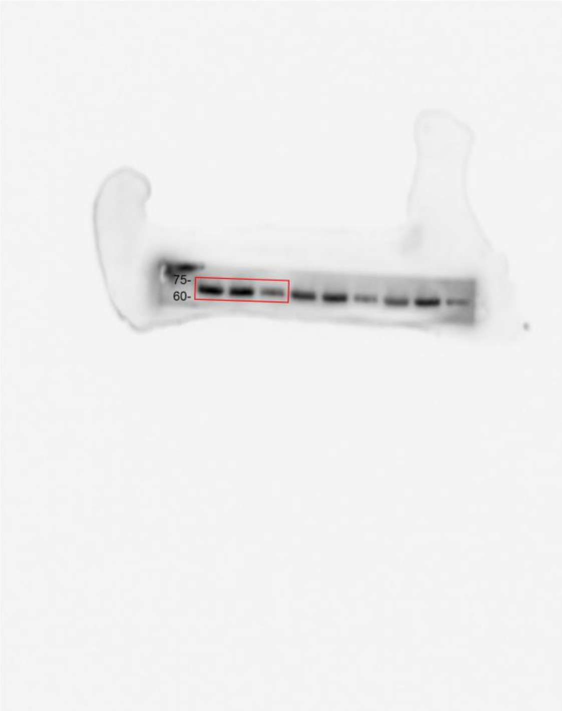

U251 LAMC2

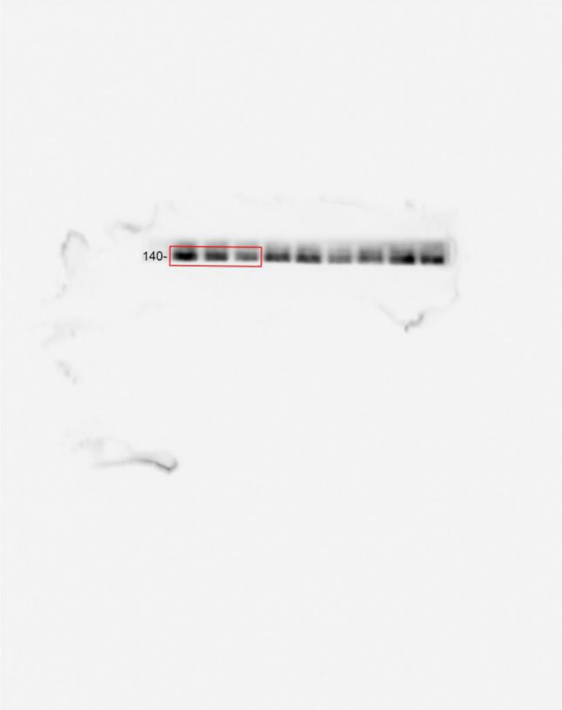

U251 GAPDH

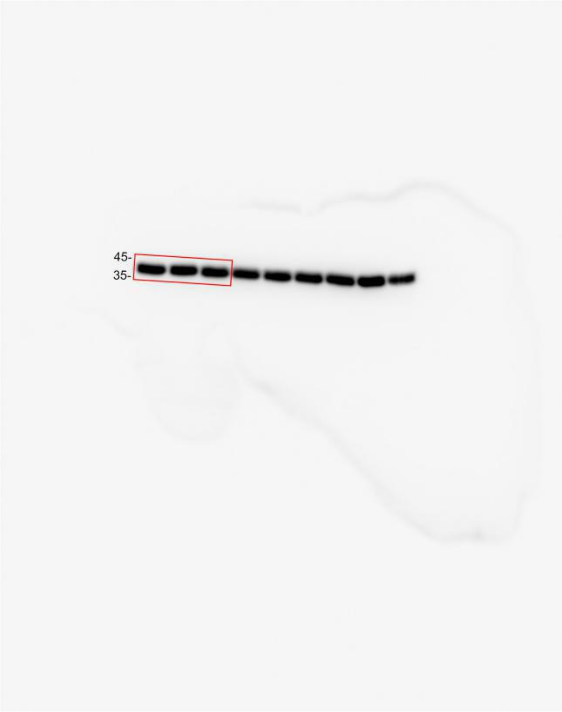

U373 MMP2

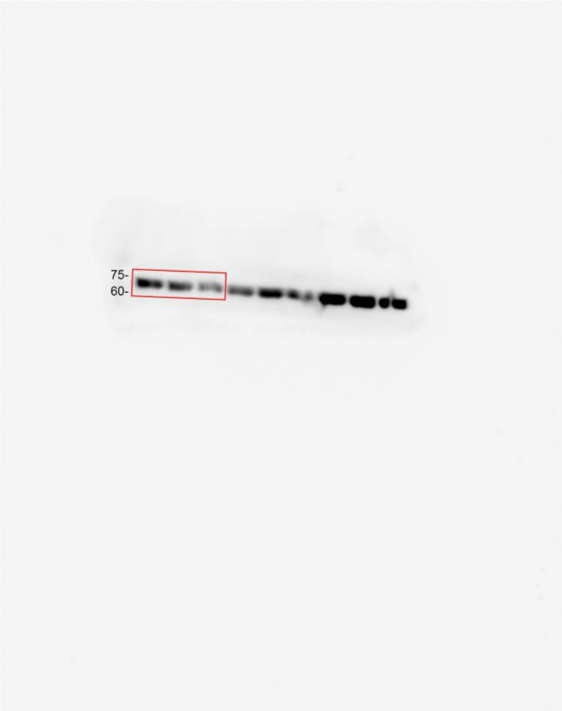

U373 LAMC2

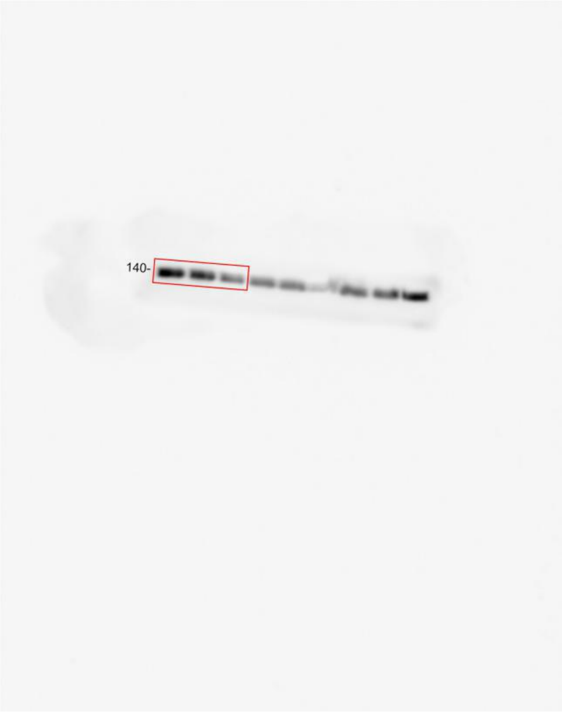

U373 GAPDH

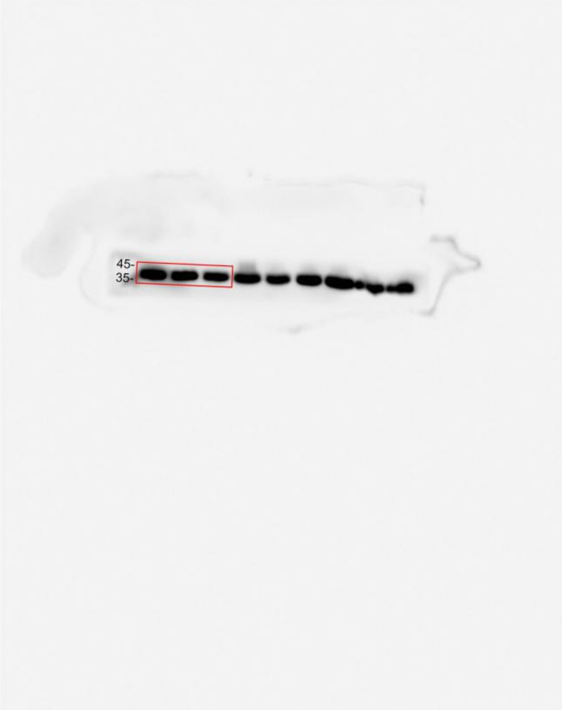

Figure 4A representative image

MBNL1

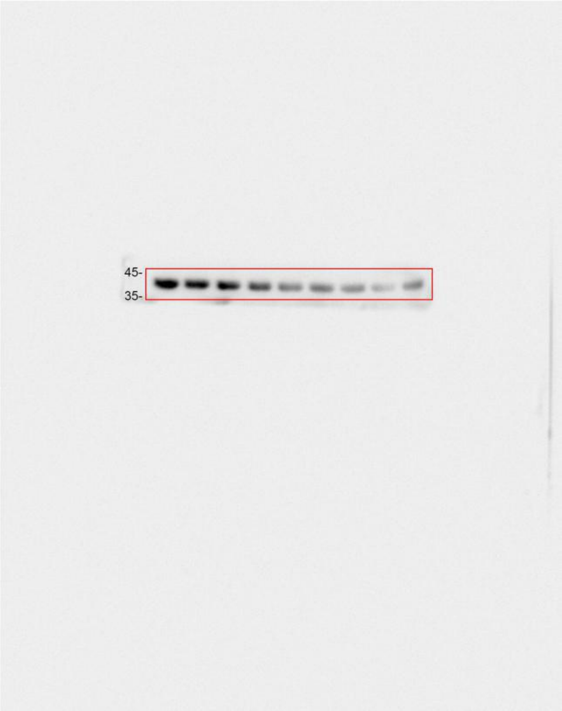

GAPDH

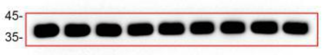

Figure 4B representative image

MBNL1

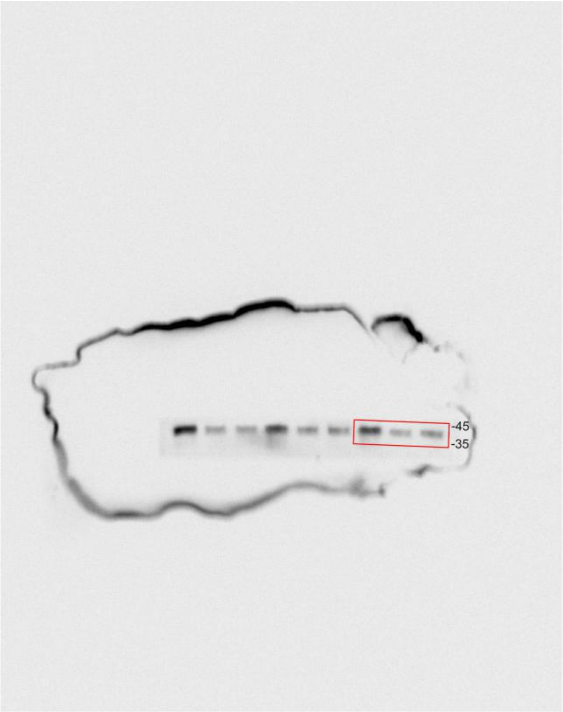

GAPDH

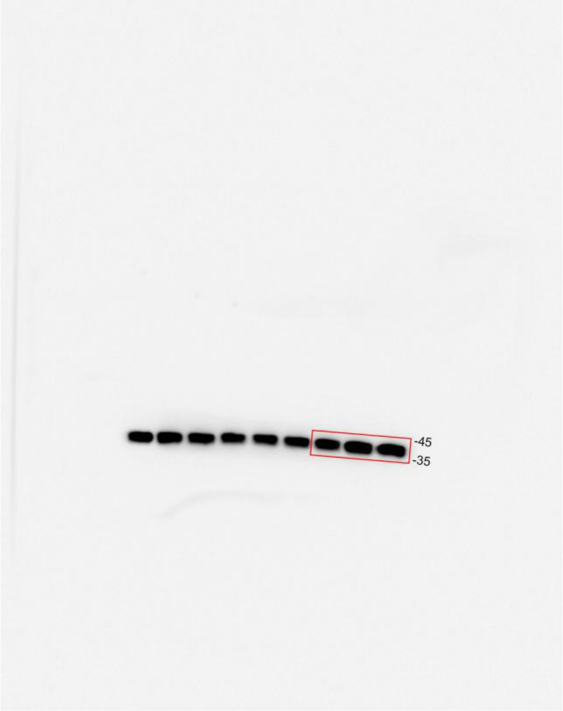

Figure 4G representative image

U251 MMP2

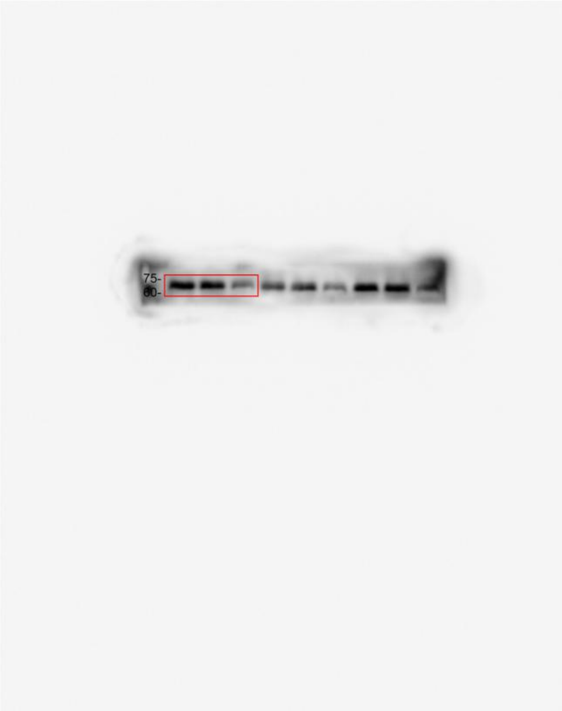

U251 LAMC2

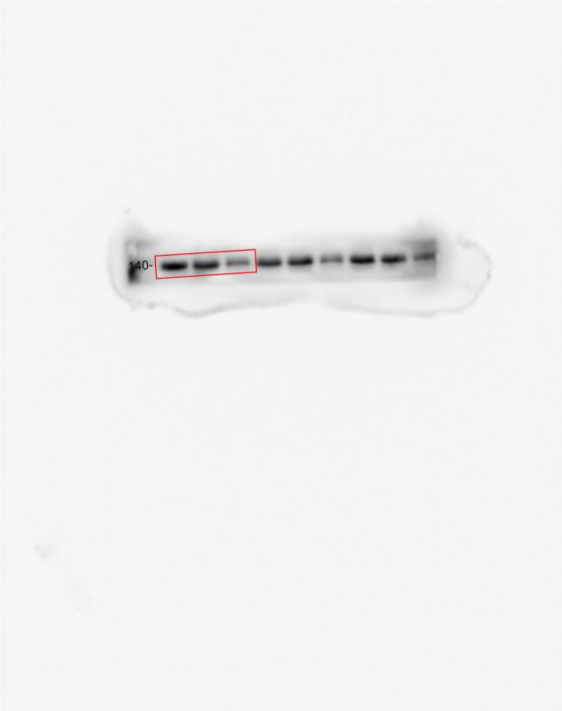

U251 GAPDH

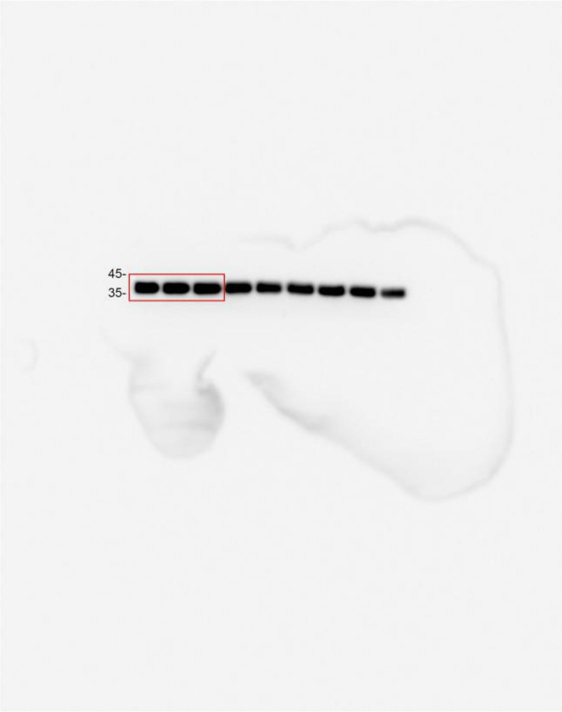

U373 MMP2

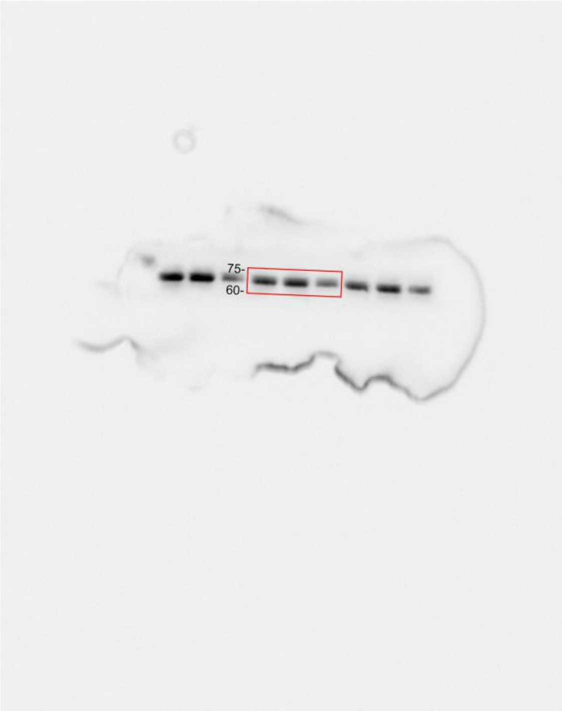

U373 LAMC2

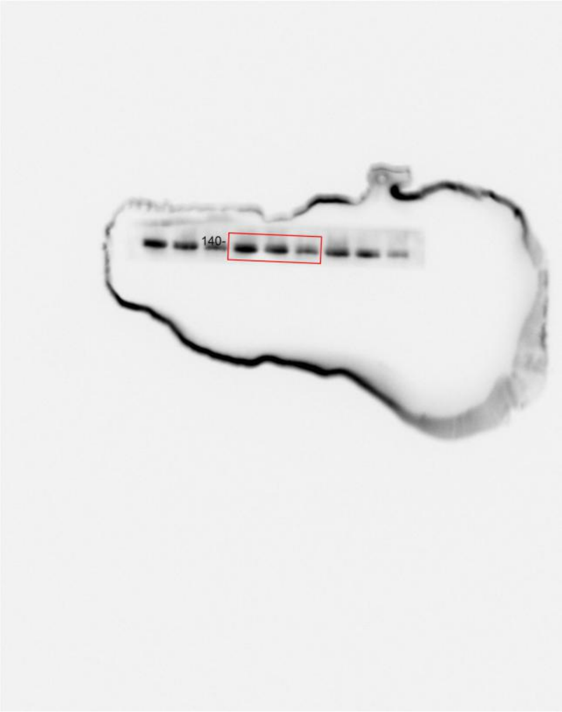

U373 GAPDH

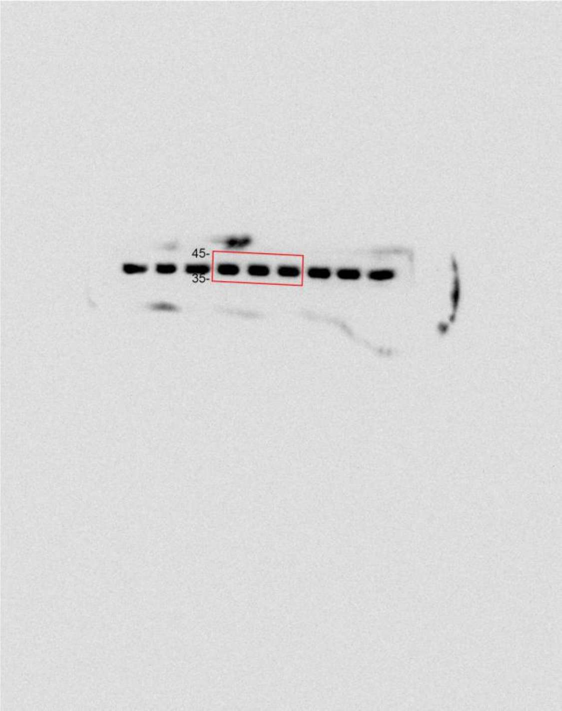

Figure 5B representative image

MBNL1

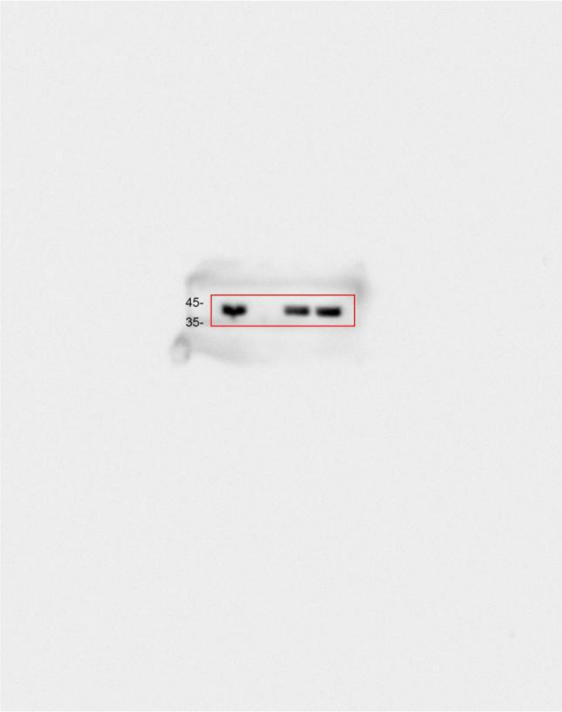

CDK12

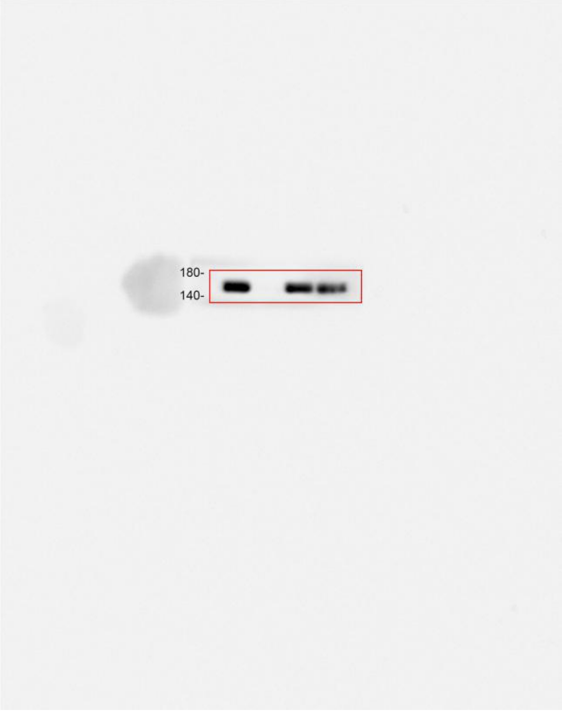

Figure 5C representative image

IP: GST IB: FLAG

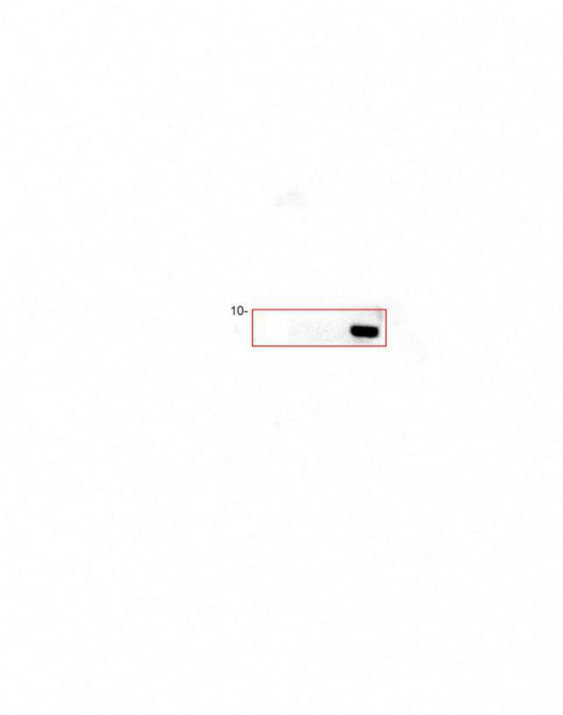

IP: GST IB: GST

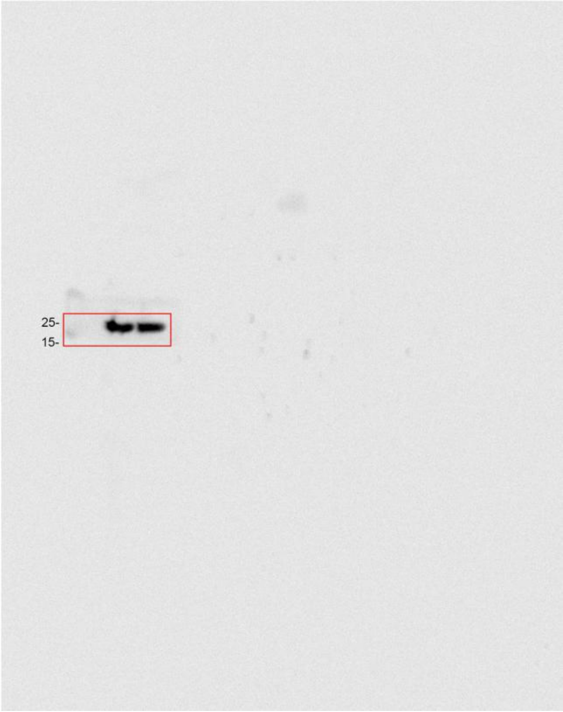

IP: FLAG IB: FLAG

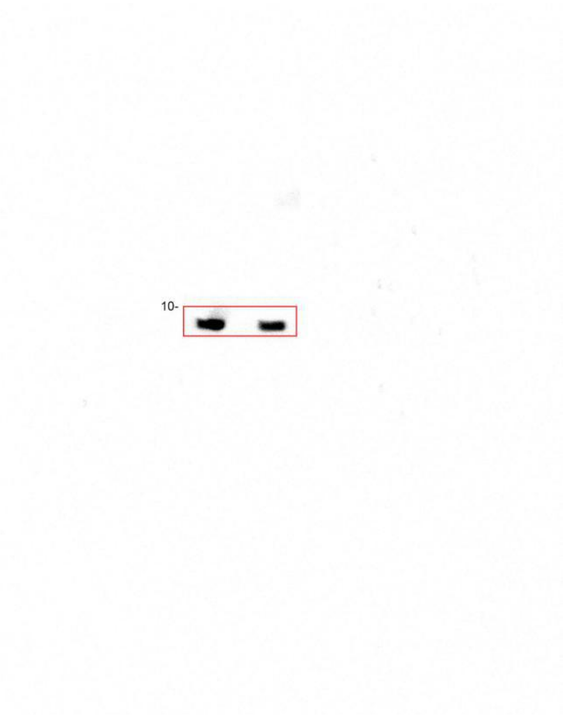

IP: FLAG IB: GST

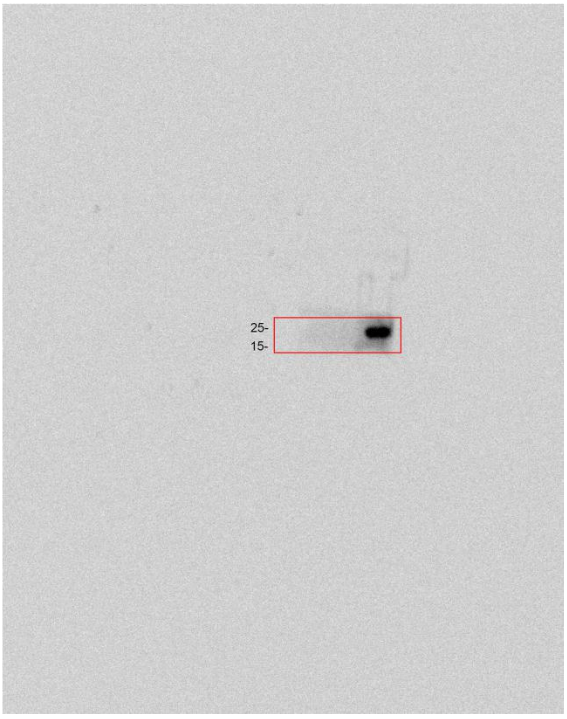

IP: Input IB: FLAG

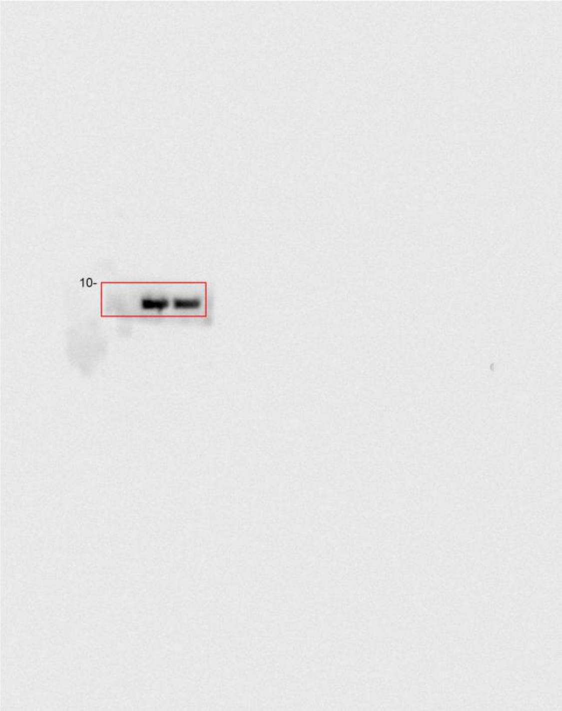

IP: Input IB: GST

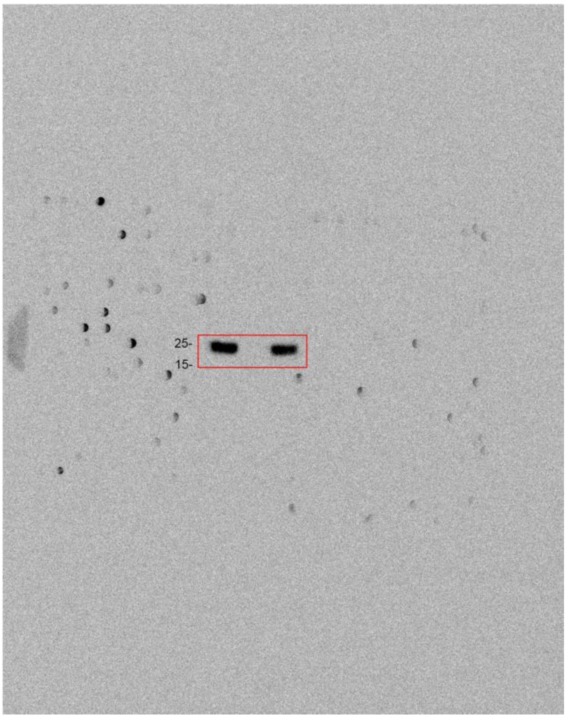

Figure 5D representative image

FLAG

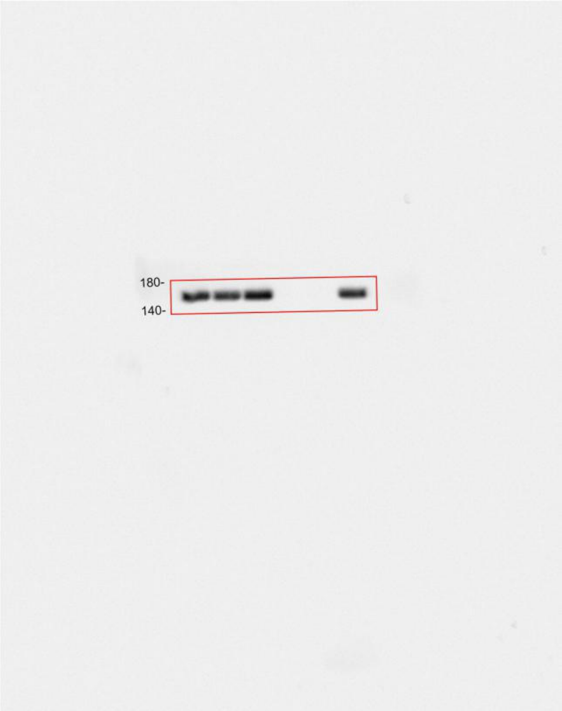

GST

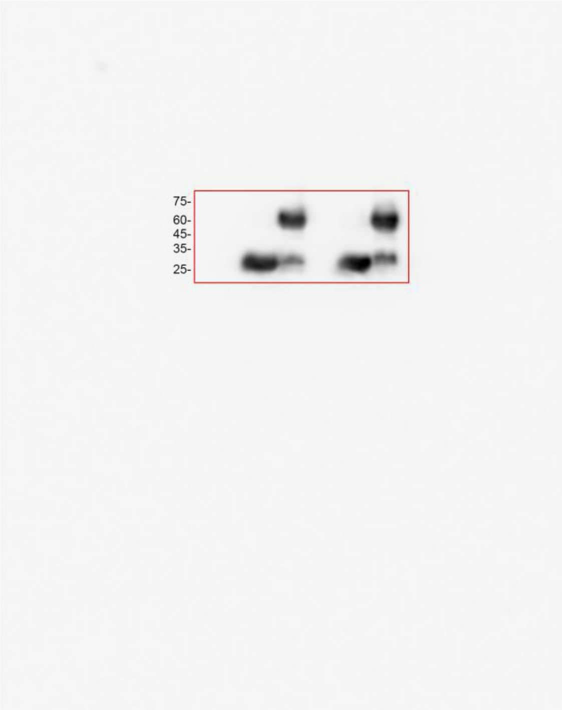

Figure 5F representative image

U251 Control MBNL1

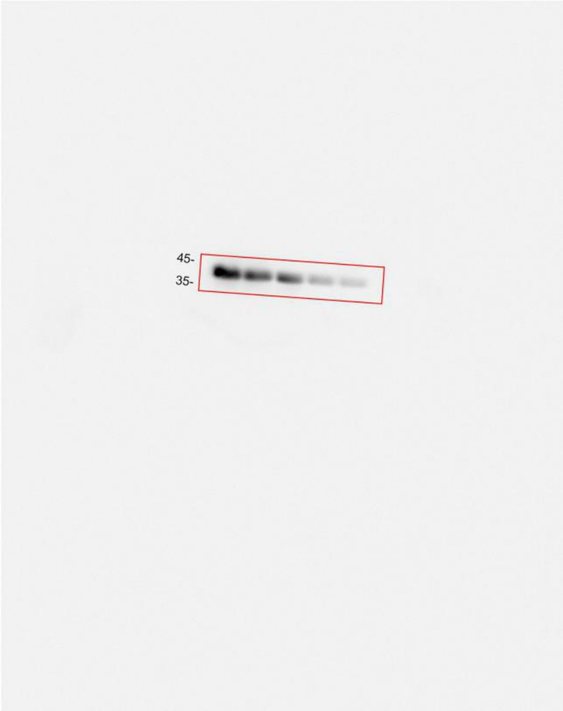

U251 Control GAPDH

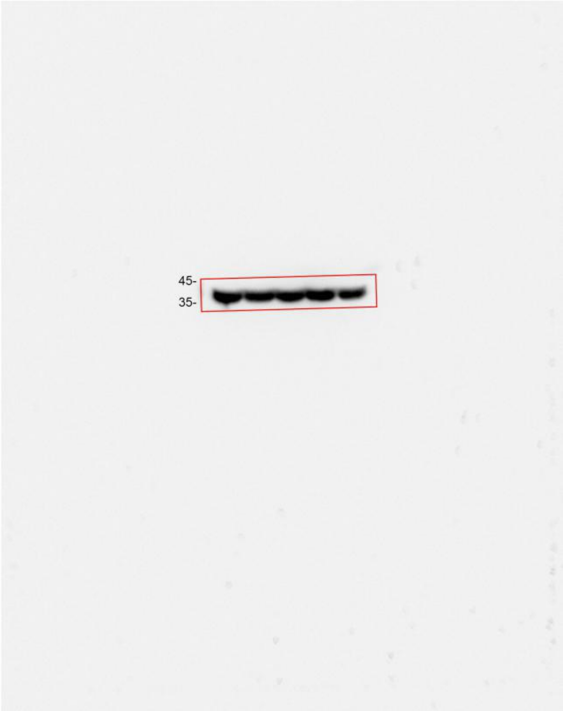

U251 MBNL1-WT MBNL1

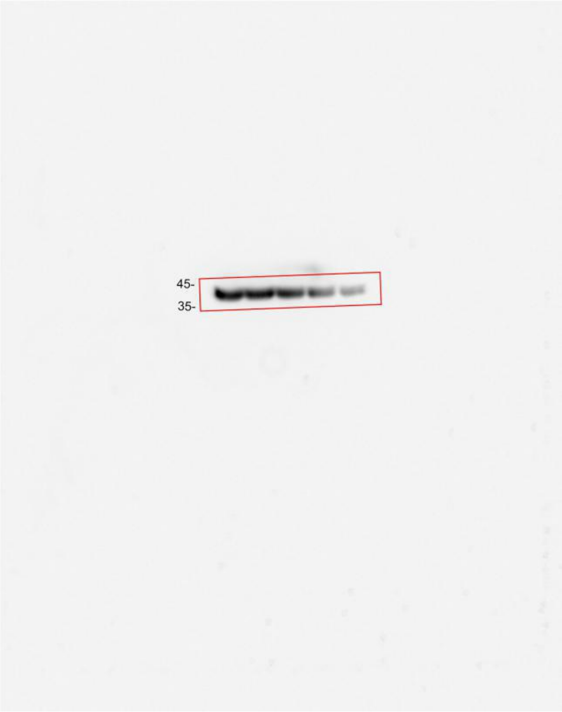

U251 MBNL1-WT GAPDH

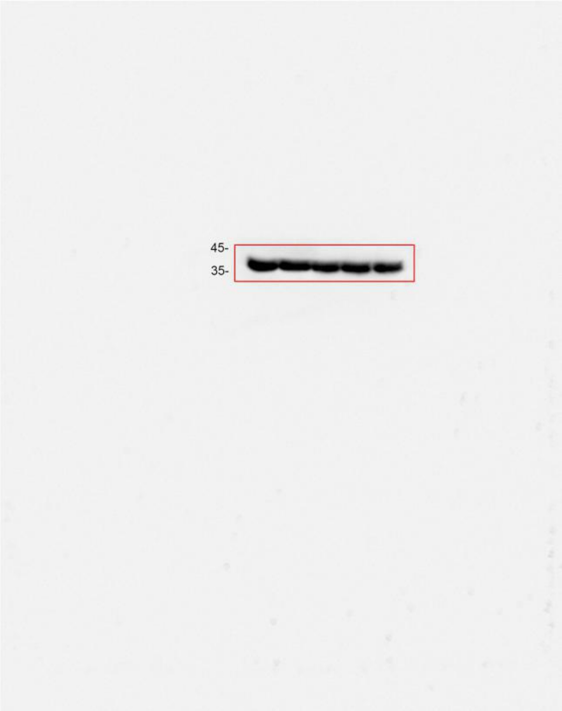

U251 MBNL1-mut MBNL1

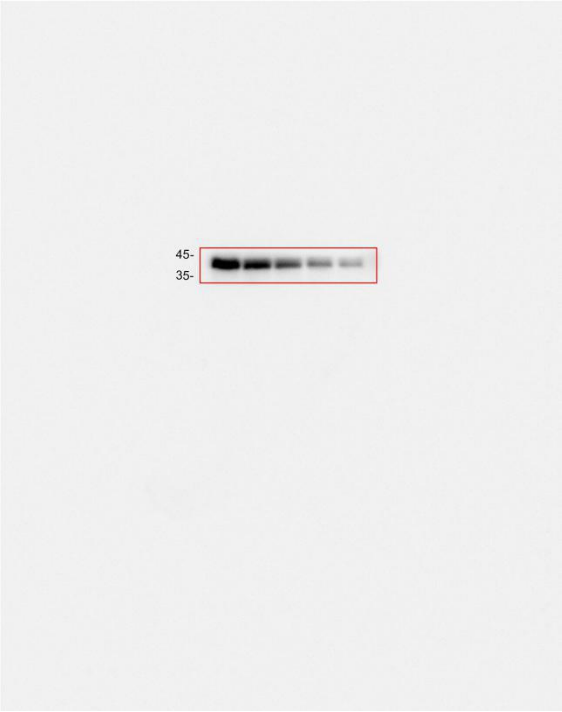

U251 MBNL1-mut GAPDH

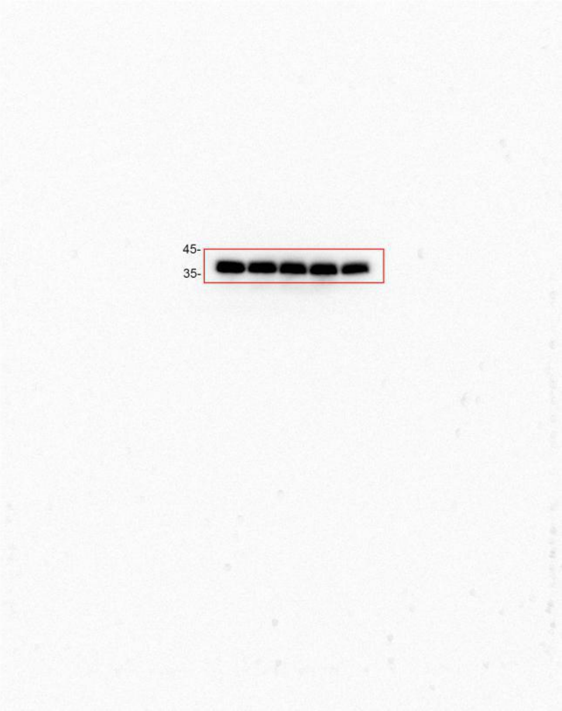

U373 Control MBNL1

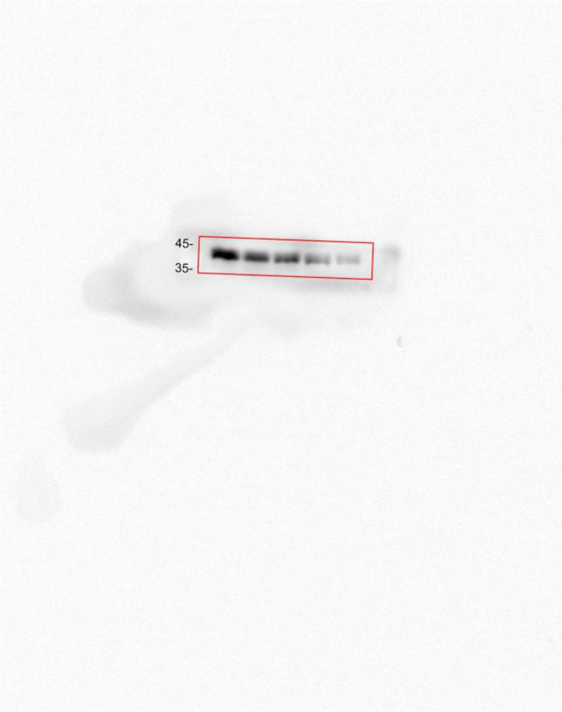

U373 Control GAPDH

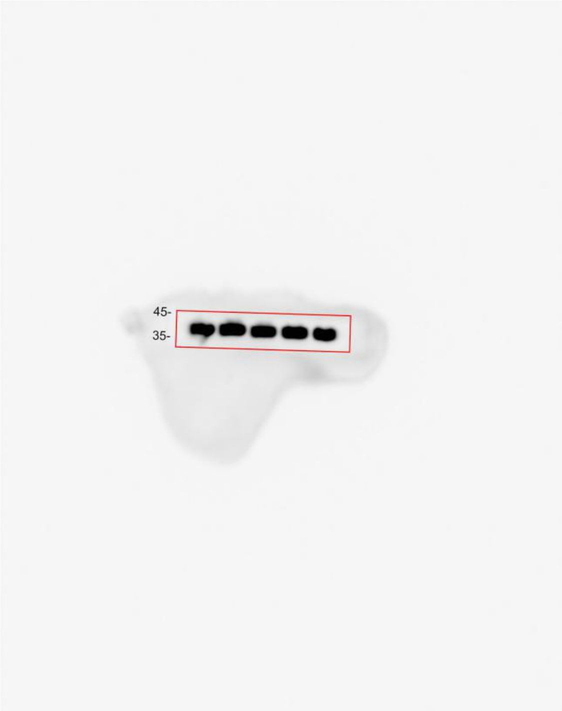

U373 MBNL1-WT MBNL1

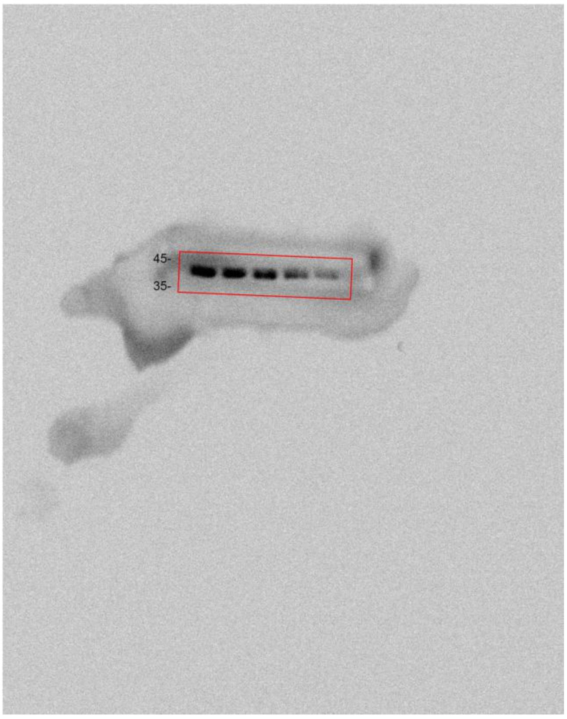

U373 MBNL1-WT GAPDH

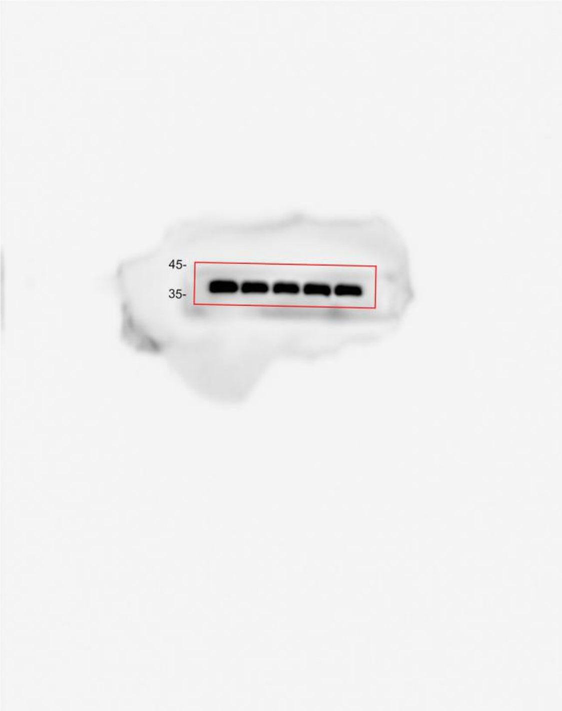

U373 MBNL1-mut MBNL1

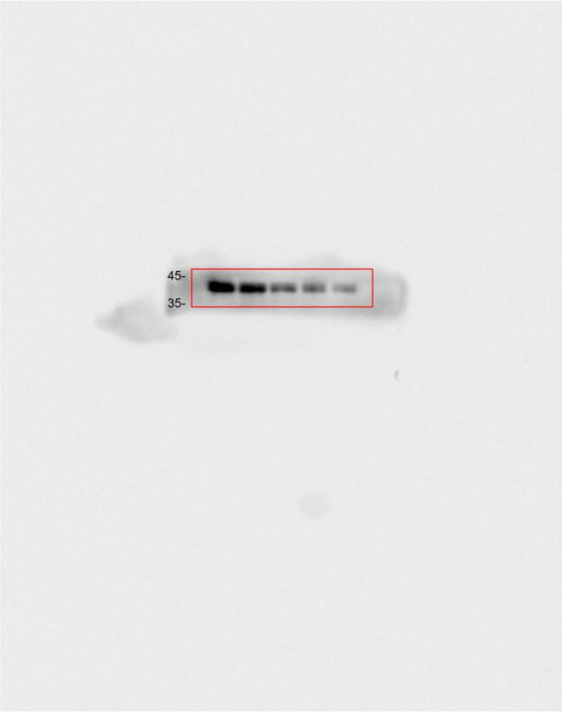

U373 MBNL1-mut GAPDH

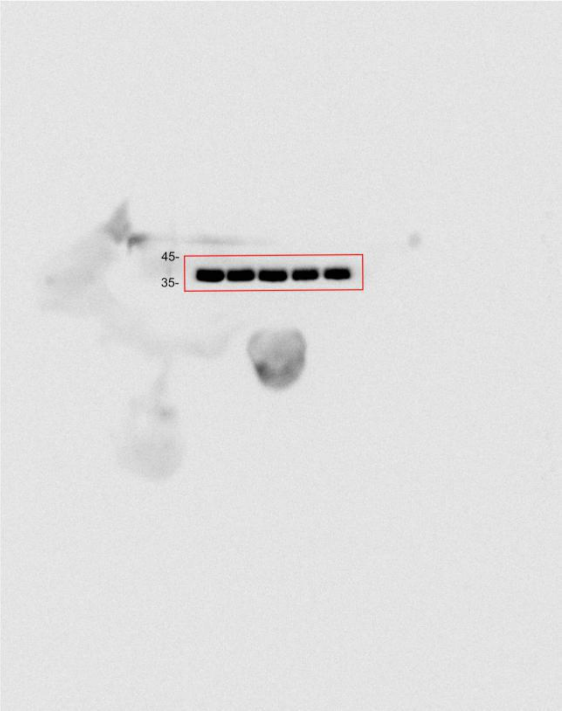

Figure S1B representative image

METTL3

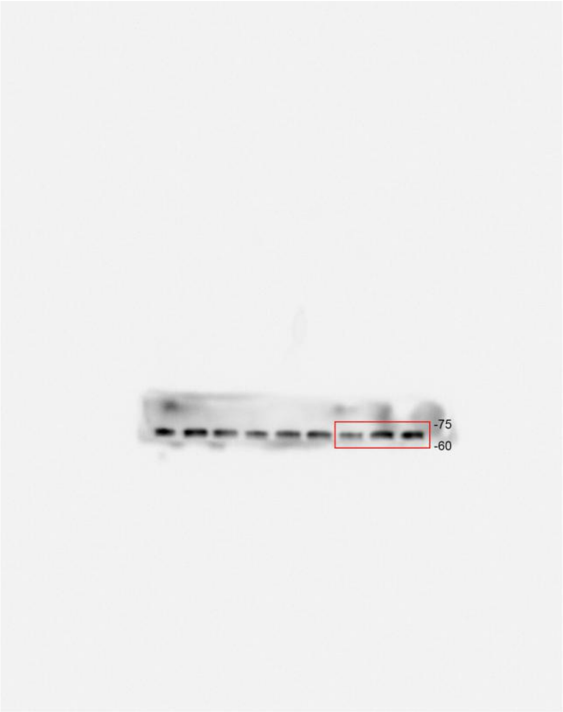

GAPDH

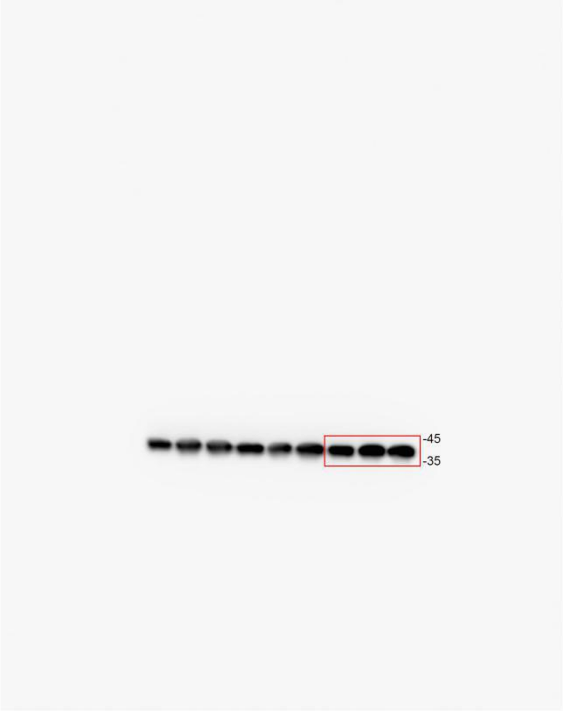

Figure S1E representative image

U251 BUD13

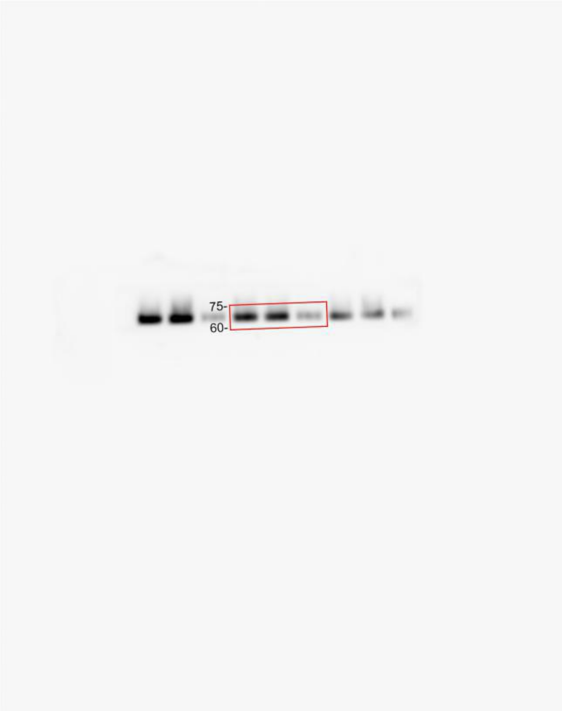

U251 GAPDH

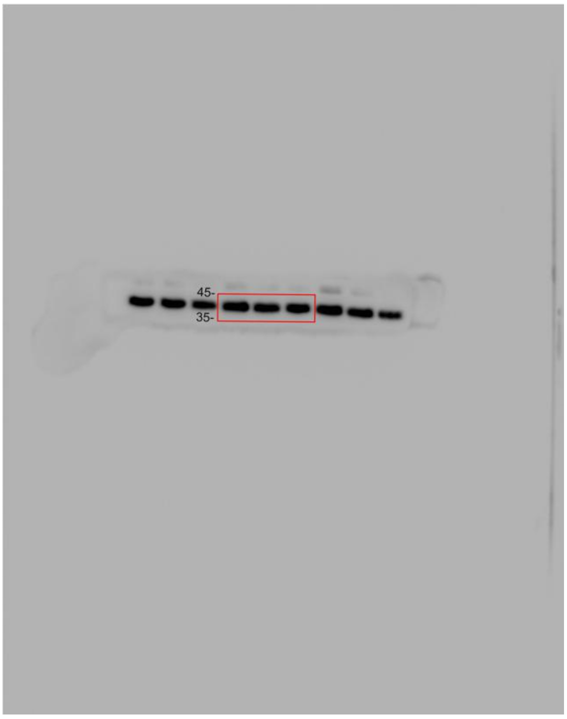

U373 BUD13

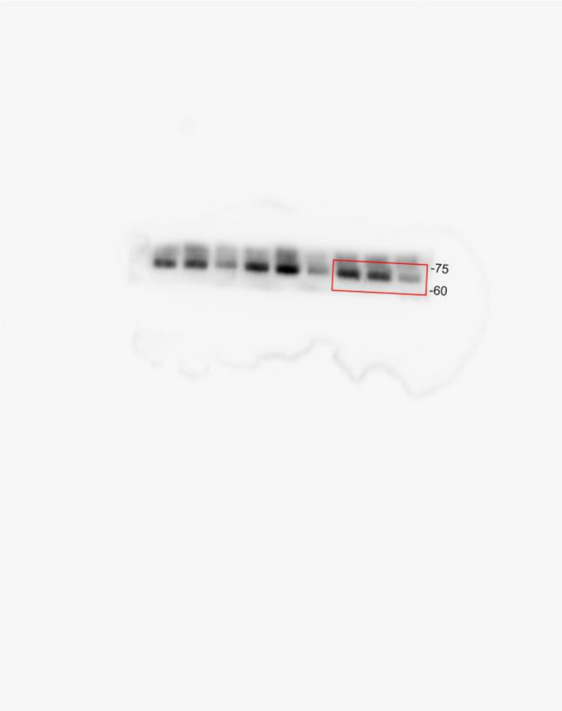

U373 GAPDH

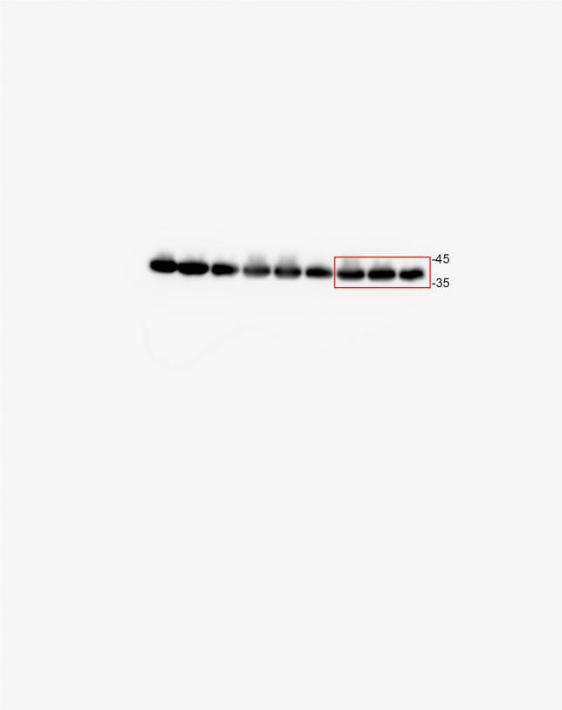

Figure S1I representative image

METTL3

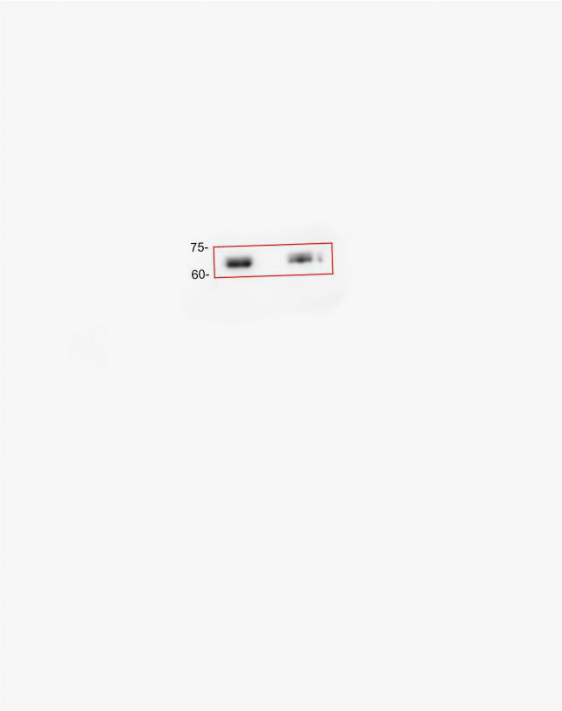

GAPDH

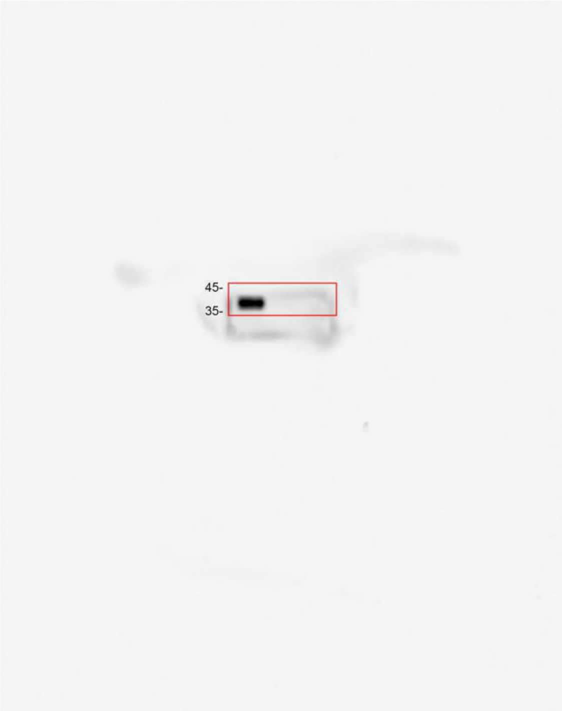

Figure S2D representative image

U251 CDK12

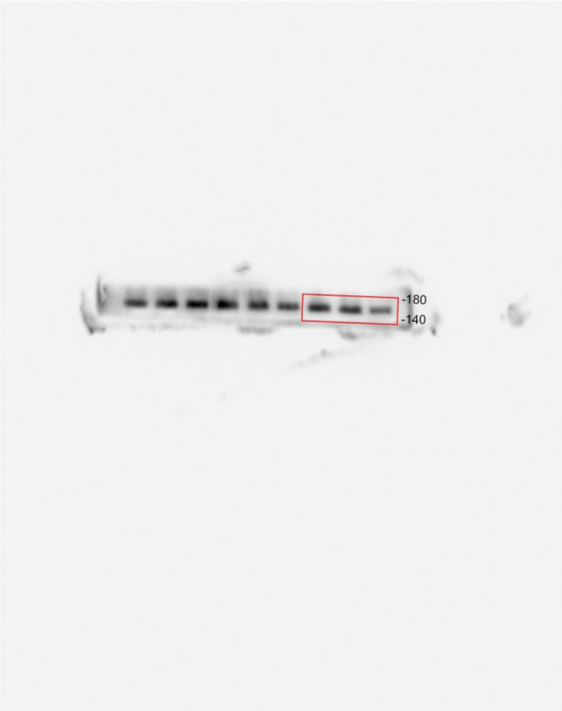

U251 GAPDH

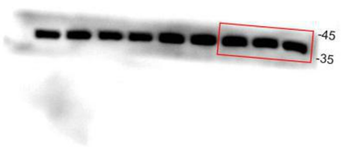

U373 CDK12

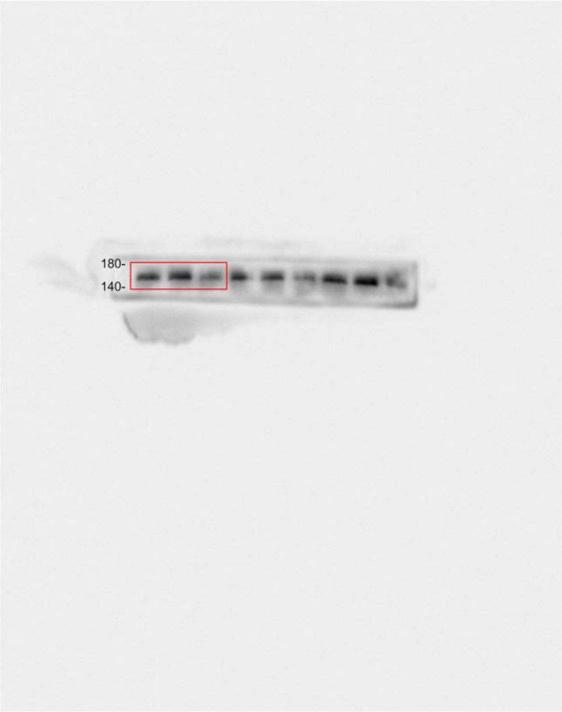

U373 GAPDH

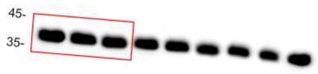

Figure S2G representative image

BUD13

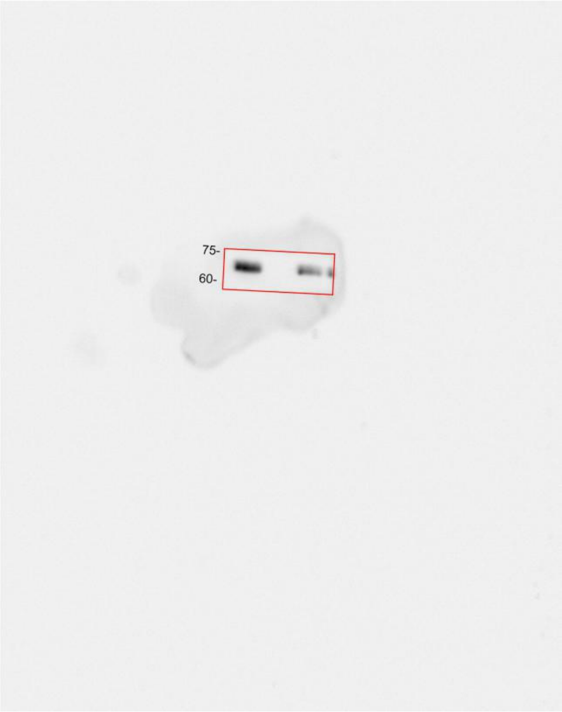

GAPDH

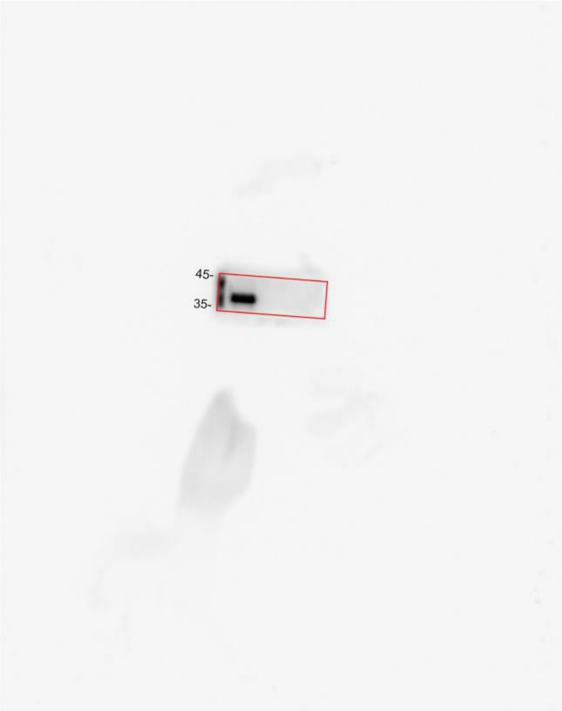

Figure S3E representative image

U251 MMP2

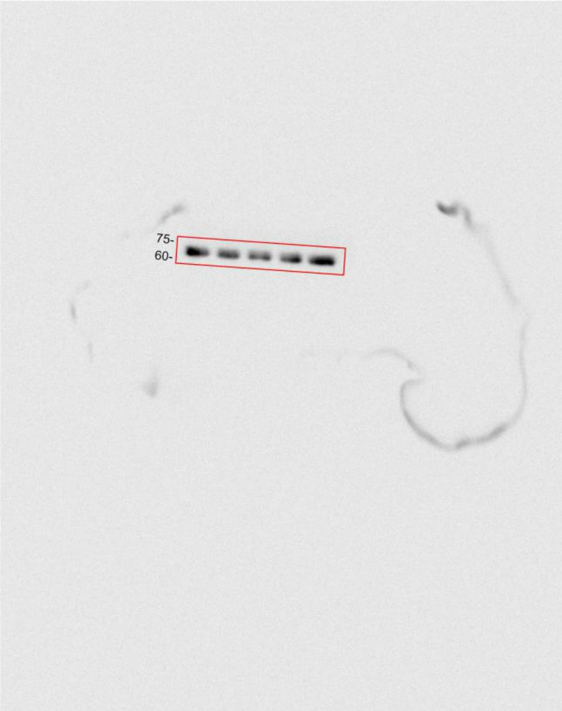

U251 LAMC2

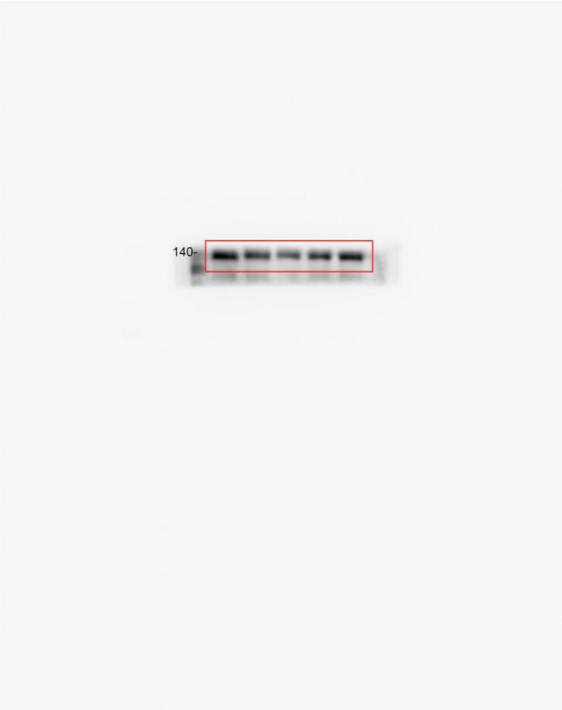

U251 GAPDH

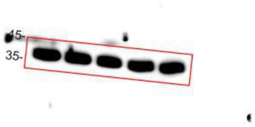

U373 MMP2

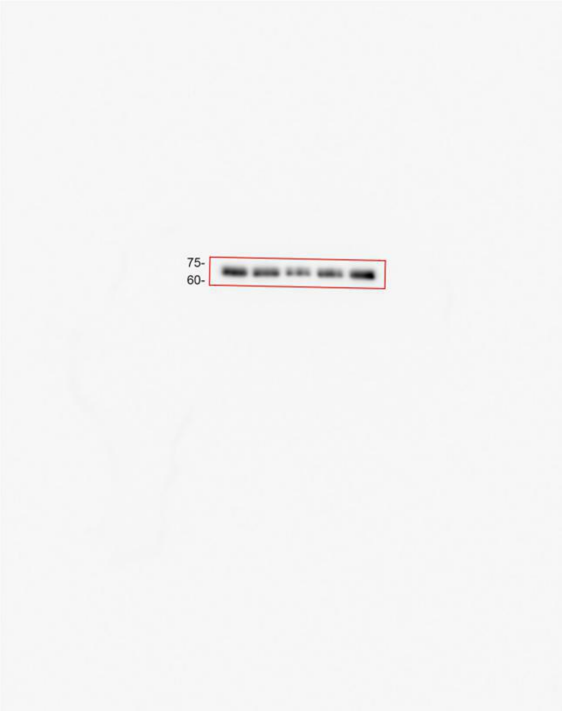

U373 LAMC2

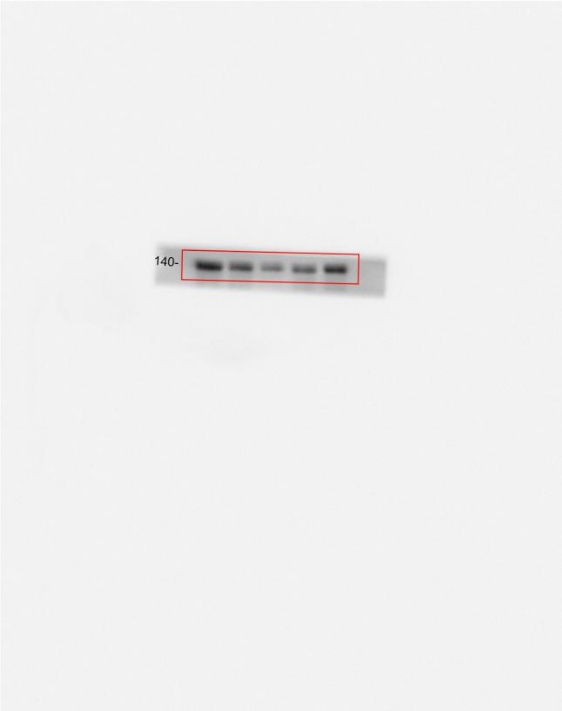

U373 GAPDH

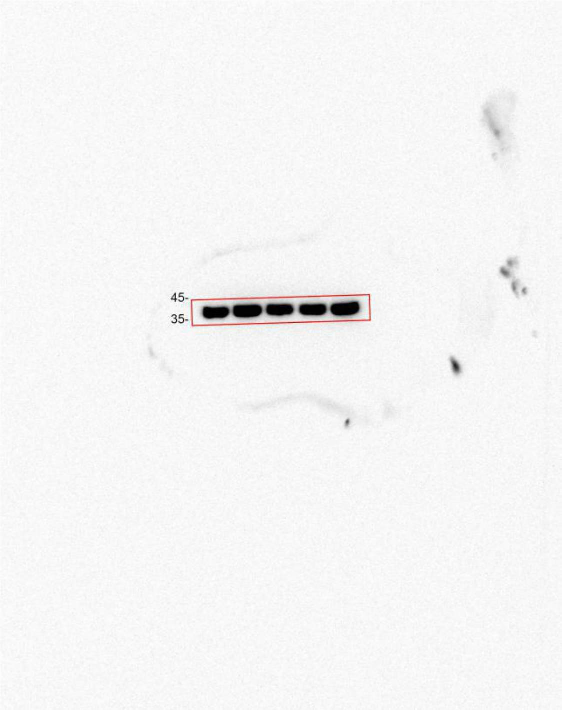

Figure S4E representative image

U251 p-MBNL1

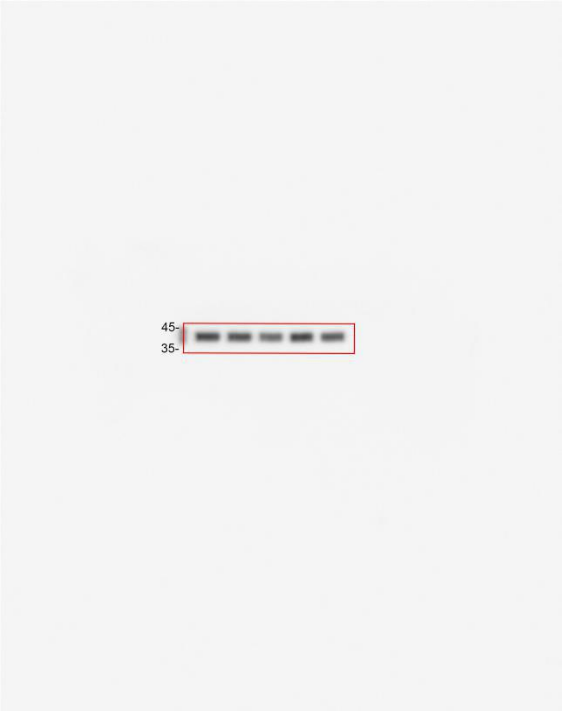

U251 MBNL1

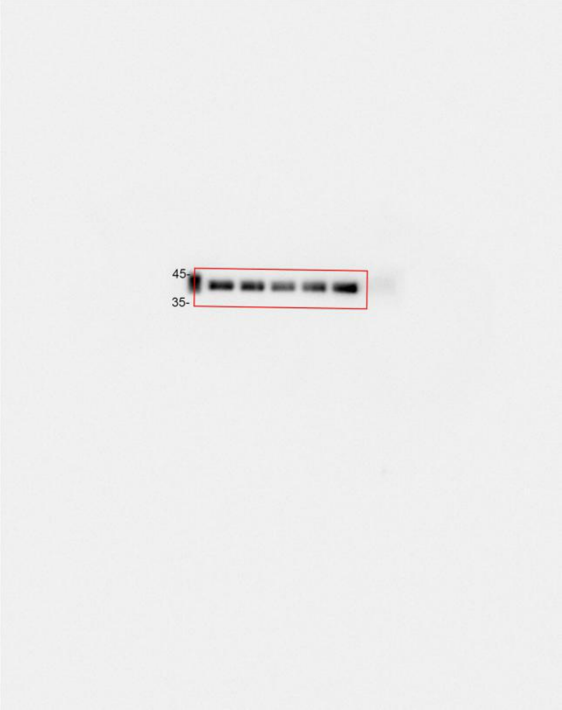

U251 GAPDH

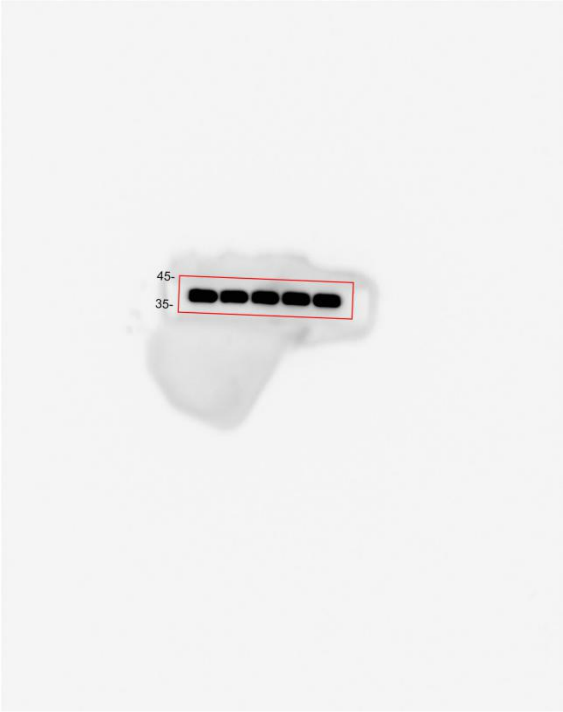

U373 p-MBNL1

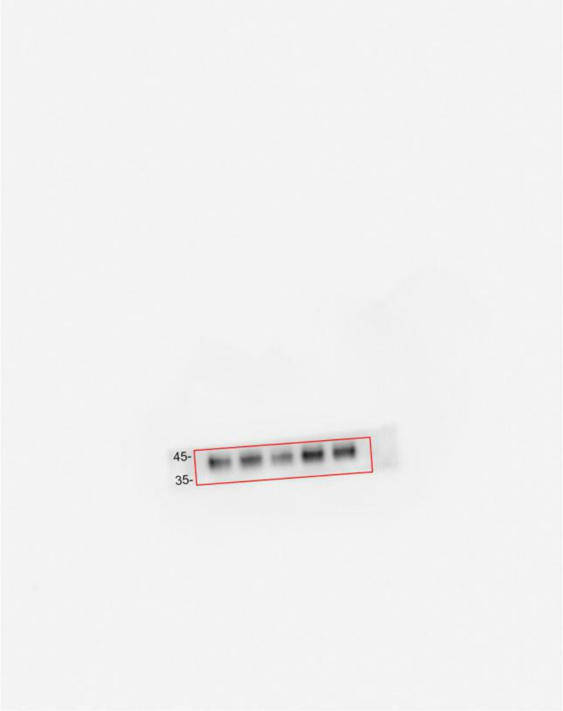

U373 MBNL1

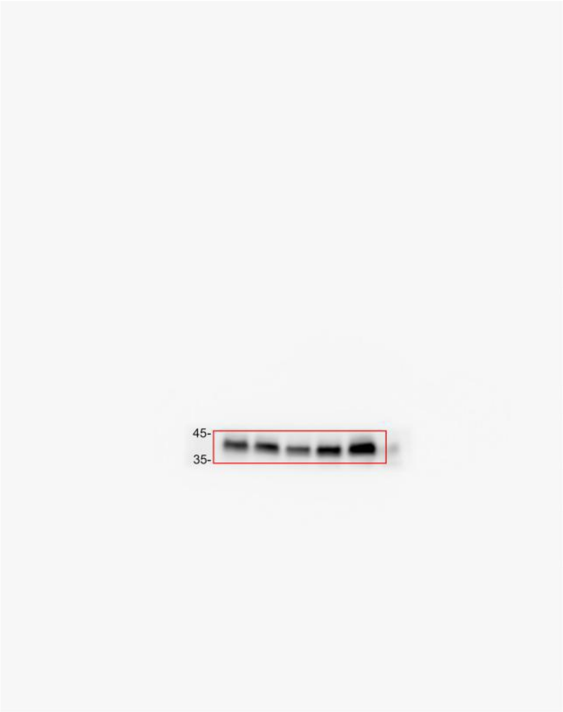

U373 GAPDH

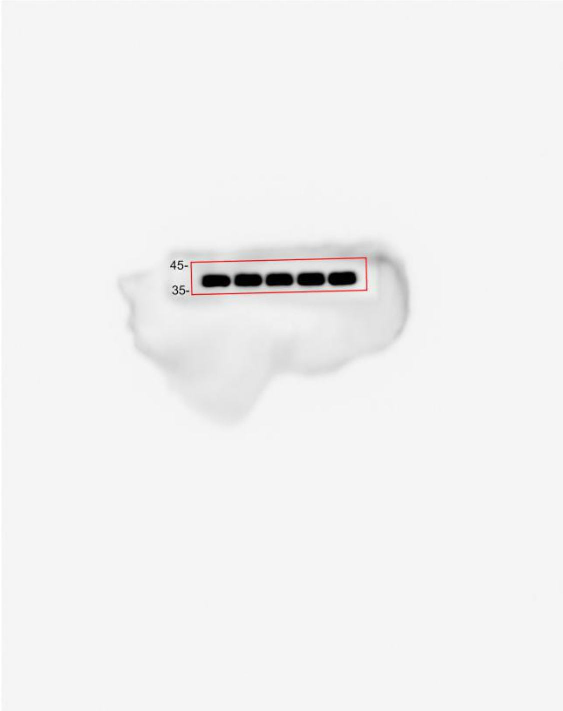

Figure S5E representative image

U251 MMP2

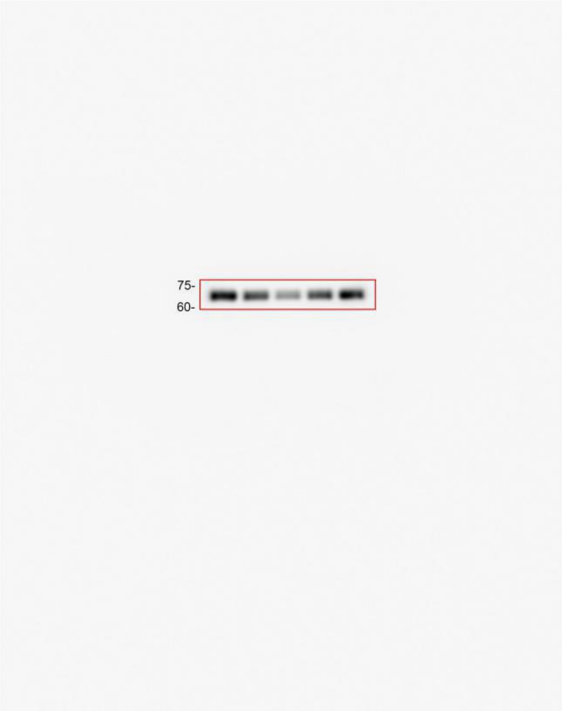

U251 LAMC2

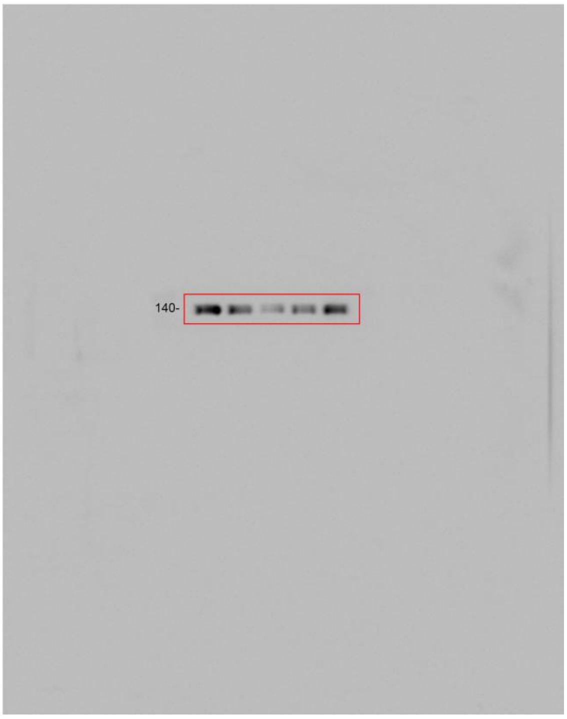

U251 GAPDH

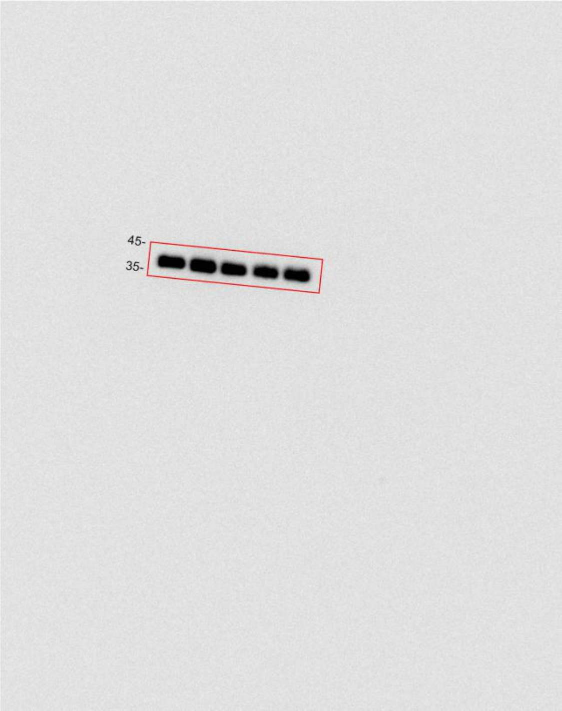

U373 MMP2

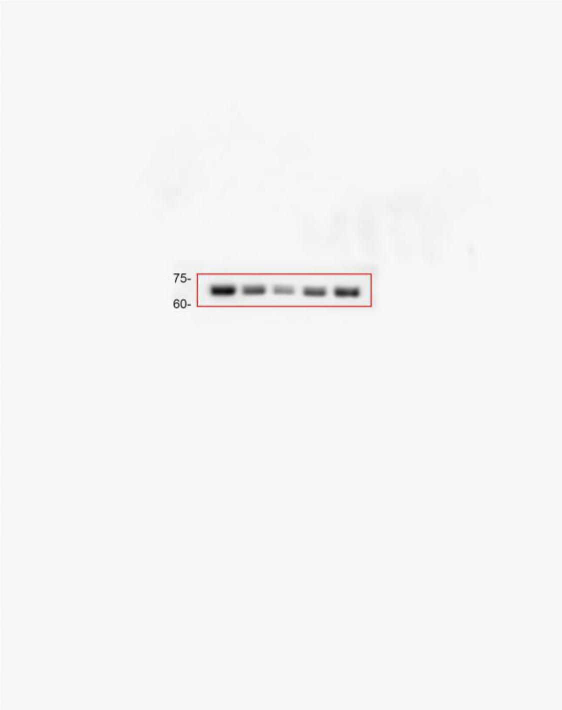

U373 LAMC2

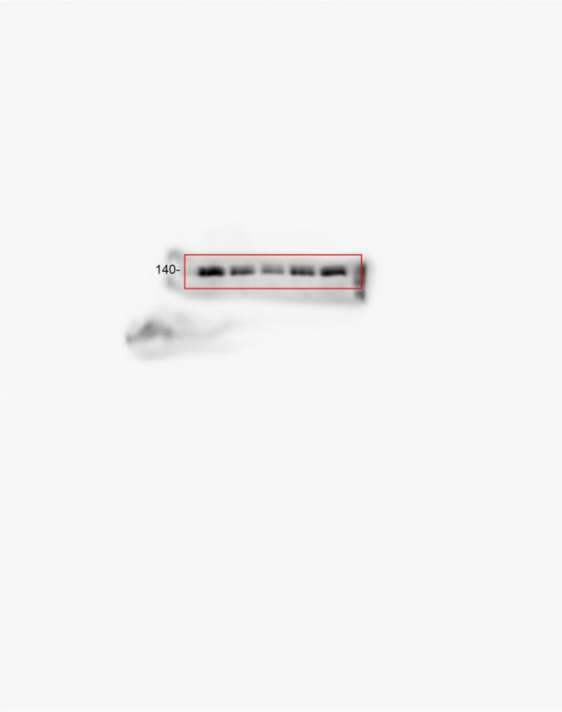

U373 GAPDH

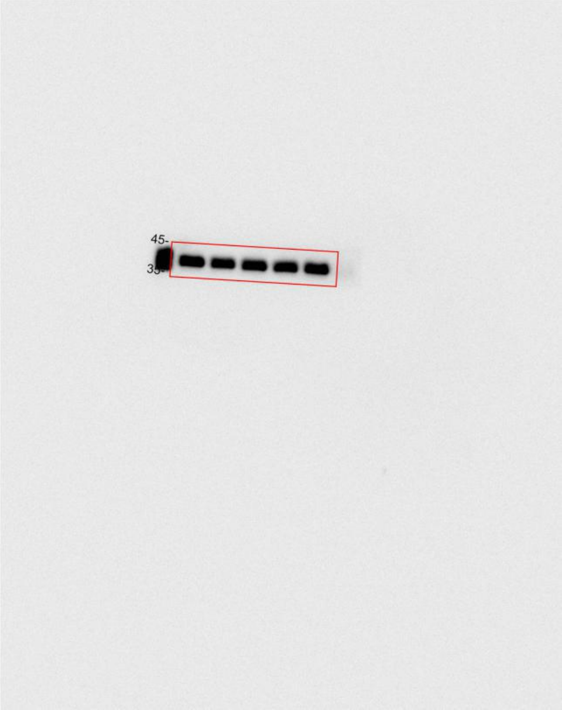

Figure S6A representative image

U251 METTL3

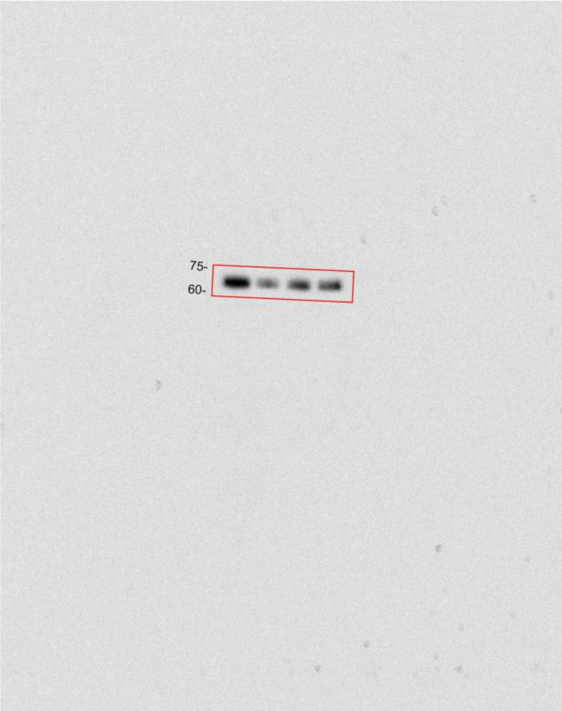

U251 GAPDH

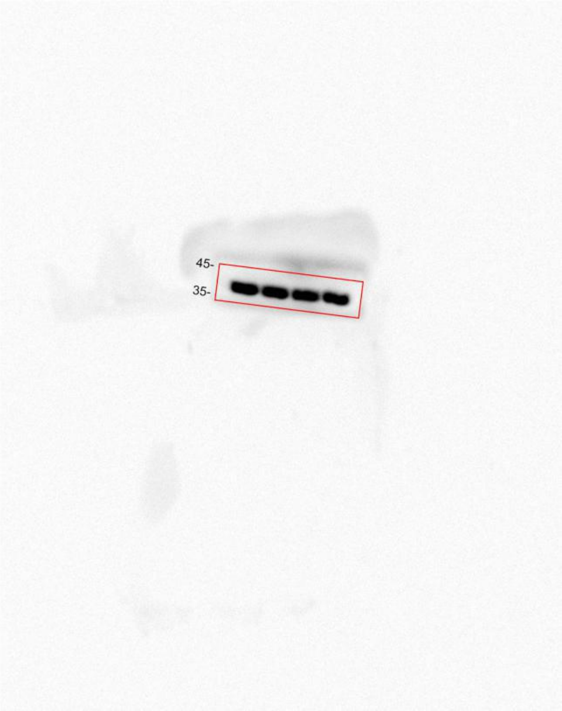

U373 METTL3

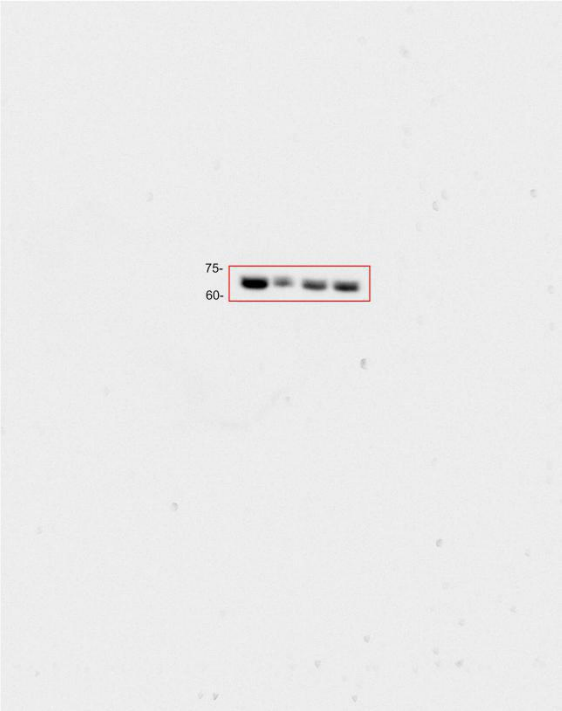

U373 GAPDH

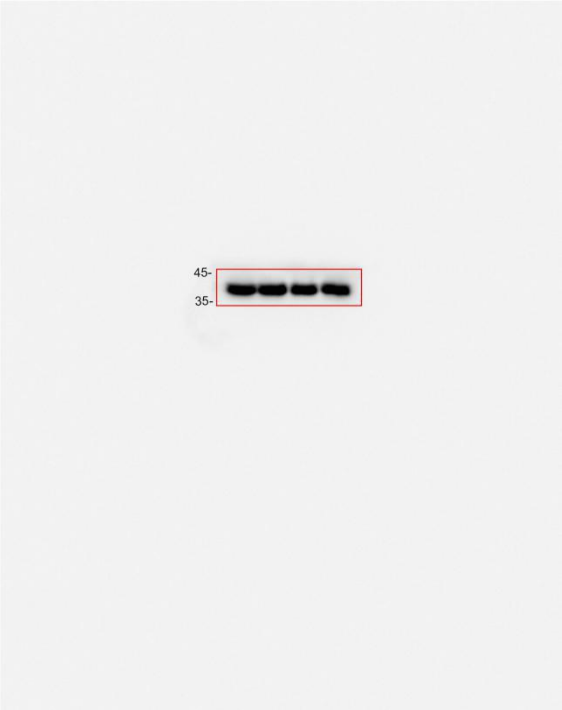

Figure S6B representative image

U251 BUD13

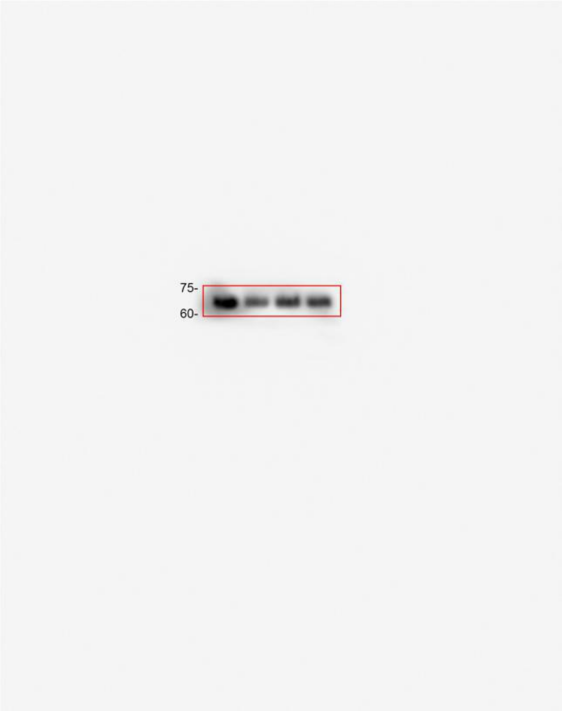

U251 GAPDH

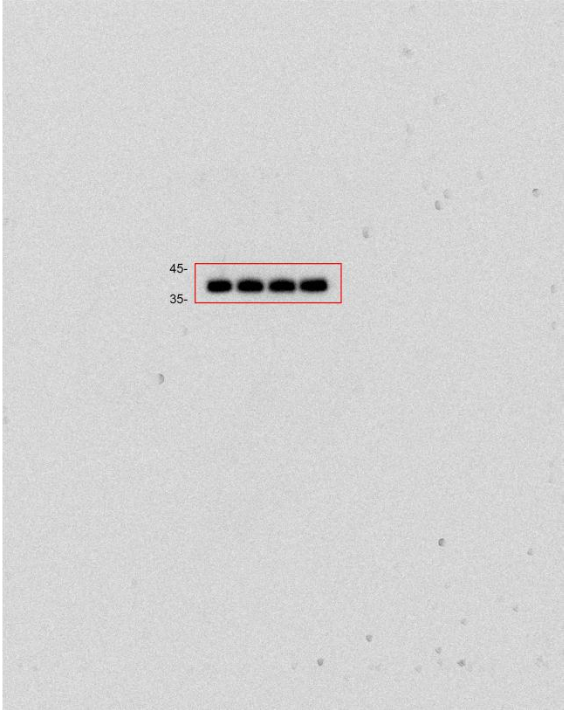

U373 BUD13

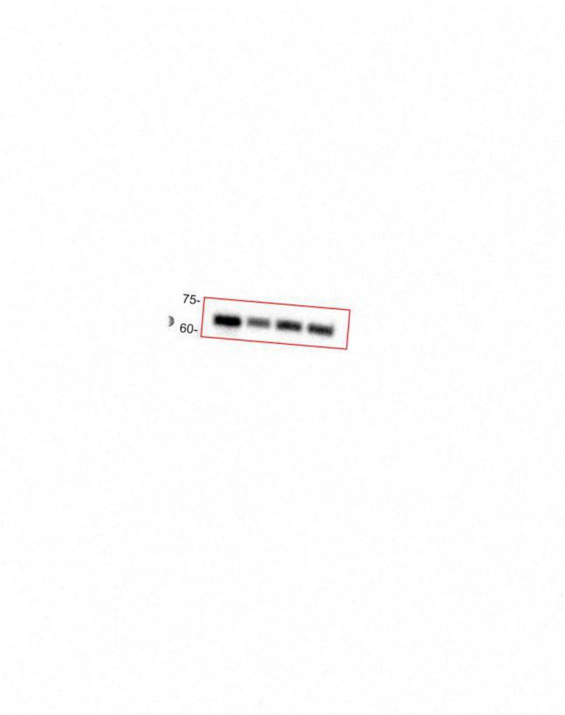

U373 GAPDH

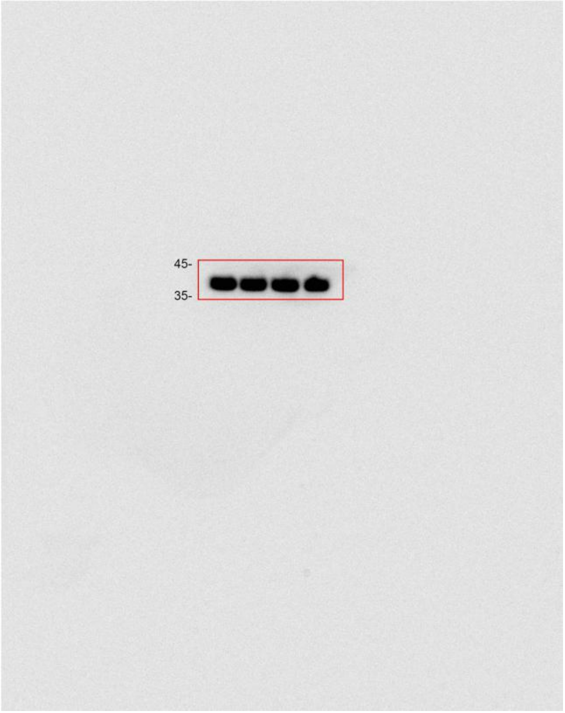

Figure S6C representative image

U251 CDK12

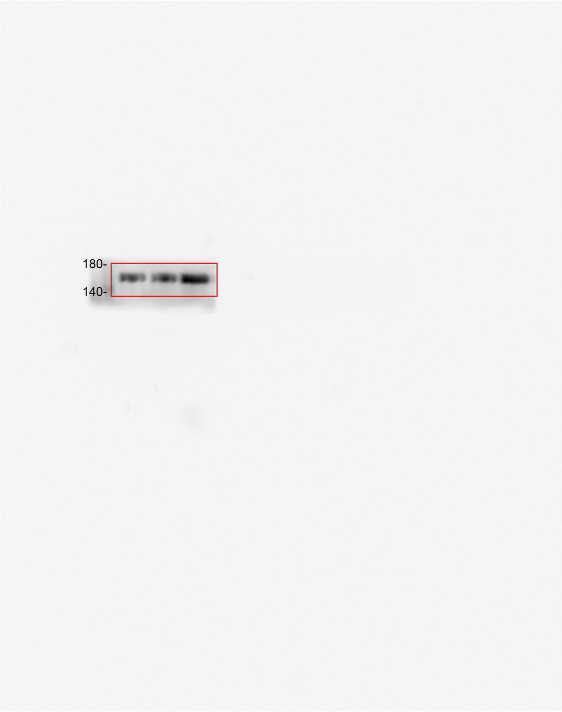

U251 GAPDH

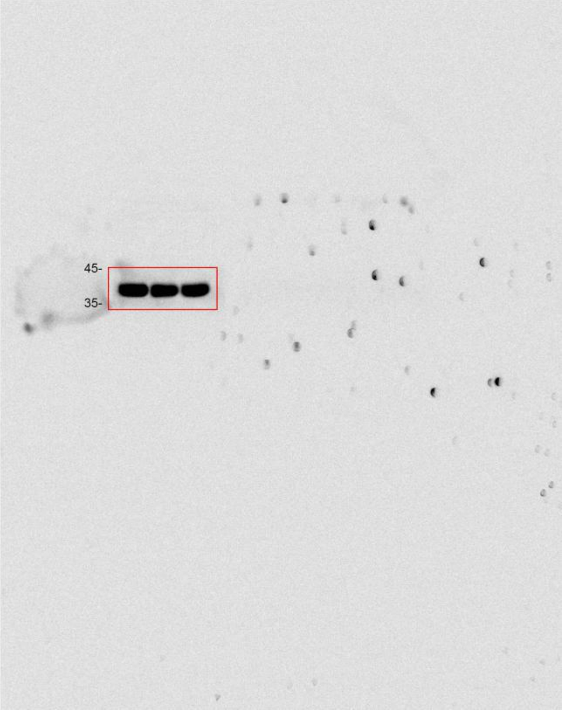

U373 CDK12

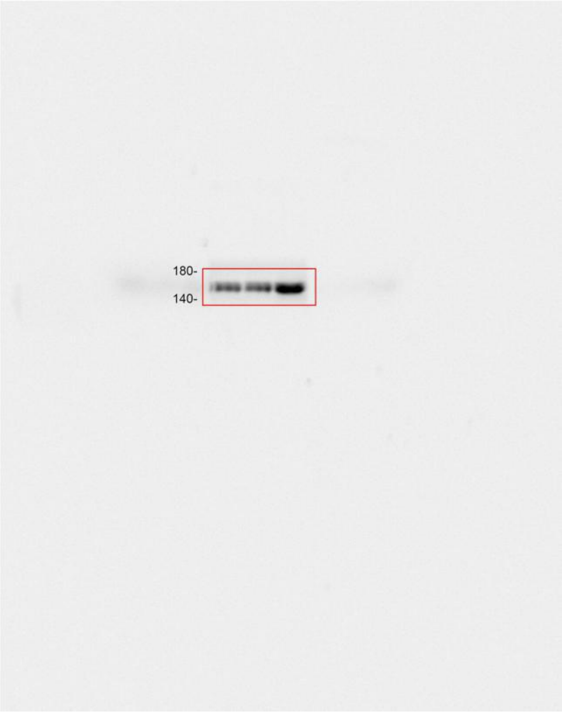

U373 GAPDH

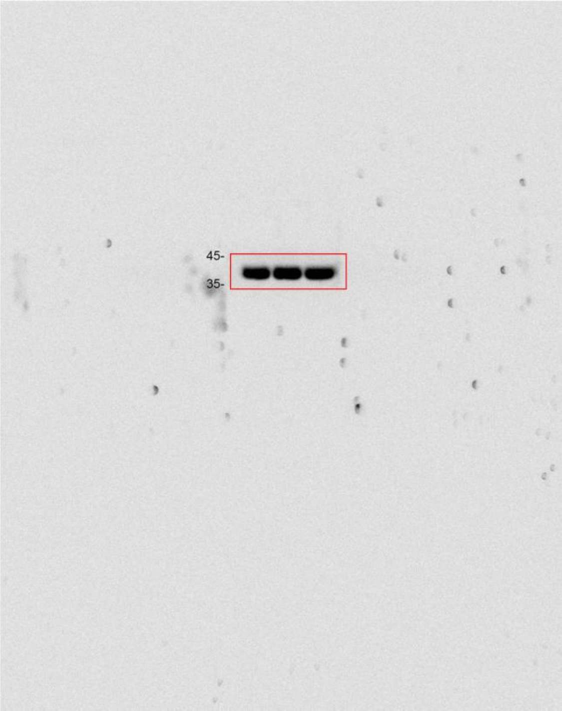

Figure S6D representative image

U251 CDK12

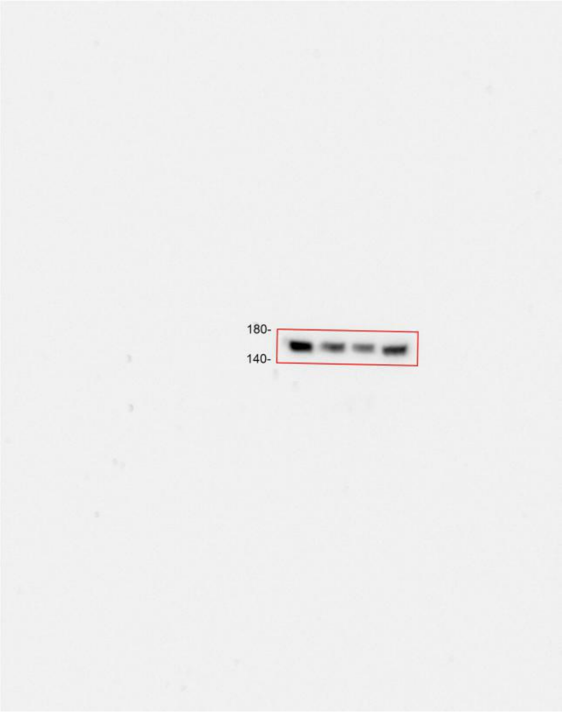

U251 GAPDH

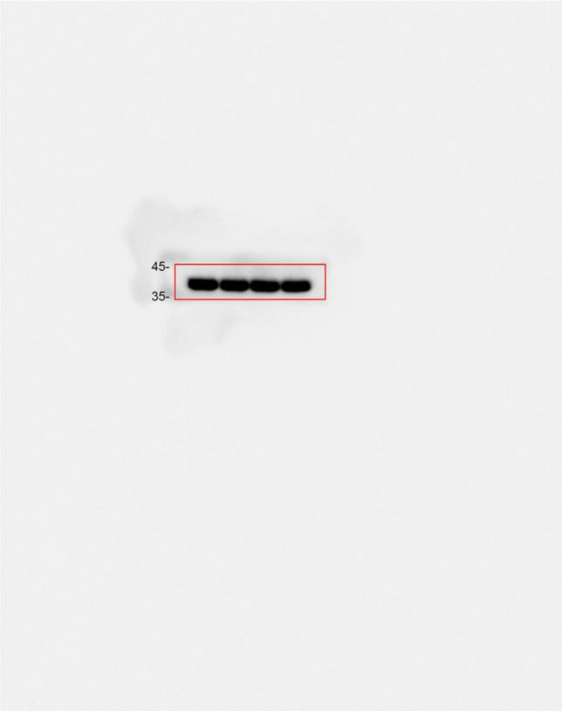

U373 CDK12

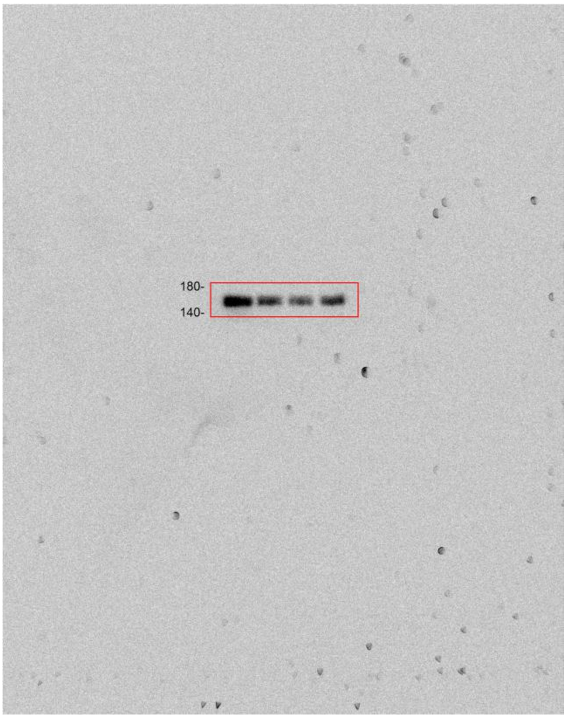

U373 GAPDH

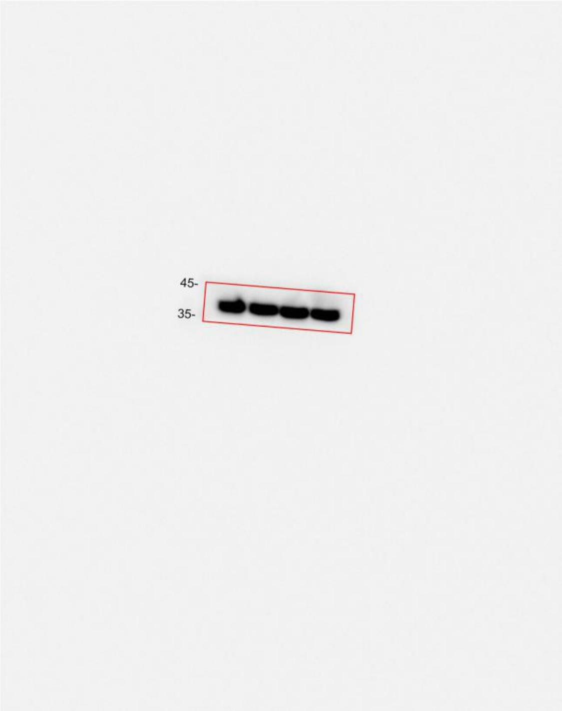

Figure S6E representative image

U251 MBNL1

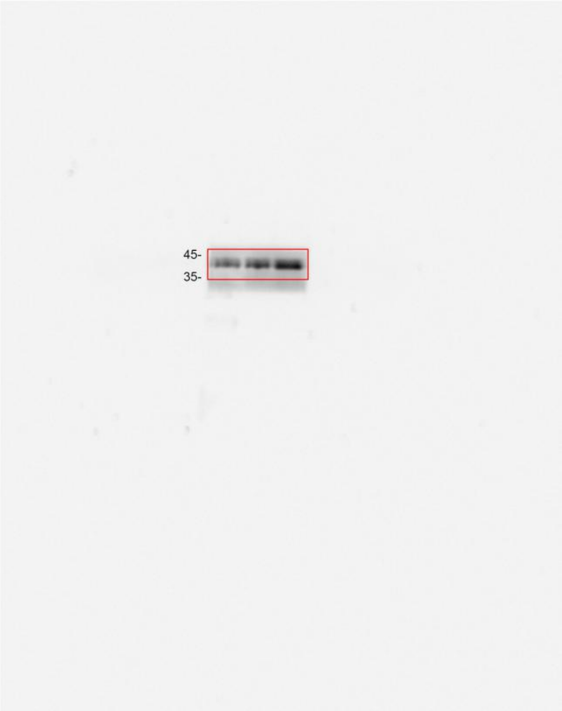

U251 GAPDH

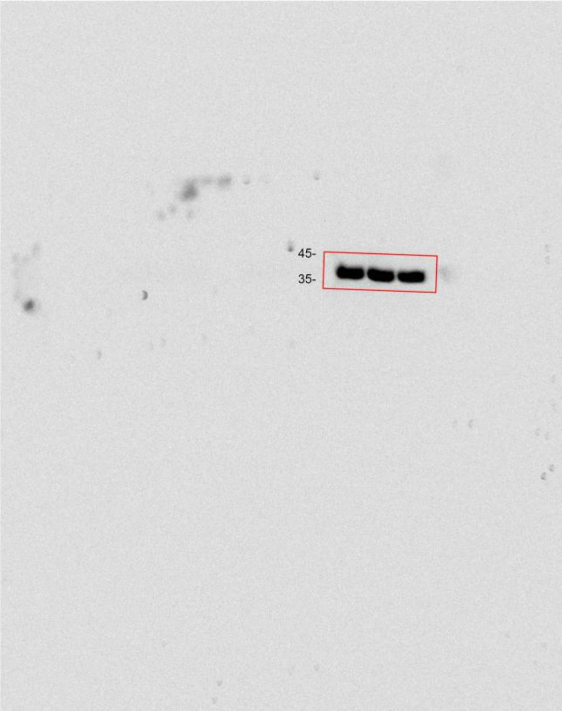

U373 MBNL1

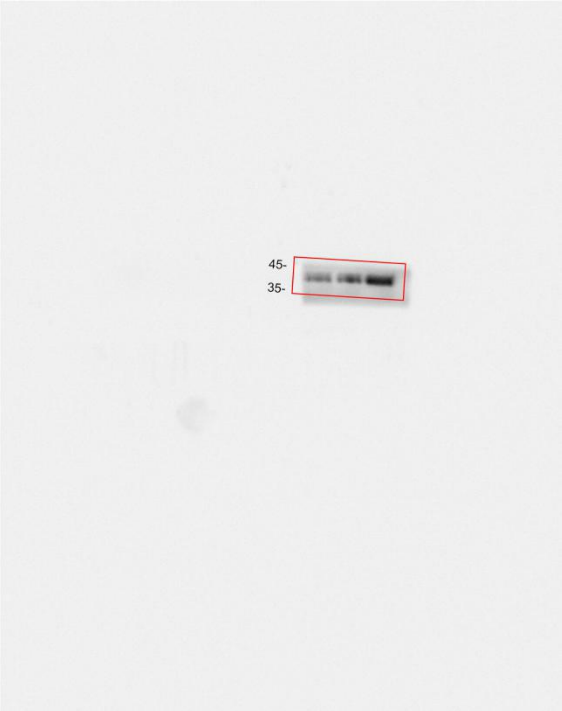

U373 GAPDH

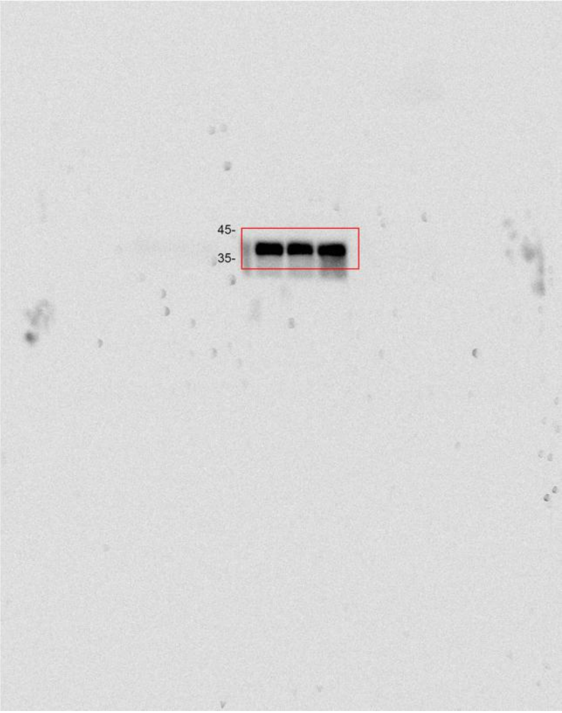

Figure S6F representative image

U251 MBNL1

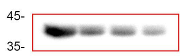

U251 GAPDH

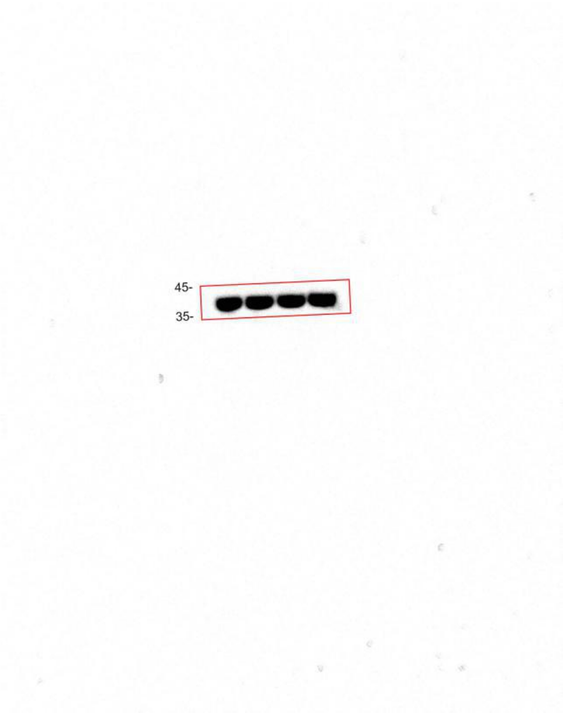

U373MBNL1

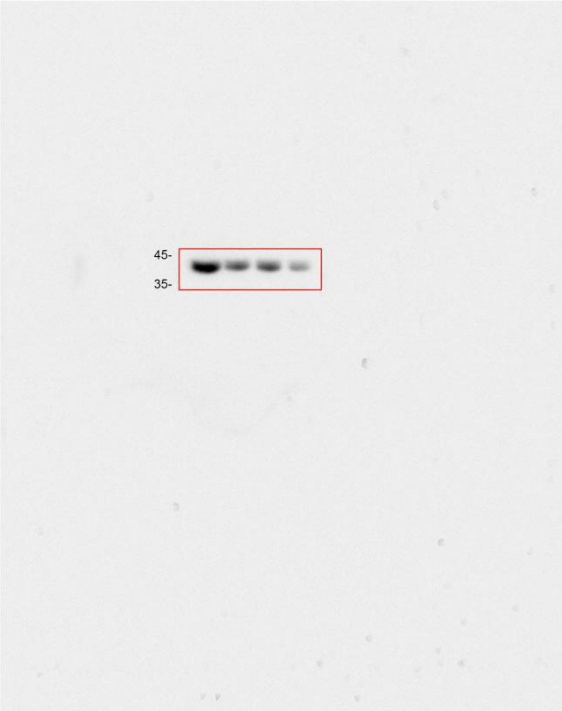

U373 GAPDH

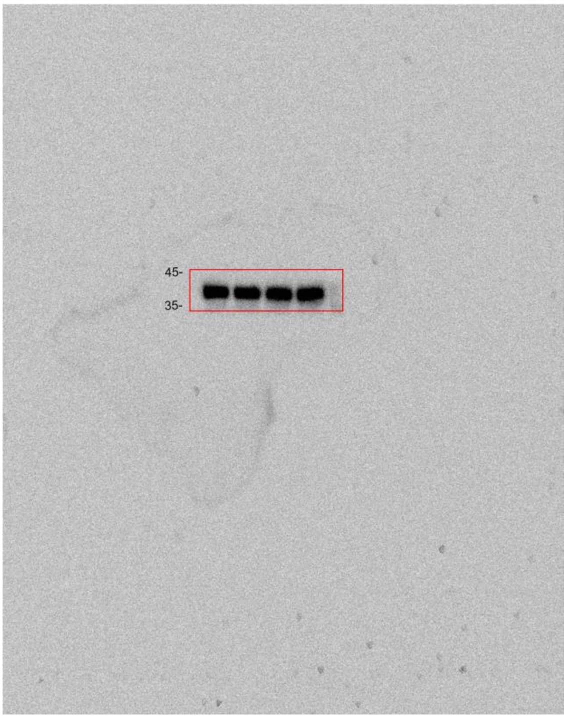

Supplement: Supplementary file 3 — Original western blots [file 41419_2022_5426_MOESM3_ESM.pdf]
