# Supplementary figures and images for: The mechanism of BUD13 m6A methylation mediated MBNL1-phosphorylation by CDK12 regulating the vasculogenic mimicry in glioblastoma cells
Source: Cell Death Dis. 2022 Dec 3;13(12):1017. doi: 10.1038/s41419-022-05426-z (PMC9719550; doi:10.1038/s41419-022-05426-z)

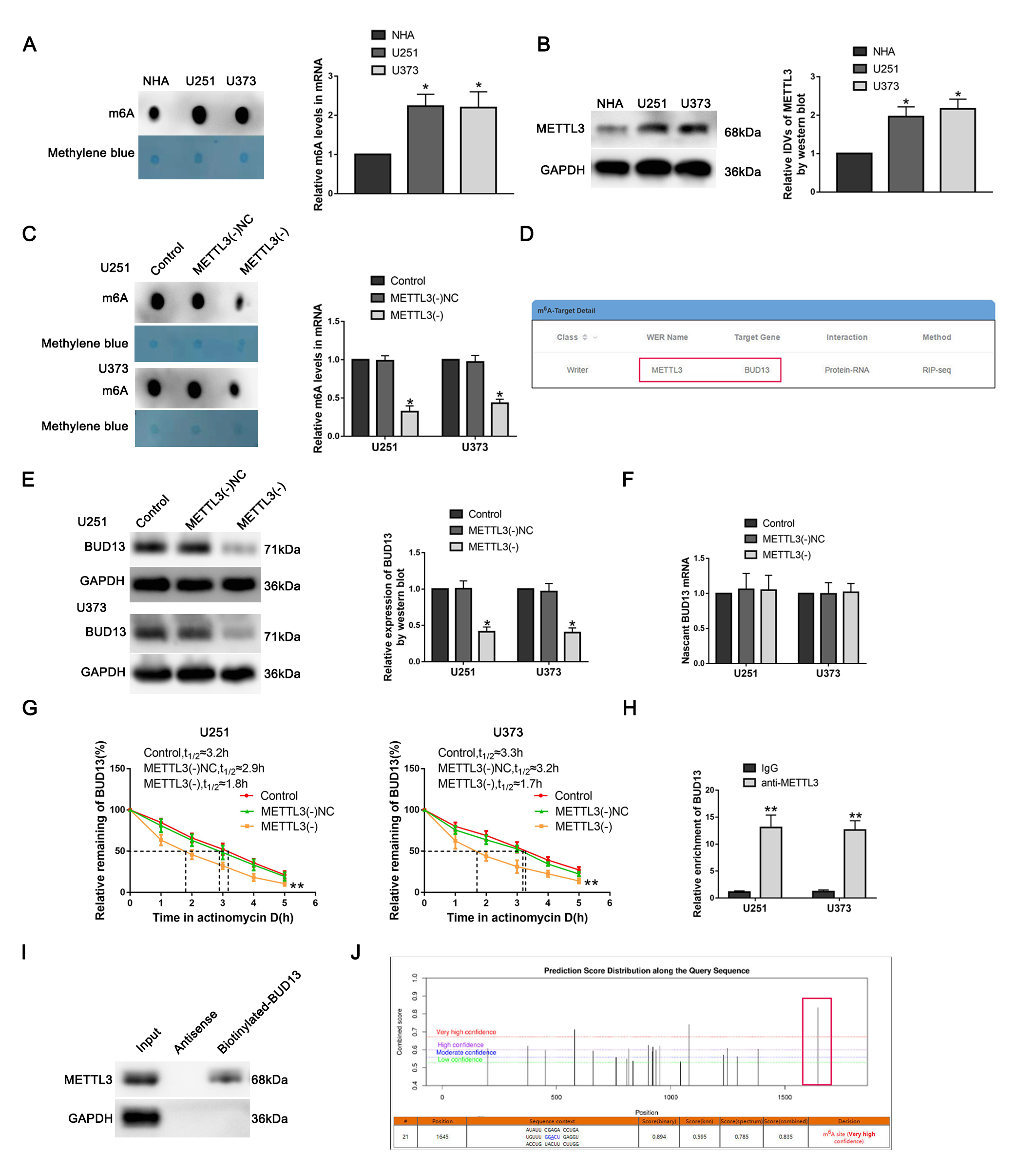

Supplement: Supplementary file 5 — Figure S1 [file 41419_2022_5426_MOESM5_ESM.tif]

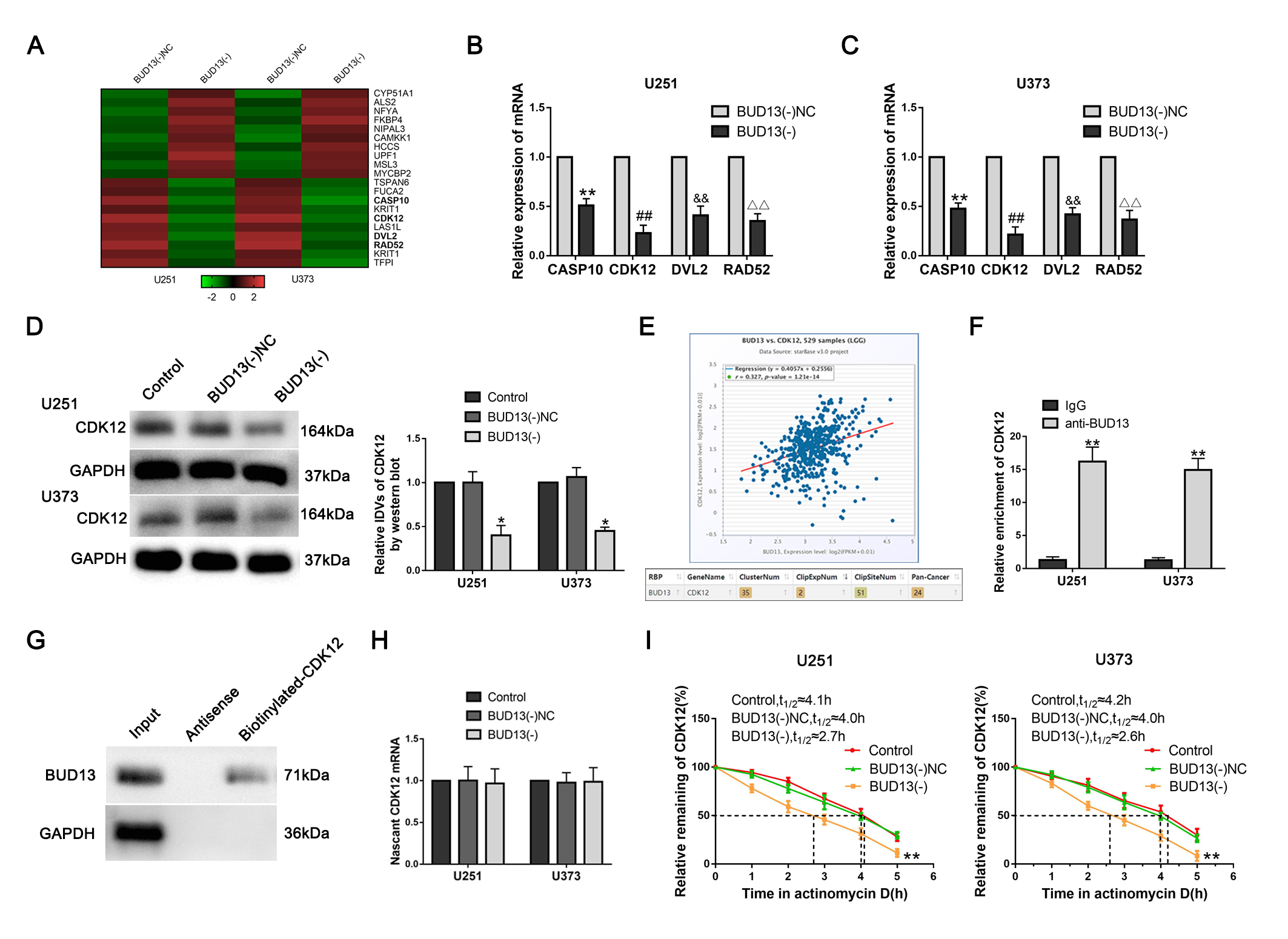

Supplement: Supplementary file 6 — Figure S2 [file 41419_2022_5426_MOESM6_ESM.tif]

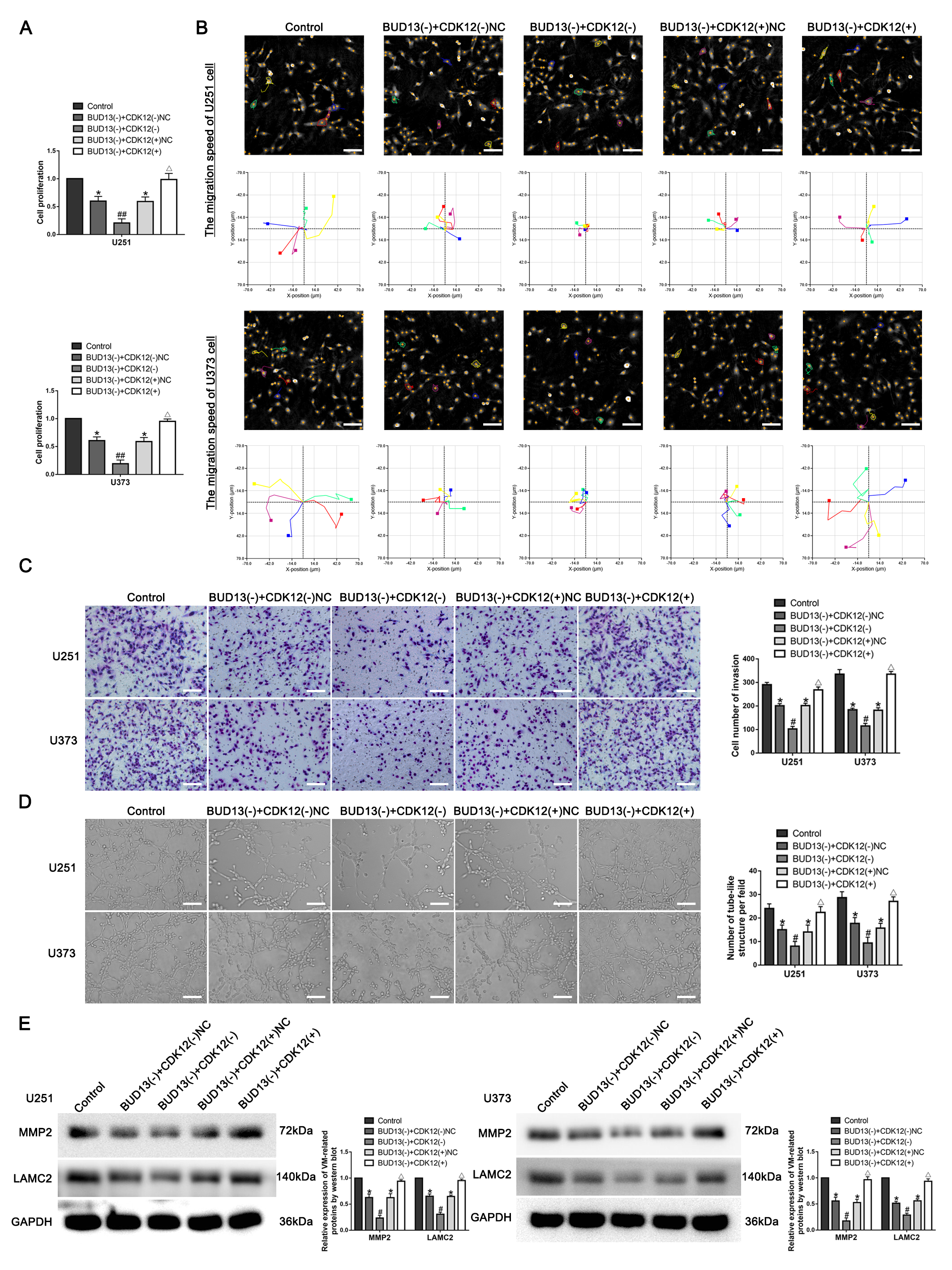

Supplement: Supplementary file 7 — Figure S3 [file 41419_2022_5426_MOESM7_ESM.tif]

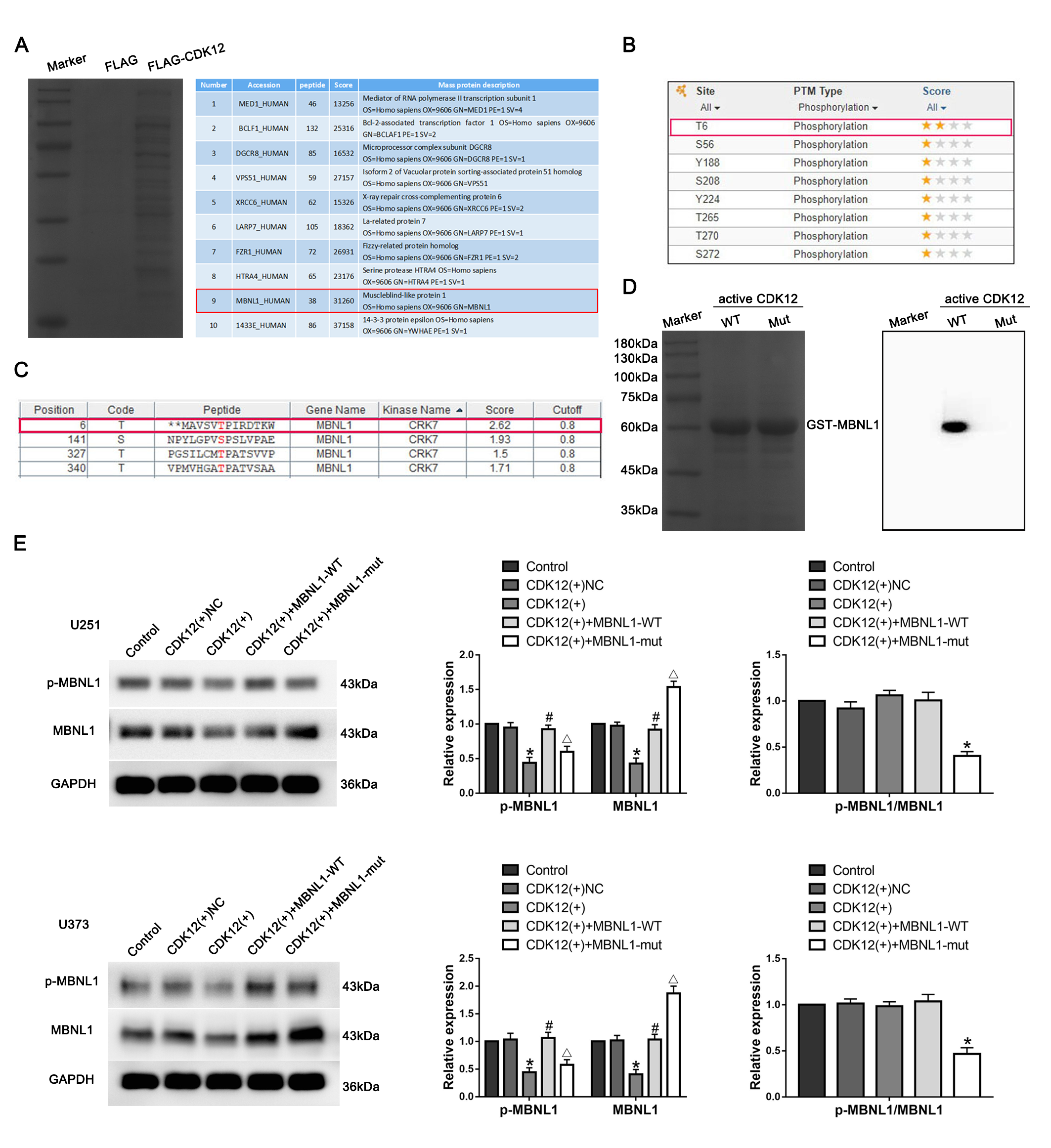

Supplement: Supplementary file 8 — Figure S4 [file 41419_2022_5426_MOESM8_ESM.tif]

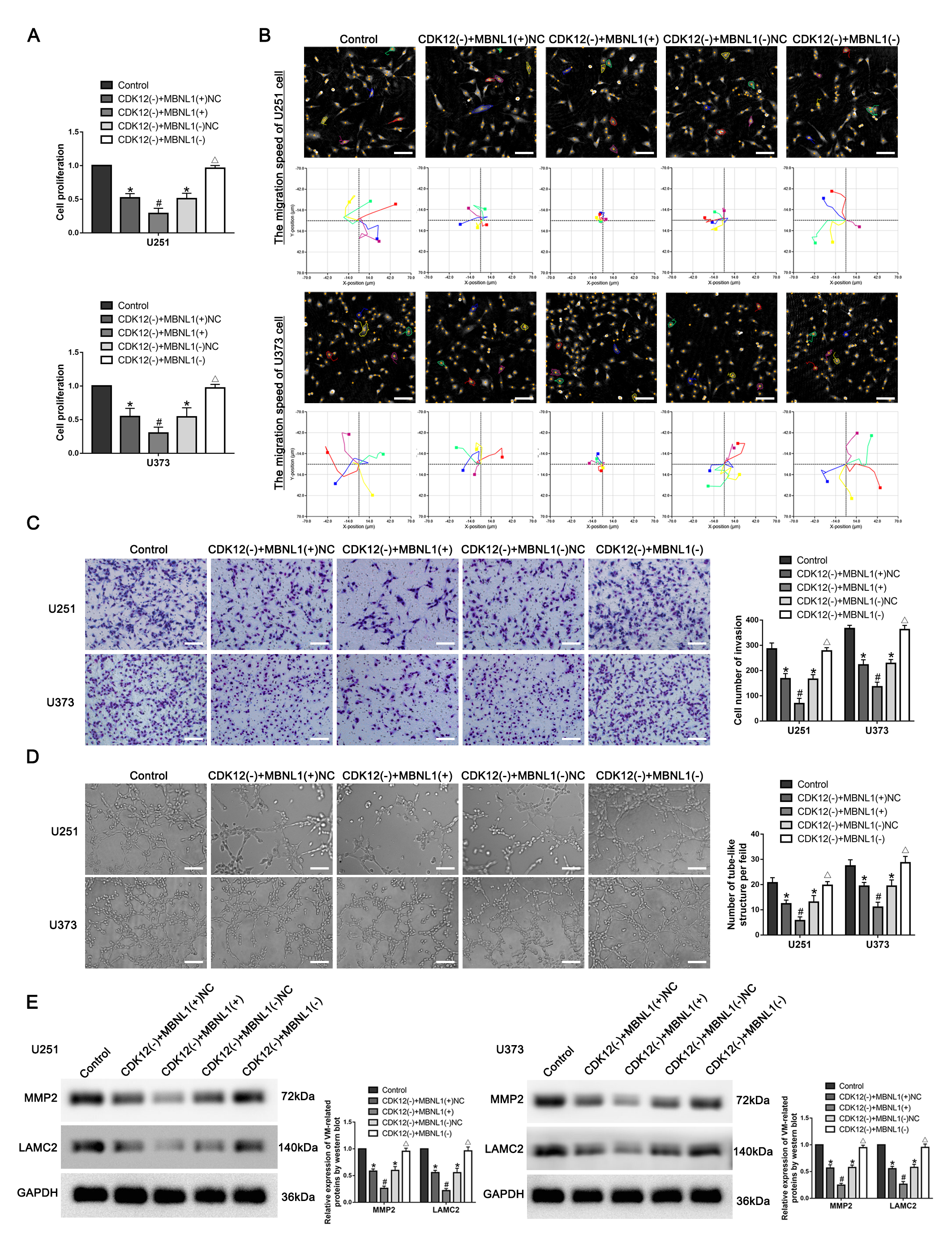

Supplement: Supplementary file 9 — Figure S5 [file 41419_2022_5426_MOESM9_ESM.tif]

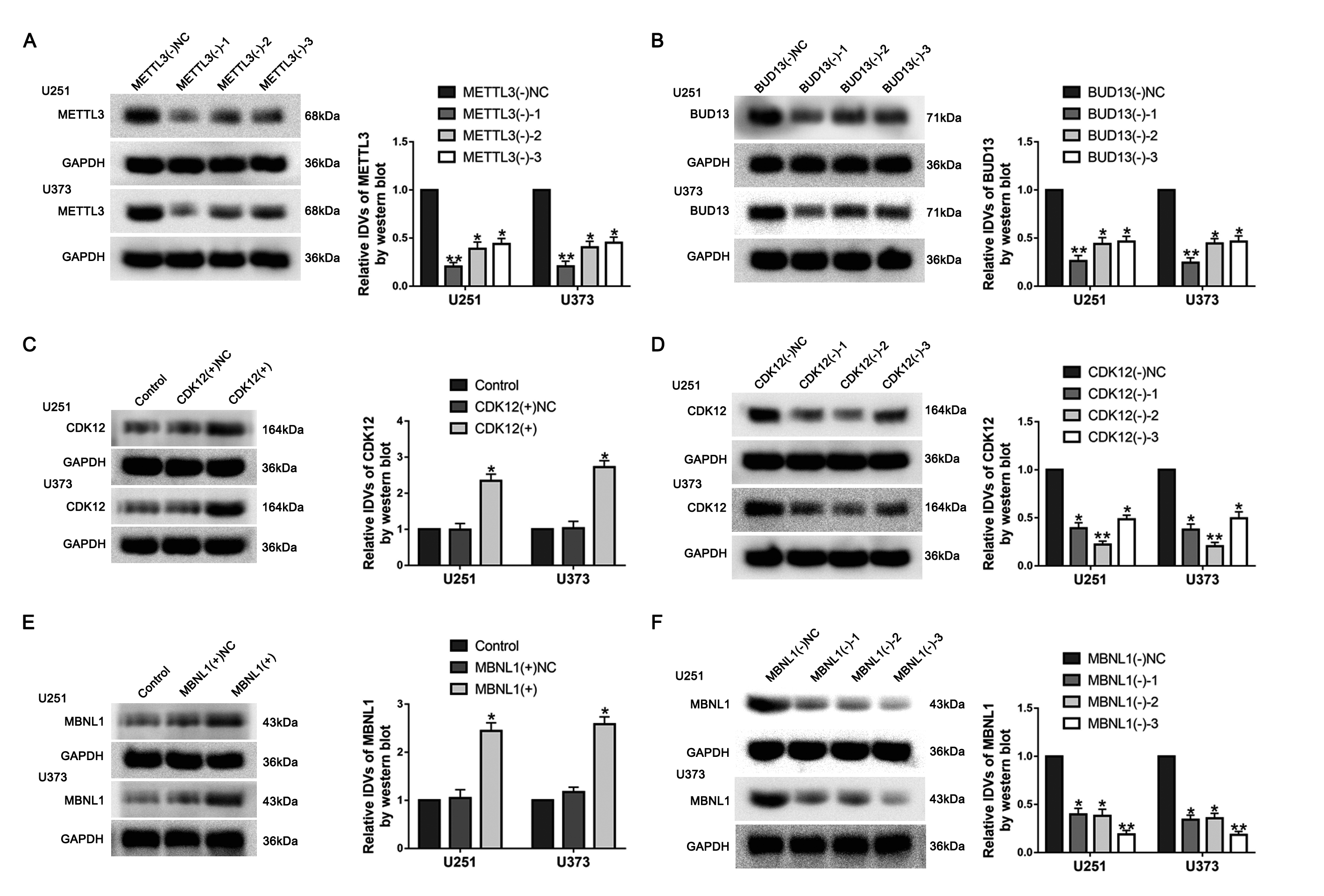

Supplement: Supplementary file 10 — Figure S6 [file 41419_2022_5426_MOESM10_ESM.tif]
