# Supplementary material for: The mechanism of BUD13 m6A methylation mediated MBNL1-phosphorylation by CDK12 regulating the vasculogenic mimicry in glioblastoma cells
Source: Cell Death Dis. 2022 Dec 3;13(12):1017. doi: 10.1038/s41419-022-05426-z (PMC9719550; doi:10.1038/s41419-022-05426-z)
Supplement: Supplementary file 11 — Supplementary Information [file 41419_2022_5426_MOESM11_ESM.docx]

Supplementary Figure

Figure S1 METTL3 methylated BUD13 to enhance its stability. (A) Dot blot was used to detect m6A methylation levels in NHAs, U251, and U373 cells. Compared with NHAs group, the m6A methylation level in U251 and U373 cells were significantly increased (n=3). (B) Western blot was used to detect METTL3 protein expression level in NHAs, U251, and U373 cells. Data are presented as mean±SD (n=3). ^*^*P*<0.05, compared with NHAs group. (C) The effect of METTL3 on the m6A methylation level of U251 and U373 cells was detected by Dot blot. Compared with METTL3(-)NC group, the m6A methylation level of METTL3(-) group was significantly decreased. (D) m6A2Target predicted that METTL3 could methylate BUD13. (E) Western blot was used to detect the effect of METTL3 on BUD13 protein expression. Data are presented as mean±SD (n=3). ^*^*P*<0.05, compared with METTL3(-)NC group. (F) Expression of nascent BUD13 was measured via qRT-PCR after METTL3 was knockdown. (G) Half-life of BUD13 was measured by qRT-PCR after actinomycin D was treated in U251 and U373 cells. ^**^*P*<0.01, compared with METTL3(-)NC group. (H) An enrichment of BUD13 mRNA in METTL3 immunoprecipitated samples via RNA immunoprecipitation (RIP) assay. ^**^*P*<0.01, compared with the anti-IgG group, using Student’s t-test. (I) RNA pull-down assay followed by Western blot showed the specific associations of METTL3 with biotinylated-BUD13 or antisense RNA. (J) m6A SRAMP predicted BUD13 m6A methylation sites.

Figure S2 Binding of BUD13 to CDK12 mRNA enhanced its stability. (A) mRNA microarray was performed to detect the differential gene when BUD13 was knockdown. (B and C) Selected molecules were validated by qRT-PCR. ^**/##/&&/ΔΔ^*P*<0.01, compared with BUD13(-)NC group. (D) Western blot detected the effect of BUD13 knockdown on CDK12 protein expression. Data are presented as mean±SD (n=3). ^*^*P*<0.05, compared with BUD13(-)NC group. (E) STARBASE database predicted BUD13 bound to CDK12 mRNA. (F) RIP assay analyzed the enrichment of CDK12 mRNA in BUD13 immunoprecipitates. ^**^*P*<0.01 compared with the anti-IgG group. (G) RNA pull-down assay detected the binding between BUD13 and CDK12 mRNA. (H) Expression of nascent CDK12 was measured via qRT-PCR after BUD13 was knockdown. (I) Half-life of CDK12 was measured by qRT-PCR after actinomycin D was treated in U251 and U373 cells. ^**^*P*<0.01, compared with BUD13(-)NC group.

Figure S3 Effects of BUD13 and CDK12 on VM formation in GBM cells. (A) The effects of BUD13 and CDK12 on the proliferation of U251 and U373 were detected by CCK8 assay. Data are presented as mean±SD (n=3). ^*^*P*<0.05, compared with the Control group. ^##^*P*<0.01,compared with BUD13(-)+CDK12(-)NC group. ^Δ^*P*<0.05, compared with BUD13(-)+CDK12(+)NC group. (B) Hstudio M4 detected the effect of BUD13 and CDK12 on the migration of U251 and U373 cells (n=5). (C) Transwell assay was used to detect the effects of BUD13 and CDK12 on the invasion of U251 and U373 cells. Data are presented as mean±SD (n=3). ^*^*P*<0.05, compared with the Control group. ^#^*P*<0.05, compared with BUD13(-)+CDK12(-)NC group; ^△^*P*<0.05, compared with BUD13(-)+CDK12(+)NC group. Scale bar: 50 μm. (D) Three-dimensional tube formation assay detected the effects of BUD13 and CDK12 on tube formation of U251 and U373 cells. Data are presented as mean±SD (n=3). ^*^*P*<0.05, compared with the Control group. ^#^*P*<0.05, compared with BUD13(-)+CDK12(-)NC group; ^△^*P*<0.05, compared with BUD13(-)+CDK12(+)NC group. Scale bar: 50μm. (E) Western blot detected the effects of BUD13 and CDK12 on VM-related proteins MMP2 and LAMC2 expression in U251 and U373 cells. Data are presented as mean±SD (n=3). ^*^*P*<0.05, compared with the Control group. ^#^*P*<0.05, compared with BUD13(-)+CDK12(-)NC group; ^△^*P*<0.05, compared with BUD13(-)+CDK12(+)NC group.

Figure S4 Screening for MBNL1 and the effect of MBNL1 T6 phosphorylation site mutation on phosphorylation and MBNL1 and p-MBNL1 protein expression. (A) Left panel, Coomassie brilliant blue (CBB) staining of precipitates immunoprecipitated by FLAG tag antibodies from U251 cells transfected with FLAG-CDK12 or empty plasmid. Right panel, CDK12-interacting proteins screened by mass spectrometry analysis of the precipitates, and MBNL1 was marked with a red frame. (B) IPTMnet database predicted the phosphorylation sites in MBNL1. (C) The iGPS database predicted that CDK12 phosphorylated MBNL1 through multiple sites. (D) In vitro kinase assay was conducted after MBNL1 T6 phosphorylation site mutated. (E) Western blot detected the effects of CDK12(+), MBNL1-WT and MBNL1-mut on MBNL1 and p-MBNL1 protein expression. Data are presented as mean±SD (n=3). ^*^*P*<0.05, compared with CDK12(+)NC group. ^#^*P*<0.05, compared with CDK12(+) group; ^△^*P*<0.05, compared with CDK12(+)+MBNL1-WT group.

Figure S5 Effects of CDK12 and MBNL1 on VM formation in GBM cells. (A) The effects of CDK12 and MBNL1 on the proliferation of U251 and U373 cells were detected by CCK8 assay. Data are presented as mean±SD (n=3). ^*^*P*<0.05, compared with the Control group. ^#^*P*<0.05, compared with CDK12(-)+MBNL1(+)NC group; ^△^*P*<0.05, compared with CDK12(-)+MBNL1(-)NC group. (B) Hstudio M4 detected the effect of CDK12 and MBNL1 on the migration of U251 and U373 cells (n=5). (C) Transwell assay was used to detect the effects of CDK12 and MBNL1 on the invasion of U251 and U373 cells. Data are presented as mean±SD (n=3). ^*^*P*<0.05, compared with the Control group. ^#^*P*<0.05, compared with CDK12(-)+MBNL1(+)NC group; ^△^*P*<0.05, compared with CDK12(-)+MBNL1(-)NC group. Scale bar: 50 μm. (D) Three-dimensional tube formation assay detected the effects of CDK12 and MBNL1 on the tube formation of U251 and U373 cells. Data are presented as mean±SD (n=3). ^*^*P*<0.05, compared with the Control group. ^#^*P*<0.05, compared with CDK12(-)+MBNL1(+)NC group; ^△^*P*<0.05, compared with CDK12(-)+MBNL1(-)NC group. Scale bar: 50 μm. (E) Western blot was used to detect the effects of CDK12 and MBNL1 on VM-related proteins MMP2 and LAMC2 expression in U251 and U373 cells. Data are presented as mean±SD (n=3). ^*^*P*<0.05, compared with the Control group. ^#^*P*<0.05, compared with CDK12(-)+MBNL1(+)NC group; ^△^*P*<0.05, compared with CDK12(-)+MBNL1(-)NC group.

Figure S6 The transfection efficiencies of METTL3, BUD13, CDK12 and MBNL1. (A) The transfection efficiency of METTL3 knockdown was detected by western blot. Data are presented as mean±SD (n=3, each group). ^*^*P*<0.05, ^**^*P*<0.01, compared with METTL3(-)NC group. (B) The transfection efficiency of BUD13 knockdown was detected by western blot. Data are presented as mean±SD (n=3, each group). ^*^*P*<0.05, ^**^*P*<0.01, compared with BUD13(-)NC group. (C) The transfection efficiency of CDK12 overexpression was detected by western blot. Data are presented as mean±SD (n=3, each group). ^**^*P*<0.01, compared with CDK12(+)NC group. (D) The transfection efficiency of CDK12 knockdown was detected by western blot. Data are presented as mean±SD (n=3, each group). ^*^*P*<0.05, ^**^*P*<0.01, compared with CDK12(-)NC group. (E) The transfection efficiency of MBNL1 overexpression was detected by western blot. Data are presented as mean±SD (n=3, each group). ^**^*P*<0.01, compared with MBNL1(+)NC group. (F) The transfection efficiency of MBNL1 knockdown was detected by western blot. Data are presented as mean±SD (n=3, each group). ^*^*P*<0.05, ^**^*P*<0.01, compared with MBNL1(-)NC group.

Supplementary Table

Table S1 Primers used for qRT-PCR

Table S2 The short hairpin RNAs and their sequences

Table S3 The sites mutation
